# Integrative Analysis of *PAIP2B* to Identify a Novel Biomarker for Pancreatic Ductal Adenocarcinoma

**DOI:** 10.1055/s-0043-1777789

**Published:** 2023-12-19

**Authors:** Yaoxian Xiang, Li Wang, Yurong Cheng, Huanjuan An, Chan Zhang, Jing Wang, Yingying Tong, Dong Yan

**Affiliations:** 1 Department of Oncology, Beijing Luhe Hospital Affiliated to Capital Medical University, Beijing, China; 2 Department of Hematology, Peking University Shougang Hospital, Beijing, China

**Keywords:** pancreatic carcinoma, *PAIP2B*, overall survival, prognosis, biomarker

## Abstract

The aim of the study was to evaluate the potential diagnostic and prognostic value of gene, Poly A-Binding Protein Interacting Protein 2B ( *PAIP2B* ) in pancreatic cancer. We used the gene expression data and clinical information of pancreatic adenocarcinoma patients from The Cancer Genome Atlas database and Gene Expression Omnibus database to analyze the expression of *PAIP2B* in pancreatic cancer samples, and validated the expression of *PAIP2B* in tumor tissue, using bioinformatics technology to explore the prognostic value of *PAIP2B* and its possible biological function. A significantly lower level of *PAIP2B* was observed in pancreatic cancer patients than in controls, and validated by immunohistochemistry. *PAIP2B* reduced the proliferation and invasion of cancer cells and had a significantly high expression in early stage. Patients with lower levels of *PAIP2B* had a significantly shorter median survival time than those with higher levels. DNA demethylation played an important role in *PAIP2B* expression. In addition, *PAIP2B* expression was significantly associated with the tumor-infiltrating immune cells, especially T cells CD8, T cells CD4 memory resting, macrophages M0, and dendritic cells resting. Our study also found that *PAIP2B* regulated miRNA function leading to disease progression in pancreatic cancer patients. Our study explored the potential value of *PAIP2B* as a biological link between prognosis and pancreatic cancer, and provided reference for the follow-up study on the role of *PAIP2B* in pancreatic cancer.

## Introduction

Pancreatic ductal adenocarcinoma (PDAC) is one of the most malignant tract tumors with a poor prognosis and an average 5-year survival rate of 8% due to late diagnosis, high invasiveness, less effective therapy, and profound drug-resistant nature of the tumor. 1 Despite the development of various treatments such as chemotherapy and targeted therapies in recent years, the prognosis of PDAC patients is still unsatisfactory. 2 Early diagnosis remains an unresolved problem, as a large proportion of patients are diagnosed at an advanced stage or with extensive metastases and about 80% of resected patients suffer relapse and die shortly after surgery. 3 Therefore, there is an urgent need for new molecular markers in the precise diagnosis of PDAC, in order to benefit the survival of patients by diagnosing them as early as possible.

In our previous study on genetic susceptibility to pancreatic cancer using the existing genome-wide association data, we identified a significant interaction of prognosis with single nucleotide variation (SNV) of the Poly A-Binding Protein Interacting Protein 2B ( *PAIP2B* ) gene variant rs113988120. 4 *PAIP2B* , a human PABP-interacting protein, is a translational inhibitor by reducing the poly (A)-binding protein (PABP) activity. 5 It is mainly involved in the translation process of mRNA and can be involved not only in regulating the rate of mRNA deoxygenation but also in causing the decay of mRNA, both of which are associated with the cancerous transformation of normal tissues. 6 7 Analysis based on The Cancer Genome Atlas (TCGA) database showed that the mRNA expression level of *PAIP2B* was significantly lower in pancreatic tumors than in normal tissues, which was consistent with our previous RNA-sequencing analysis. Therefore, further validation and functional studies on the expression of this gene will be important for understanding the biology of *PAIP2B* for pancreatic cancer to guide clinical decision-making.

In this study, we analyzed the biological functions of *PAIP2B* in the development of PDAC through multiomics data. First, we identified the differences in *PAIP2B* expression in tumor and normal tissues. Next, we analyzed the clinicopathological characteristic interaction with *PAIP2B* , evaluated the value of *PAIP2B* for early identifications, and assessment of prognosis of patients with PDAC. Then, we assessed the correlation between *PAIP2B* expression and the levels of tumor-infiltrating immune cells (TIICs) in the tumor microenvironment, and the response to the drug treatment in patients. Finally, we established a network of potential ceRNA associated with the development of PDAC. Our findings may contribute to investigate whether low *PAIP2B* expression could promote progression for pancreatic cancer and evaluate the potential values of a potential therapeutic target for PDAC.

## Materials and Methods

### Data Acquisition

We downloaded the mRNA expression profiles data and corresponding clinical information of 182 pancreatic adenocarcinoma (PAAD) patients from TCGA database ( https://tcga-data.nci.nih.gov/tcga/ ), which includes 178 pancreatic cancer samples and 4 paraneoplastic samples. Then, to exclude the samples with incomplete survival information, datum of 176 PDAC patients were finally included. Meanwhile, we downloaded miRNA expression profiles data of pancreatic cancer cohort, containing 179 tumor tissues and 4 paracancerous tissues. In addition, data of 167 normal pancreatic tissues were collected from genotype-tissue expression database. We also obtained the GSE16515 dataset from the Gene Expression Omnibus (GEO) database ( https://www.ncbi.nlm.nih.gov/geo/ ), which contains 36 pancreatic cancer samples and 16 normal pancreatic tissue samples.

### Expression and Survival Analysis

*PAIP2B* expression was compared between normal and multiple PDAC tissues. We estimated overall survival using the “Survival” in R package (4.2.0) and compared the overall survival of PDAC patients between the *PAIP2B* high- and low-expression groups using Kaplan–Meier curves. We defined a *p* -value <0.05 as statistically valid. Meanwhile, the alterations of *PAIP2B* gene in PDAC were detected from cBioPortal database ( https://www.cbioportal.org/ ), such as the copy number variation (CNV) profile of *PAIP2B* , and the correlation between the methylation level of DNA and gene expression. Furthermore, the online tool UALCAN ( http://ualcan.path.uab.edu/ ) was used to analyze the relationship between *PAIP2B* expression and clinicopathological characteristics.

### Identification of Differential Gene Expression and Functional Analysis

We used the “limma” package of R language 8 to explore differentially expressed mRNAs, miRNAs, and lncRNAs according to the criteria of Log2 fold-change |log2FC| > 2 and false discovery rate (FDR) < 0.05. Kyoto encyclopedia of genes and genomes (KEGG)/gene ontology (GO) enrichment analysis of differentially expressed genes was performed using the clusterProfiler R package. 9 The enrichment results were visualized with the “ggplot2” package in R software. STRING database ( https://string-db.org/version 11.0) 10 was used to develop a protein–protein interaction (PPI) network. Cytoscape (version 3.7.2) software 11 was utilized to visualize and analyze the PPI network.

### Immune-related Analysis and Drug Efficacy

The CIBERSORT algorithm was used to calculate the relative proportions of 22 immune cell types. In addition, the differences in TIICs between the *PAIP2B* high- and low-expression groups were compared by CIBERSORT software. 12 The immune scores were calculated by the “Estimate” R package ( https://R-Forge.R-project.org/projects/estimate/ ). R package of “OncoPredict” was used to predict efficacy and safety of a few drugs' correlation between *PAIP2B* expression. 13

### Immunohistochemical Staining

We collected clinical samples from PDAC patients participating in the study after obtaining informed consent from patients and ethical approval from medical authorities. The resected primary tumor specimens were fixed in 10% formalin and embedded in paraffin. The longest diameter of all tumor samples was measured and one or two 5-μm-thick sections were obtained from each centimeter of sample from areas containing viable tumor tissue with no (or minimal) hemorrhage or necrosis. Sections (5-μm thickness) of tissue specimens were obtained after antigen extraction by microwave. Immunostaining was performed with *PAIP2B* monoclonal antibody (Abcam, ab184774).

## Results

### PAIP2B Expression and Gene Variation

**Fig. 1:**
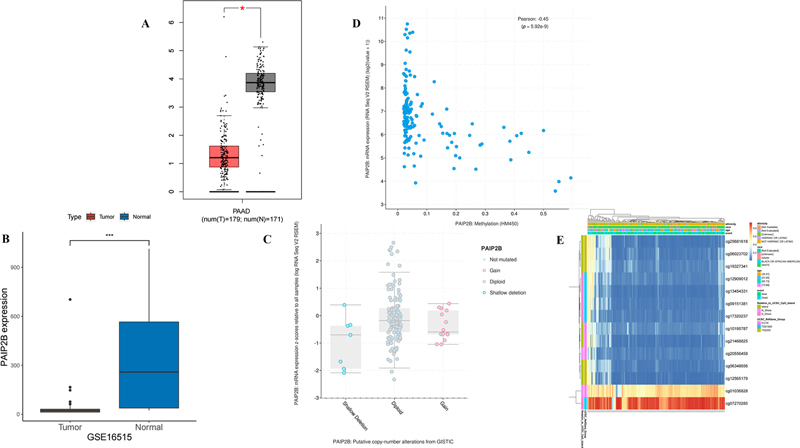
Expression of PAIP2B in tumor tissue and variation. ( **A** ) Gene PAIP2B expression in the GEPIA database. ( **B** ) Gene PAIP2B expression in the GSE16515 dataset. ( **C** ) Copy number variation of PAIP2B in the cBioPortal database. ( **D** ) Correlation of DNA methylation level of PAIP2B with its expression. ( **E** ) Methylation heat map at CG sites of PAIP2B gene. PAIP2B, poly A-binding protein interacting protein 2B; PAAD, pancreatic adenocarcinoma.

First, we compared the expression of *PAIP2B* between tumor tissue and normal tissue in PDAC cohort with the gene expression profiling interactive analysis (GEPIA) ( https://gepia.cancer-pku.cn/ ) database, and validated the outcome based on the GSE16515 database. Both datasets indicated that a significantly lower level of *PAIP2B* was observed in tumor tissue than in normal tissue ( Fig. 1A, B ). Second, we applied the cBioPortal database to analyze the frequencies of *PAIP2B* alteration in pancreatic cancer samples. The CNV profile of *PAIP2B* is shown in Fig. 1C . There is no significant association of copy number deletion and lower *PAIP2B* expression. Finally, we analyzed the correlation of *PAIP2B* between DNA methylation level and its expression, and the results showed that DNA methylation level in CpG of *PAIP2B* had a significantly negative correlation with its expression ( Fig. 1D ). In addition, we estimated methylation values at CG sites of *PAIP2B* gene, and found two CG sites (cg01036828 and cg07270285) that had higher methylation values than the others ( Fig. 1E ). Consequently, there is a strong possibility that correlations of methylation levels within a given CG site play a critical role in *PAIP2B* expression.

### Gene and Protein Network

**Fig. 2:**
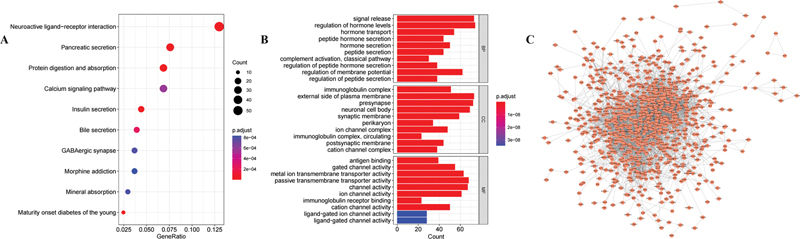
Analysis of gene–gene network and PPI network of PAIP2B. ( **A** ) The top 10 significantly enriched KEGG pathways. ( **B** ) Analysis of GO terms (BP, CC, MF) enrichment bar plot. ( **C** ) PPI network graph, nodes represent genes and the edges represent their interrelationships. PAIP2B, poly A-binding protein interacting protein 2B; PPI, protein–protein interaction; BP, biological process, CC, cellular component; MF, molecular function.

Patients of TCGA-PAAD cohort were divided into two groups according to *PAIP2B* expression levels. A total of 1,113 differentially expressed genes were identified between two groups on account of the criteria |log2FC| > 2 and FDR < 0.05. The gene–gene network and GO/KEGG enrichment analyses were performed. These results were presented in Fig. 2A, B . It is suggested that *PAIP2B* was a potential diagnostic and prognostic marker for pancreatic cancer. After that, we constructed the PPI network to evaluate the correlation of differentially expressed genes through STRING website, with minimum required interaction score > 0.4. This PPI network was visualized by Cytoscape software, and was shown in Fig. 2C .

### Correlation with Clinical Factors and Survival

Correlation analyses found that the level of *PAIP2B* expression was significantly associated with race, age, and TP53 gene mutation. *PAIP2B* expression levels were significantly higher in patients with TP53-Nonmutant compared with TP53-Mutant, similar to Caucasian versus Asian, African American versus Asian, and age (61–80 vs. 81–100 years). Otherwise, it showed a declining trend in advanced stage based on the UALCAN database ( Supplementary Fig. S1 ). Kaplan–Meier analysis certified that low levels of *PAIP2B* had a higher median survival time than those with high levels ( Supplementary Fig. S2A, B ). The results support that *PAIP2B* were a potential factor for prognosis.

### Immune-related Analysis

**Fig. 3:**
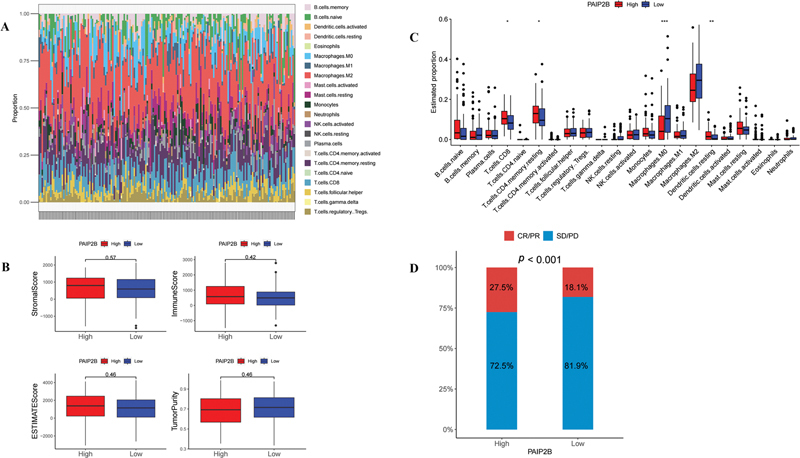
The relationship between PAIP2B expression and immune cells. ( **A** ) Tumor-infiltrating immune cells visualization. ( **B** ) Different immune cells' statistical analysis between high- and low-level PAIP2B expression group. ( **C** ) ImmuneScore, ESTIMATEScore, StromalScore, and TumorPurity compared with different PAIP2B expression. ( **D** ) Responses to immunotherapy displayed between high and low groups of PAIP2B. CR/PR, partial or complete response; PAIP2B, poly A-binding protein interacting protein 2B; PD/SD, progressive disease/stable disease.

In order to investigate whether *PAIP2B* expression play a crucial regulatory role in tumor microenvironment (TME), we explored the correlation between *PAIP2B* and TIICs base on the TCGA-PAAD cohort by the CIBERSORT algorithm ( Fig. 3A ). 12 We divided the patients into two groups based on PAIP2B levels and analyzed the differences in immune cell infiltration between the two groups. Four types of immune cells had significant difference between high levels of *PAIP2B* with low levels ( Fig. 3B ). No significant associations were found between the *PAIP2B* expression with immune scores, such as ESTIMATEScore, ImmuneScore, StromalScore, and TumorPurity ( Fig. 3C ). Notably, we found a higher proportion of patients with partial or complete response to immunotherapy in high level group of *PAIP2B* than low level group based on the IMvigor210 cohort ( Fig. 3D ).

### Correlation Analysis of *PAIP2B* Gene Expression and Drug Efficacy

Correlation analyses found that *PAIP2B* expression predicted efficacy and safety of a few drugs by “OncoPredict” package. 13 The results showed that level of *PAIP2B* was significantly negative correlation with 113 drugs such as OF.1_1853, Doramapimod_1042, ML323_1629, and TAF1_5496_1732, otherwise positive correlation with those such as Selumetinib_1736, ERK_6604_1714, ERK_2440_1713, and SCH772984_1564 ( *p* < 0.05, Supplementary Fig. S3A, B ). Then, we compared the IC _50_ levels of top four drugs between higher and lower levels of *PAIP2B* . The results showed that effectiveness of Selumetinib_1736 was lower in high level group of *PAIP2B* , and ML323_1629 was higher in high level group conversely ( Supplementary Fig. S3C, D ).

### Prediction of an LncRNA–PAIP2B–miRNA Axis

With the exception of DNA methylation, we also identified miRNA–mRNA target interactions by computational prediction through TargetScan database. Twenty-nine miRNAs targetting *PAIP2B* were obtained. Correlation analyses found that six miRNAs (hsa-miR-216a-3p, hsa-miR-377-3p, hsa-miR-128-3p, hsa-miR-299-3p, hsa-miR-488-3p, hsa-miR-491-5p) were significantly positively associated with *PAIP2B* expression and four miRNAs (hsa-miR-320a, hsa-miR-203a-3p.2, hsa-miR-27a-3p, hsa-miR-21-5p) negative correlation with its expression profile of TCGA-PAAD cohort ( Supplementary Fig. S4A ). The volcano maps of differential lncRNA and miRNA in PDAC were shown in Supplementary Fig. S4B, C . We also established “LncRNA– *PAIP2B* –miRNA” module on LncACTdb 3.0 database ( http://bio-bigdata.hrbmu.edu.cn/LncACTdb/index.html ; Supplementary Fig. S4D, E ). 14

### Immunohistochemical Staining

**Fig. 4:**
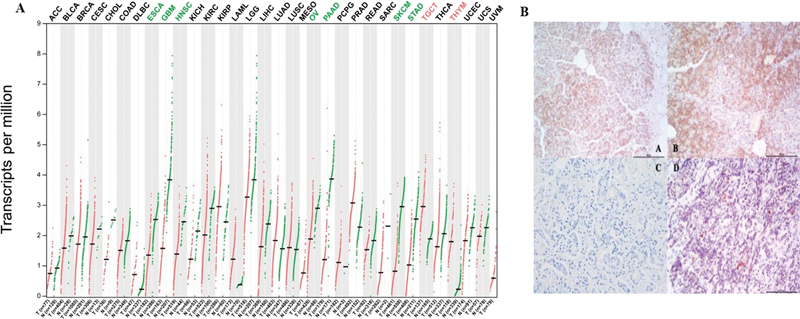
PAIP2B expression. ( **A** ) PAIP2B expression in different tumor species base on TCGA. ( **B** ) PAIP2B expression in pancreatic tumor tissue. ( **B-A** ) Weakly positive PAIP2B in pancreatic cystadenoma. ( **B-B** ) Positive PAIP2B in pancreatic normal tissue. ( **B-C** ) Negative expression in control specimens. ( **B-D** ) Negative PAIP2B expression in pancreatic tumor tissue. PAIP2B, poly A-binding protein interacting protein 2B; TCGA, The Cancer Genome Atlas.

*PAIP2B* expression in different tumor species based on TCGA was shown in Fig. 4A . Furthermore, a total of 97 human pancreatic cancer specimens were obtained. *PAIP2B* -negative expression was observed in tumors examined using immunohistochemical (IHC) staining, and weakly positive expression showed in paracancerous tissue. Examples of the IHC staining patterns are shown in Fig. 4B .

## Discussion

Pancreatic cancer is a malignant tumor with high heterogeneity, mainly manifested by a high rate of genetic mutations, local invasion of the tumor stroma, and distant metastasis. 15 Several recent studies have constructed a genetic map of pancreatic disease by performing a comprehensive genetic analysis of pancreatic cancer, which in turn has confirmed that it is a genetic disease. 16 17 Therefore, identifying as many important molecules and proteins involved in the development and progression of pancreatic cancer as possible will help identify new potential therapeutic targets. In previous study, a genome-wide association study of 868 pancreatic cancer patients, we discovered a genome-wide significant SNV of the *PAIP2B* gene variant rs113988120 which had a 3.06-fold higher risk of death (95% confidence interval: 2.10–4.47, *p* = 6.4 × 10 ^−9^ ). 4 *PAIP2B* is a translational inhibitor as well as an antiproliferative factor. 18 Currently, the impact of *PAIP2B* on pancreatic cancer is uncertain.

Previous studies showed that *PAIP2B* is a translational inhibitor. 19 It regulates the PABP activity. PABP can act on translation initiation factor eukaryotic translation initiation factor 4 gamma 1 and poly(A) tail to cause mRNA cyclization, thus enhancing translation. 20 *PAIP2B* is a strong regulator of vascular endothelial growth factor, 21 and it is an antiproliferative factor. 22 Therefore, *PAIP2B* is important to keep balance of protein levels in vivo. Berlanga et al report that *PAIP2B* is mainly expressed in the pancreas, which may suggest roles in glucose homeostasis. 19 In this study, we completely revealed the expression, mutation, prognosis factor, DNA methylation, and associations with immune cell infiltration and response to checkpoint inhibitors of *PAIP2B* in PAAD cohort. First, we demonstrated that *PAIP2B* was down-regulated in pancreatic tumors compared with nontumor tissues both in the GEPIA database and the GSE16515 database, then validated the result in tumor tissue of pancreatic cancer patients by IHC staining and RNA-Seq. Moreover, *PAIP2B* associated with the prognosis of PDAC has further been evaluated in clinical settings. Our study showed a shorter survival linked to lower *PAIP2B* expression in TCGA database. This suggested that *PAIP2B* may be a novel tumor suppressor, and identified as a predictor of survival. We evaluated the genetic alterations of *PAIP2B* in PAAD and shows that DNA methylation plays a vital role in negative regulation with *PAIP2B* expression.

In order to illuminate the biological role of *PAIP2B* in the TCGA RNA-sequencing dataset for PAAD, we identified differential gene expression (DEG) between groups with high and low *PAIP2B* expression, and performed functional enrichment analysis. The results of GO and KEGG enrichment analysis on DEG are shown in Supplementary Table S1 . Those were related to metabolic pathways, for example, insulin secretion, cellular processes, protein digestion and absorption, and so on. The function and expression of *PAIP2B* were provided as same as recently reported by Mukherjee and Goswami. 7 Obviously, *PAIP2B* was involved in tumor progression and regarded as prognosis factor of pancreatic cancer patients. Furthermore, the immune landscape of *PAIP2B* expression was successfully analyzed in PAAD cohort. The proportions of macrophages were significantly higher in low levels group of *PAIP2B* , whereas those of T cells CD8, T cells CD4 memory resting, and dendritic cells resting demonstrated increased trend in high levels group. No correlation was detected among *PAIP2B* expression and TME scores. It is well known that *PAIP2B* was identified as a potential immunomodulatory factor and plays an important role in maintaining TIICs. 18

Recent research reported that Kirsten rat sarcoma viral oncogene (KRAS) perturbation causes lower *PAIP2B* expression in lung tissue, but not in breast, kidney, and prostate tissues. 23 As is known to all, 95% of PDACs can be accompanied by mutations in the *KRAS* gene, which is therefore a recognized driver of PDAC growth and maintenance. In our study, we demonstrated that *PAIP2B* expression was general lower in tumor than control. A phase II study (NCT03040986) showed that patients with advanced pancreatic cancer harboring somatic KRASG12R mutation were more sensitive to Selumetinib, a mitogen-activated protein kinase 1 and 2 inhibitor, 24 similar to our study reported. In addition, previous studies have evidenced that PAIP2 enhanced poly(A)-shortening of miRNA-targeted mRNAs and regulated miRNA function. 25 26 Here, we also demonstrate that *PAIP2B* may be involved in the regulation of ceRNA modules to influence PDAC aggressiveness and prognosis.

However, our study still has some limitations. Firstly, we included a limited sample size of patients with pancreatic cancer, and secondly, the information from the databases we used may be potentially biased, so the generalizability of the conclusions we obtained to the whole population may be limited. Therefore, we call for further large-sample, multiethnic pancreatic cancer patient cohort-based studies in the future, and our study will also provide a reference for further clarification of the diagnostic and prognostic value of PAIP2B in pancreatic cancer in the future.

## Conclusions

*PAIP2B* has an important biological function in the development of pancreatic cancer. Further studies on the molecular mechanism of PDAC development are needed to promote its precise diagnosis and treatment.
