# Supplementary material for: Integrative Analysis of PAIP2B to Identify a Novel Biomarker for Pancreatic Ductal Adenocarcinoma
Source: Glob Med Genet. 2023 Dec 19;10(4):388–94. doi: 10.1055/s-0043-1777789 (PMC10730282; doi:10.1055/s-0043-1777789)
Supplement: Supplementary file 1 — Supplementary Material [file 10-1055-s-0043-1777789-s2300086.pdf]

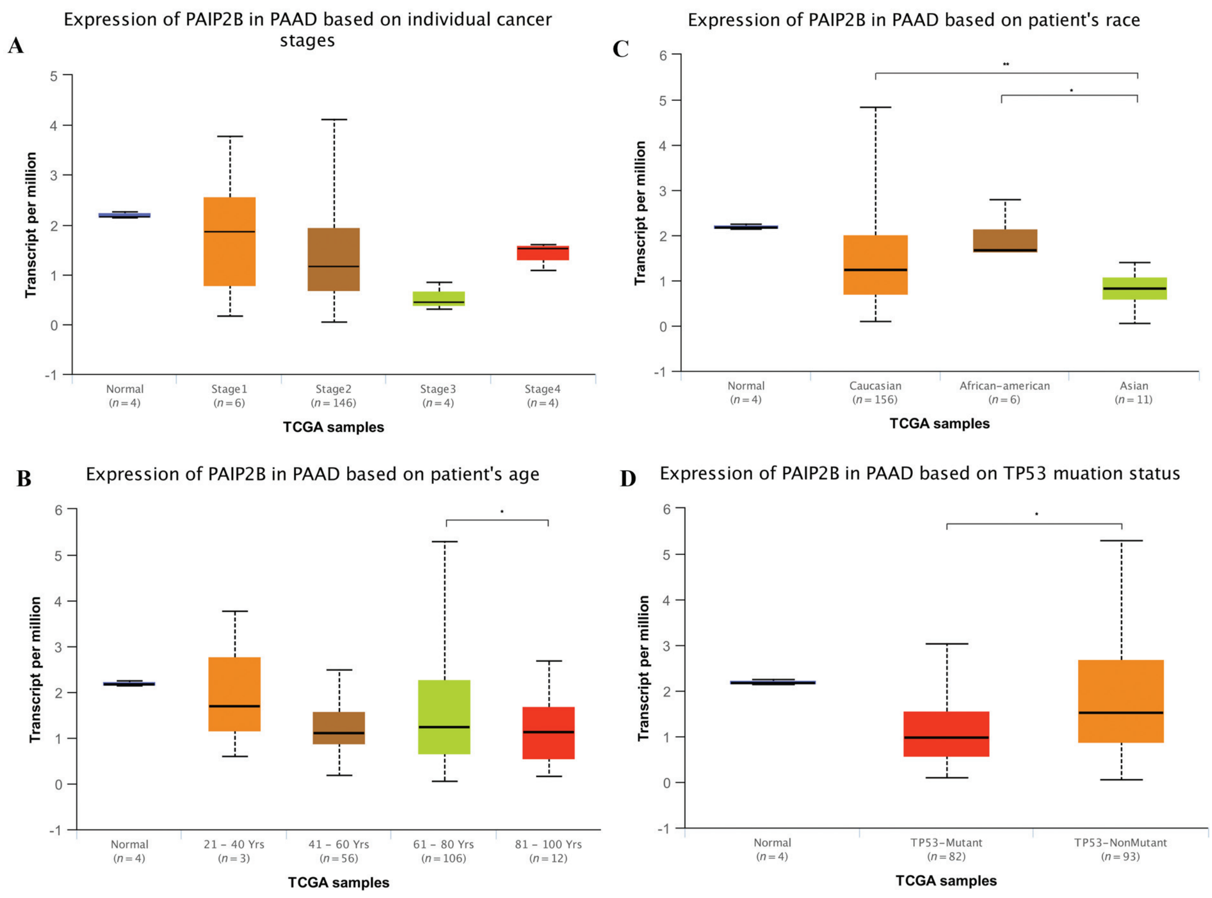

Supplementary Fig. S1 Correlation between PAIP2B expression and clinical factors. (**A**) PAIP2B expression in different tumor stages based on UALCAN database. (**B**) PAIP2B expression at different ages. (**C**) PAIP2B expression in different races. (**D**) Correlation between PAIP2B expression and TP53 mutation. PAAD, pancreatic adenocarcinoma; PAIP2B, poly A-binding protein interacting protein 2B; TCGA, The Cancer Genome Atlas.

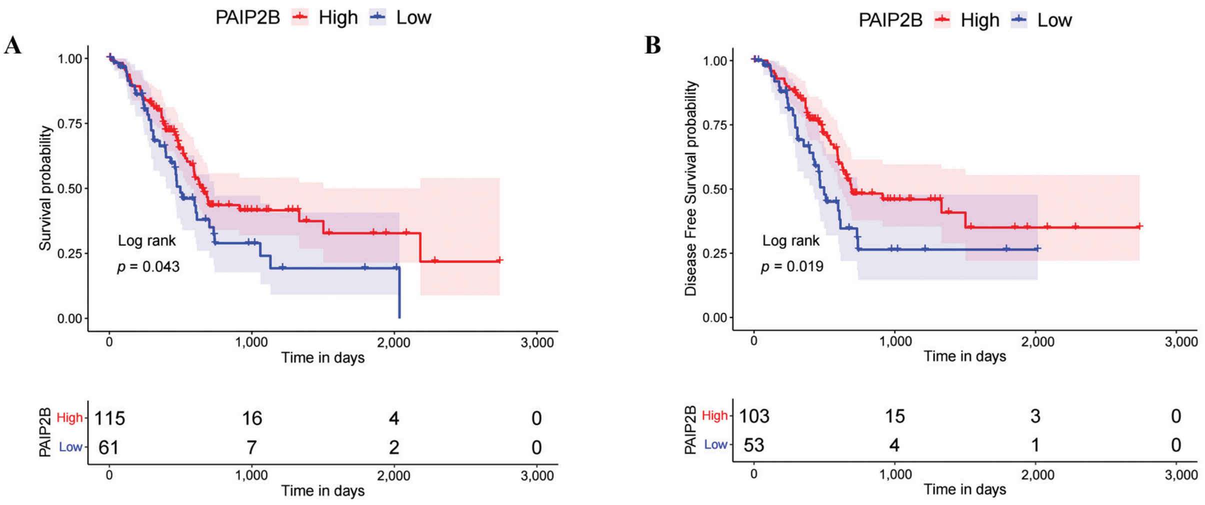

Supplementary Fig. S2 Correlation with PAIP2B expression and Survival by Kaplan–Meier curve. (**A**) OS curve between high level and low level group of PAIP2B expression. (**B**) DFS curve between high level and low level group of PAIP2B expression, *p*-value is based on log-rank test. PAIP2B, poly A-binding protein interacting protein 2B; OS, overall survival; DFS, disease-free survival.

 © 2023. The Author(s).

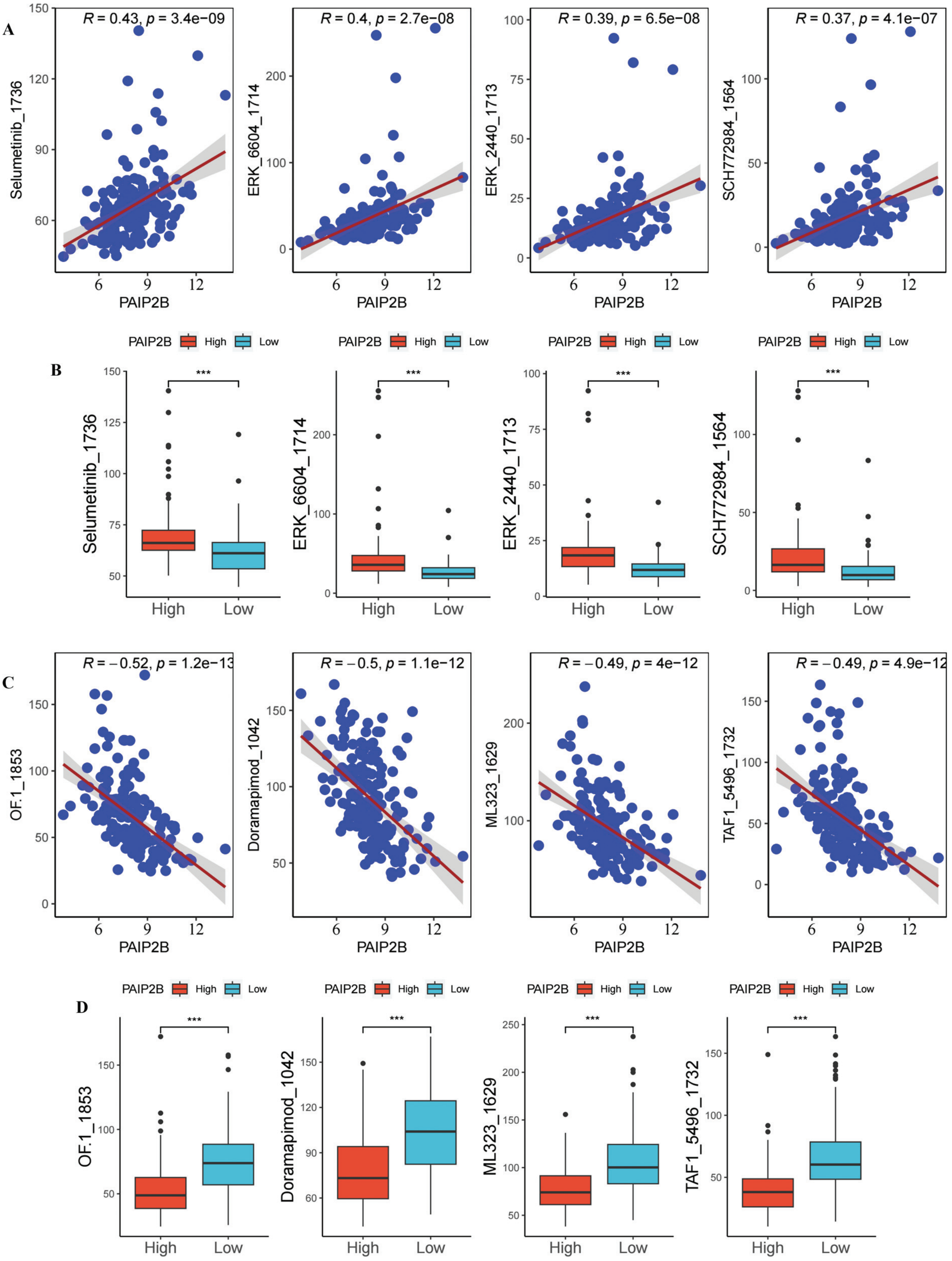

Supplementary Fig. S3 Correlation between drug efficacy and PAIP2B expression. (**A, C**) Correlation between the IC_{50} value of drugs and PAIP2B. (**B, D**) IC_{50} value of different drugs between high- and low-level groups. PAIP2B, poly A-binding protein interacting protein 2B.

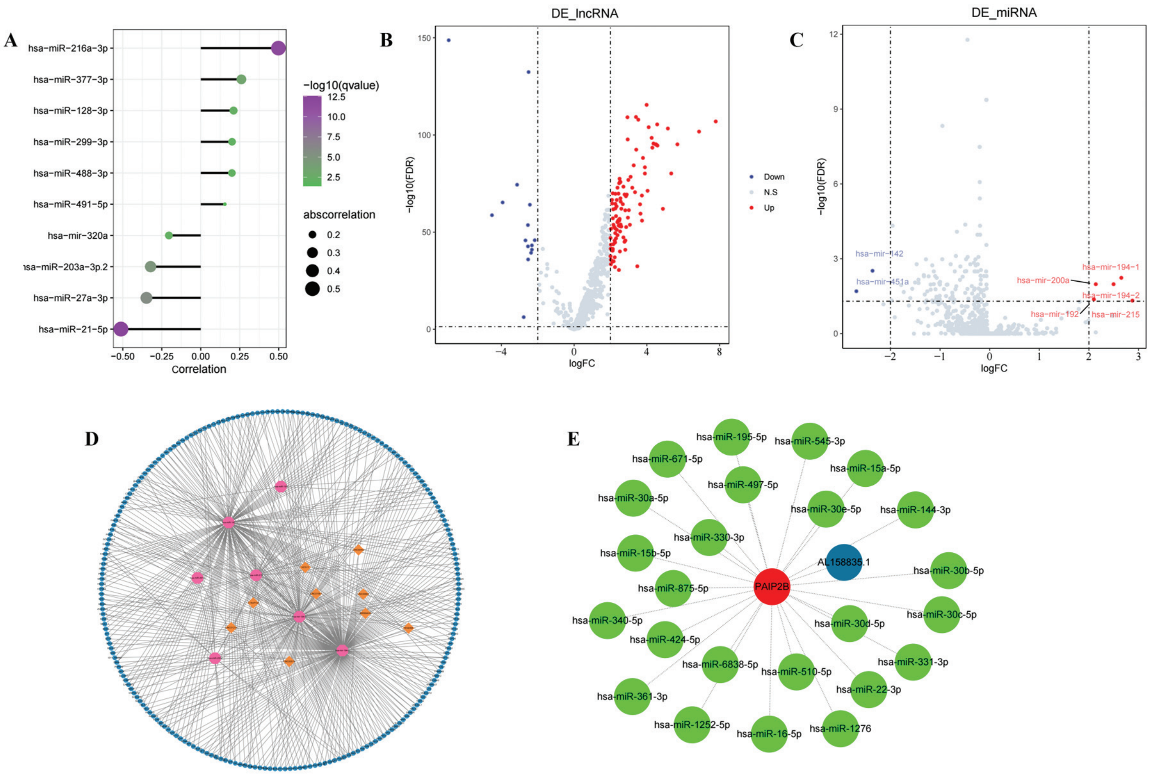

Supplementary Fig. S4 ceRNA network prediction. (**A**) Correlation of miRNA with PAIP2B expression. (**B**) Volcano map of differential lncRNA in PDAC. (**C**) Volcano map of differential miRNA in PDAC. (**D**) ceRNA (lncRNA–miRNA–mRNA) network. (**E**) lncRNA–PAIP2B–miRNA axis. PAIP2B, poly A-binding protein interacting protein 2B; PDAC, pancreatic ductal adenocarcinoma.

 © 2023. The Author(s).

Supplementary Table S1 KEGG and GO enrichment analyses

Ontology	ID	Description	GeneRatio	BgRatio	p-Value	*p*.adjust	q-Value	geneID	Count
BP	GO:0023061	Signal release	73/917	451/18,800	8.32E-20	3.85E-16	3.31E-16	*MYRIP/BRSK2/CELA2A/CHRNB3/P2RX1/SYT6/NLGN1/KCNA2/GPLD1/VIP/ABAT/ADCY1/CARTPT/TACR1/CACNB2/KCNB1/SLC6A4/NRXN1/PCLO/GALR1/CLTRN/CHRNB2/GHRL/ADCYAP1/GRIK5/SYT4/CRHBP/GPR27/SFRP1/UNC13A/CADPS/LRRK2/NKX6-1/KCNJ11/SPINK1/SYP/SOX11/NEUROD1/SYT9/CPE/NR0B2/KCNA5/CXCL12/CNR1/RIMS2/SNAP25/RIMS1/PRKCB/CASR/RFX6/ZBED6/ABCC8/TRPV6/SCG5/FGA/CPLX2/EPHA5/CFTR/GCK/SYT5/NR1H4/ADIPOQ/FGB/CHGA/PTPRN/G6PC2/SLC30A8/FFAR1/UCN3/GRM4/SYT8/BLK/FGG*	73
BP	GO:0010817	Regulation of hormone levels	74/917	496/18,800	5.32E-18	1.23E-14	1.06E-14	*MYRIP/BRSK2/CELA2A/SCNN1B/GCNT4/PNLIP/MBOAT4/GPLD1/CHST9/CMA1/VIP/ABAT/CARTPT/TACR1/KCNB1/ADH1A/SLCO1C1/PCLO/GALR1/CLTRN/GHRL/ADCYAP1/ADH1B/CTSG/CRHBP/GPR27/SFRP1/SERPINA7/NKX6-1/KCNJ11/HPN/SPINK1/PCSK1/UGT2B15/SOX11/NEUROD1/SYT9/TPO/PRLHR/SLC22A9/CPE/NR0B2/KCNA5/UGT2B11/CYP2C8/CNR1/RIMS2/DUOXA1/SNAP25/PCSK2/CASR/RFX6/ZBED6/ABCC8/TRPV6/SCG5/FGA/EPHA5/CFTR/GCK/NR1H4/ADIPOQ/FGB/CHGA/BMP5/TTR/PTPRN/G6PC2/UGT2B17/SLC30A8/FFAR1/UCN3/BLK/FGG*	74
BP	GO:0009914	Hormone transport	54/917	290/18,800	1.16E-17	1.78E-14	1.53E-14	*MYRIP/BRSK2/CELA2A/GPLD1/VIP/ABAT/CARTPT/TACR1/KCNB1/SLCO1C1/PCLO/GALR1/CLTRN/GHRL/ADCYAP1/CRHBP/GPR27/SFRP1/SERPINA7/NKX6-1/KCNJ11/SPINK1/SOX11/NEUROD1/SYT9/SLC22A9/CPE/NR0B2/KCNA5/CNR1/RIMS2/SNAP25/CASR/RFX6/ZBED6/ABCC8/TRPV6/SCG5/FGA/EPHA5/CFTR/GCK/NR1H4/ADIPOQ/FGB/CHGA/TTR/PTPRN/G6PC2/SLC30A8/FFAR1/UCN3/BLK/FGG*	54
BP	GO:0030072	Peptide hormone secretion	44/917	220/18,800	8.26E-16	9.56E-13	8.22E-13	*MYRIP/BRSK2/CELA2A/GPLD1/VIP/ABAT/CARTPT/KCNB1/PCLO/CLTRN/GHRL/ADCYAP1/CRHBP/GPR27/SFRP1/NKX6-1/KCNJ11/SPINK1/NEUROD1/SYT9/CPE/NR0B2/KCNA5/CNR1/RIMS2/SNAP25/CASR/RFX6/ZBED6/ABCC8/FGA/EPHA5/CFTR/GCK/NR1H4/FGB/CHGA/PTPRN/G6PC2/SLC30A8/FFAR1/UCN3/BLK/FGG*	44
BP	GO:0046879	Hormone secretion	50/917	281/18,800	1.30E-15	1.20E-12	1.03E-12	*MYRIP/BRSK2/CELA2A/GPLD1/VIP/ABAT/CARTPT/TACR1/KCNB1/PCLO/GALR1/CLTRN/GHRL/ADCYAP1/CRHBP/GPR27/SFRP1/NKX6-1/KCNJ11/SPINK1/SOX11/NEUROD1/SYT9/CPE/NR0B2/KCNA5/CNR1/RIMS2/SNAP25/CASR/RFX6/ZBED6/ABCC8/TRPV6/SCG5/FGA/EPHA5/CFTR/GCK/NR1H4/ADIPOQ/FGB/CHGA/PTPRN/G6PC2/SLC30A8/FFAR1/UCN3/BLK/FGG*	50

(*Continued*)

 © 2023. The Author(s).

Supplementary Table S1 (*Continued*)

Ontology	ID	Description	GeneRatio	BgRatio	p-Value	*p*.adjust	q-Value	geneID	Count
BP	GO:0002790	Peptide secretion	44/917	225/18,800	1.96E-15	1.51E-12	1.30E-12	*MYRIP/BRSK2/CELA2A/GPLD1/VIP/ABAT/CARTPT/KCNB1/PCLO/CLTRN/GHRL/ADCYAP1/CRHBP/GPR27/SFRP1/NKX6-1/KCNJ11/SPINK1/NEUROD1/SYT9/CPE/NR0B2/KCNA5/CNR1/RIMS2/SNAP25/CASR/RFX6/ZBED6/ABCC8/FGA/EPHA5/CFTR/GCK/NR1H4/FGB/CHGA/PTPRN/G6PC2/SLC30A8/FFAR1/UCN3/BLK/FGG*	44
BP	GO:0006958	Complement activation, classical pathway	30/917	108/18,800	3.21E-15	2.12E-12	1.83E-12	*C5/CLU/C6/C7/C8A/IGHV3-64/C4BPA/IGHV6-1/IGHV2-5/IGHV3-74/IGHV4-4/IGHV3-20/IGHV3-7/IGHV3-15/IGHV1-45/IGHV3-72/IGHV3-48/APCS/IGHV2-26/IGHV3-49/IGHV4-28/IGHV5-51/CRP/IGHV1-69/IGHV4-39/IGHV4-59/IGHA2/IGHV3-21/IGHV3-13/IGHV1-18*	30
BP	GO:0090276	Regulation of peptide hormone secretion	38/917	177/18,800	7.08E-15	4.09E-12	3.52E-12	*MYRIP/BRSK2/CELA2A/GPLD1/ABAT/CARTPT/KCNB1/GHRL/ADCYAP1/CRHBP/GPR27/SFRP1/NKX6-1/KCNJ11/SPINK1/NEUROD1/SYT9/NR0B2/KCNA5/CNR1/SNAP25/CASR/RFX6/ZBED6/ABCC8/FGA/EPHA5/CFTR/GCK/NR1H4/FGB/CHGA/G6PC2/SLC30A8/FFAR1/UCN3/BLK/FGG*	38
BP	GO:0042391	Regulation of membrane potential	62/917	425/18,800	8.54E-15	4.39E-12	3.78E-12	*NRCAM/CHRNB3/KCNK3/NTRK2/P2RX1/KCNJ5/NLGN1/KCNA2/AKAP6/BNIP3/RELN/FGF12/CD36/GABRB3/ABAT/STOX1/KCNH7/SLC8A3/SLC8A2/TACR1/SCN3A/DMD/CACNB2/KCNB1/SLC8A1/NRXN1/SCN4A/KCND3/SLC4A4/CHRNB2/CELF4/GHRL/GRIK5/MAPT/ANK2/LRRK2/RGS7BP/KCNJ11/SCN3B/SCN7A/MAPK8IP2/GLRA3/CBLN1/ATP1A3/KCNA5/KCNJ3/KCNMB2/HCN4/CNR1/RIMS2/RIMS1/MYOC/GABRA4/GABRG2/SEZ6/KCNH6/CFTR/GJD2/GLRA1/KCNK16/GABRA2/UCN3*	62
BP	GO:0002791	Regulation of peptide secretion	38/917	180/18,800	1.26E-14	5.47E-12	4.71E-12	*MYRIP/BRSK2/CELA2A/GPLD1/ABAT/CARTPT/KCNB1/GHRL/ADCYAP1/CRHBP/GPR27/SFRP1/NKX6-1/KCNJ11/SPINK1/NEUROD1/SYT9/NR0B2/KCNA5/CNR1/SNAP25/CASR/RFX6/ZBED6/ABCC8/FGA/EPHA5/CFTR/GCK/NR1H4/FGB/CHGA/G6PC2/SLC30A8/FFAR1/UCN3/BLK/FGG*	38
BP	GO:0002455	Humoral immune response mediated by circulating immunoglobulin	31/917	121/18,800	1.30E-14	5.47E-12	4.71E-12	*C5/CLU/C6/C7/C8A/IGHV3-64/PTPRC/C4BPA/IGHV6-1/IGHV2-5/IGHV3-74/IGHV4-4/IGHV3-20/IGHV3-7/IGHV3-15/IGHV1-45/IGHV3-72/IGHV3-48/APCS/IGHV2-26/IGHV3-49/IGHV4-28/IGHV5-51/CRP/IGHV1-69/IGHV4-39/IGHV4-59/IGHA2/IGHV3-21/IGHV3-13/IGHV1-18*	31

 © 2023. The Author(s).

Supplementary Table S1 (*Continued*)

Ontology	ID	Description	GeneRatio	BgRatio	p-Value	*p*.adjust	q-Value	geneID	Count
BP	GO:0006956	Complement activation	32/917	131/18,800	2.10E-14	7.86E-12	6.76E-12	*C5/CLU/C6/C7/FCN2/C8A/IGHV3-64/C4BPA/IGHV6-1/IGHV2-5/IGHV3-74/IGHV4-4/IGHV3-20/KRT1/IGHV3-7/IGHV3-15/IGHV1-45/IGHV3-72/IGHV3-48/APCS/IGHV2-26/IGHV3-49/IGHV4-28/IGHV5-51/CRP/IGHV1-69/IGHV4-39/IGHV4-59/IGHA2/IGHV3-21/IGHV3-13/IGHV1-18*	32
BP	GO:0090087	Regulation of peptide transport	38/917	183/18,800	2.21E-14	7.86E-12	6.76E-12	*MYRIP/BRSK2/CELA2A/GPLD1/ABAT/CARTPT/KCNB1/GHRL/ADCYAP1/CRHBP/GPR27/SFRP1/NKX6-1/KCNJ11/SPINK1/NEUROD1/SYT9/NR0B2/KCNA5/CNR1/SNAP25/CASR/RFX6/ZBED6/ABCC8/FGA/EPHA5/CFTR/GCK/NR1H4/FGB/CHGA/G6PC2/SLC30A8/FFAR1/UCN3/BLK/FGG*	38
BP	GO:0046883	Regulation of hormone secretion	43/917	231/18,800	2.55E-14	8.44E-12	7.26E-12	*MYRIP/BRSK2/CELA2A/GPLD1/ABAT/CARTPT/TACR1/KCNB1/GALR1/GHRL/ADCYAP1/CRHBP/GPR27/SFRP1/NKX6-1/KCNJ11/SPINK1/SOX11/NEUROD1/SYT9/NR0B2/KCNA5/CNR1/SNAP25/CASR/RFX6/ZBED6/ABCC8/SCG5/FGA/EPHA5/CFTR/GCK/NR1H4/ADIPOQ/FGB/CHGA/G6PC2/SLC30A8/FFAR1/UCN3/BLK/FGG*	43
BP	GO:0035592	Establishment of protein localization to extracellular region	54/917	351/18,800	5.30E-14	1.63E-11	1.41E-11	*SEL1L/MYRIP/BRSK2/PLA2G1B/CELA2A/GPLD1/RAB3C/VIP/ABAT/CARTPT/KCNB1/PCLO/CLTRN/GHRL/ADCYAP1/SYT4/GPR27/SFRP1/NKX6-1/KCNJ11/PCSK1/STXBP5L/AFM/NEUROD1/SYT9/CBLN1/NR0B2/KCNA5/CNR1/RIMS2/SNAP25/CASR/RFX6/ZBED6/SCG2/ABCC8/FGA/CBLN4/EPHA5/CFTR/GCK/NR1H4/IGF1/FGB/CHGA/PTPRN/G6PC2/SLC30A8/PPY/MTTP/FFAR1/UCN3/BLK/FGG*	54
BP	GO:0030073	Insulin secretion	37/917	179/18,800	5.65E-14	1.64E-11	1.41E-11	*MYRIP/BRSK2/CELA2A/GPLD1/ABAT/CARTPT/KCNB1/PCLO/CLTRN/GHRL/ADCYAP1/GPR27/SFRP1/NKX6-1/KCNJ11/NEUROD1/SYT9/NR0B2/KCNA5/CNR1/RIMS2/SNAP25/CASR/RFX6/ZBED6/ABCC8/EPHA5/CFTR/GCK/NR1H4/CHGA/PTPRN/G6PC2/SLC30A8/FFAR1/UCN3/BLK*	37
BP	GO:0015833	Peptide transport	44/917	249/18,800	8.77E-14	2.39E-11	2.05E-11	*MYRIP/BRSK2/CELA2A/GPLD1/VIP/ABAT/CARTPT/KCNB1/PCLO/CLTRN/GHRL/ADCYAP1/CRHBP/GPR27/SFRP1/NKX6-1/KCNJ11/SPINK1/NEUROD1/SYT9/CPE/NR0B2/KCNA5/CNR1/RIMS2/SNAP25/CASR/RFX6/ZBED6/ABCC8/FGA/EPHA5/CFTR/GCK/NR1H4/FGB/CHGA/PTPRN/G6PC2/SLC30A8/FFAR1/UCN3/BLK/FGG*	44

(*Continued*)

 © 2023. The Author(s).

Supplementary Table S1 (*Continued*)

Ontology	ID	Description	GeneRatio	BgRatio	p-Value	*p*.adjust	q-Value	geneID	Count
BP	GO:0071692	Protein localization to extracellular region	54/917	359/18,800	1.36E-13	3.50E-11	3.01E-11	*SEL1L/MYRIP/BRSK2/PLA2G1B/CELA2A/GPLD1/RAB3C/VIP/ABAT/CARTPT/KCNB1/PCLO/CLTRN/GHRL/ADCYAP1/SYT4/GPR27/SFRP1/NKX6-1/KCNJ11/PCSK1/STXBP5L/AFM/NEUROD1/SYT9/CBLN1/NR0B2/KCNA5/CNR1/RIMS2/SNAP25/CASR/RFX6/ZBED6/SCG2/ABCC8/FGA/CBLN4/EPHA5/CFTR/GCK/NR1H4/IGF1/FGB/CHGA/PTPRN/G6PC2/SLC30A8/PPY/MTTP/FFAR1/UCN3/BLK/FGG*	54
BP	GO:0009306	Protein secretion	53/917	350/18,800	1.75E-13	4.27E-11	3.67E-11	*SEL1L/MYRIP/BRSK2/PLA2G1B/CELA2A/GPLD1/RAB3C/VIP/ABAT/CARTPT/KCNB1/PCLO/CLTRN/GHRL/ADCYAP1/SYT4/GPR27/SFRP1/NKX6-1/KCNJ11/PCSK1/STXBP5L/NEUROD1/SYT9/CBLN1/NR0B2/KCNA5/CNR1/RIMS2/SNAP25/CASR/RFX6/ZBED6/SCG2/ABCC8/FGA/CBLN4/EPHA5/CFTR/GCK/NR1H4/IGF1/FGB/CHGA/PTPRN/G6PC2/SLC30A8/PPY/MTTP/FFAR1/UCN3/BLK/FGG*	53
BP	GO:0050796	Regulation of insulin secretion	32/917	147/18,800	6.45E-13	1.47E-10	1.27E-10	*MYRIP/BRSK2/CELA2A/GPLD1/ABAT/CARTPT/KCNB1/GHRL/GPR27/SFRP1/NKX6-1/KCNJ11/NEUROD1/SYT9/NR0B2/KCNA5/CNR1/SNAP25/CASR/RFX6/ZBED6/ABCC8/EPHA5/CFTR/GCK/NR1H4/CHGA/G6PC2/SLC30A8/FFAR1/UCN3/BLK*	32
BP	GO:0006959	Humoral immune response	49/917	317/18,800	6.68E-13	1.47E-10	1.27E-10	*C5/PLA2G1B/CLU/REG3G/C6/REG1B/REG3A/C7/PRSS2/FCN2/REG1A/CTSG/CCR2/C8A/IGHV3-64/PTPRC/C4BPA/FGA/IGHV6-1/IGHV2-5/IGHV3-74/SH2D1A/PRSS3/IGHV4-4/FGB/IGHV3-20/KRT1/IGHV3-7/IGHV3-15/IGHV1-45/CXCL9/JCHAIN/IGHV3-72/IGHV3-48/APCS/IGHV2-26/IGHV3-49/IGHV4-28/MS4A1/IGHV5-51/CRP/IGHV1-69/IGHV4-39/PAX5/IGHV4-59/IGHA2/IGHV3-21/IGHV3-13/IGHV1-18*	49
BP	GO:0042886	Amide transport	46/917	287/18,800	9.21E-13	1.94E-10	1.66E-10	*MYRIP/BRSK2/CELA2A/ABCB1/GPLD1/VIP/ABAT/CARTPT/KCNB1/PCLO/CLTRN/GHRL/ADCYAP1/CRHBP/GPR27/SFRP1/NKX6-1/KCNJ11/SPINK1/NEUROD1/SYT9/CPE/NR0B2/KCNA5/CNR1/RIMS2/SNAP25/CASR/RFX6/ZBED6/ABCC8/FGA/EPHA5/CFTR/GCK/NR1H4/FGB/CHGA/PTPRN/G6PC2/SLC30A8/MTTP/FFAR1/UCN3/BLK/FGG*	46
BP	GO:0006910	Phagocytosis, recognition	26/917	102/18,800	2.02E-12	4.06E-10	3.49E-10	*CD36/FCN2/IGHV3-64/C4BPA/IGHV6-1/IGHV2-5/IGHV3-74/IGHV4-4/IGHV3-20/IGHV3-7/IGHV3-15/IGHV1-45/IGHV3-72/IGHV3-48/IGHV2-26/IGHV3-49/IGHV4-28/IGHV5-51/CRP/IGHV1-69/IGHV4-39/IGHV4-59/IGHA2/IGHV3-21/IGHV3-13/IGHV1-18*	26

Global Medical Genetics © 2023. The Author(s).

Supplementary Table S1 (*Continued*)

Ontology	ID	Description	GeneRatio	BgRatio	p-Value	*p*.adjust	q-Value	geneID	Count
BP	GO:0072503	Cellular divalent inorganic cation homeostasis	63/917	494/18,800	2.54E-12	4.90E-10	4.21E-10	*PLA2G1B/KCNK3/MT1F/CTRC/P2RX1/CX3CR1/NOS1/AKAP6/BNIP3/CD36/RGN/EPO/SLC8A3/SLC8A2/TACR1/DMD/SLC30A2/CACNB2/RIC3/SLC8A1/PTGFR/MT1G/FASLG/GALR1/CYSLTR1/GHRL/ADCYAP1/ATP2B2/ANK2/CCR2/MCOLN3/CCKBR/SPINK1/CCL14/GPR174/SCGN/CXCR6/CACNA1B/CCR5/AVPR1B/KCNA5/CXCL12/CNR1/GPR18/PRKCB/LPAR3/MT1H/CASR/PTPRC/TRPV6/GLP1R/PKHD1/ATP13A4/SLC39A5/XCR1/CCL21/CXCR5/CXCL9/SLC30A8/FFAR1/MS4A1/IAPP/CCL19*	63
BP	GO:0050808	Synapse organization	56/917	419/18,800	6.68E-12	1.24E-09	1.06E-09	*ERBB4/NRCAM/CTTNBP2/SYBU/CEL/NTRK2/CTNND2/AMIGO1/NLGN1/RAB39B/CX3CR1/PAK3/RELN/KIF1A/GABRB3/CNKSR2/ADGRB3/SLC8A3/SLC8A2/CACNB2/NFASC/SDK1/FLRT2/BSN/NRXN1/TNR/PCDHB5/PCLO/NEURL1/LRRTM2/CHRNB2/GHRL/SLITRK1/MUSK/MAPT/SEMA3E/UNC13A/LRRK2/CNTN2/C1QL3/DSCAM/INA/CBLN1/CTNNA2/IGFN1/ITPKA/GABRG2/GPM6A/SEZ6/CBLN4/CAMK2B/CNTN5/GABRA2/LHFPL4/KLK8/LRRTM3*	56
BP	GO:0001508	Action potential	30/917	143/18,800	9.23E-12	1.64E-09	1.41E-09	*NRCAM/NTRK2/P2RX1/KCNJ5/KCNA2/AKAP6/FGF12/CD36/KCNH7/SLC8A2/TACR1/SCN3A/DMD/CACNB2/KCNB1/SLC8A1/SCN4A/KCND3/CHRNB2/ANK2/SCN3B/SCN7A/KCNA5/KCNJ3/KCNMB2/HCN4/CNR1/KCNH6/GJD2/GLRA1*	30
BP	GO:0055074	Calcium ion homeostasis	59/917	468/18,800	2.07E-11	3.55E-09	3.05E-09	*PLA2G1B/KCNK3/CTRC/P2RX1/CX3CR1/NOS1/AKAP6/BNIP3/KL/CD36/RGN/EPO/SLC8A3/SLC8A2/TACR1/DMD/CACNB2/RIC3/SLC8A1/PTGFR/FASLG/GALR1/CYSLTR1/GHRL/ADCYAP1/ATP2B2/ANK2/CCR2/MCOLN3/CCKBR/SPINK1/CCL14/GPR174/SCGN/CXCR6/CACNA1B/CCR5/AVPR1B/KCNA5/CXCL12/CNR1/GPR18/PRKCB/LPAR3/CASR/PTPRC/TRPV6/GLP1R/PKHD1/ATP13A4/XCR1/CCL21/CXCR5/CXCL9/S100A14/FFAR1/MS4A1/IAPP/CCL19*	59
BP	GO:0006836	Neurotransmitter transport	36/917	207/18,800	2.58E-11	4.27E-09	3.67E-09	*SLC1A2/CHRNB3/P2RX1/NLGN1/NOS1/SV2B/SLC6A16/ADCY1/CACNB2/SLC5A7/SLC6A4/NRXN1/PCLO/GRIK5/SYT4/SLC18A3/UNC13A/CADPS/SLC6A15/LRRK2/SLC29A4/SYP/SYT9/SLC18A2/CHAT/CNR1/RIMS2/SNAP25/RIMS1/PRKCB/CPLX2/SYT5/SLC6A17/GABRA2/GRM4/SYT8*	36

(*Continued*)

 © 2023. The Author(s).

Global Medical Genetics © 2023. The Author(s).

Supplementary Table S1 (*Continued*)

Ontology	ID	Description	GeneRatio	BgRatio	p-Value	*p*.adjust	q-Value	geneID	Count
BP	GO:0050708	Regulation of protein secretion	40/917	252/18,800	3.88E-11	6.13E-09	5.27E-09	*MYRIP/BRSK2/PLA2G1B/CELA2A/GPLD1/ABAT/CARTPT/KCNB1/GHRL/SYT4/GPR27/SFRP1/NKX6-1/KCNJ11/PCSK1/STXBP5L/NEUROD1/SYT9/NR0B2/KCNA5/CNR1/SNAP25/CASR/RFX6/ZBED6/ABCC8/FGA/EPHA5/CFTR/GCK/NR1H4/IGF1/FGB/CHGA/G6PC2/SLC30A8/FFAR1/UCN3/BLK/FGG*	40
BP	GO:1903532	Positive regulation of secretion by cell	42/917	274/18,800	3.97E-11	6.13E-09	5.27E-09	*MYRIP/PLA2G1B/NLGN1/GPLD1/VIP/ABAT/CARTPT/TACR1/CACNB2/KCNB1/SLC6A4/SCAMP5/GALR1/CHRNB2/GHRL/ADCYAP1/SYT4/GPR27/CADPS/NKX6-1/SPINK1/PCSK1/SOX11/SYT9/NR0B2/AVPR1B/CXCL12/CNR1/CASR/RFX6/FGA/CFTR/GCK/NR1H4/IGF1/FGB/CDK5R2/SLC30A8/FFAR1/UCN3/BLK/FGG*	42
BP	GO:0006874	Cellular calcium ion homeostasis	57/917	456/18,800	6.42E-11	9.59E-09	8.24E-09	*PLA2G1B/KCNK3/CTRC/P2RX1/CX3CR1/NOS1/AKAP6/BNIP3/CD36/RGN/EPO/SLC8A3/SLC8A2/TACR1/DMD/CACNB2/RIC3/SLC8A1/PTGFR/FASLG/GALR1/CYSLTR1/GHRL/ADCYAP1/ATP2B2/ANK2/CCR2/MCOLN3/CCKBR/SPINK1/CCL14/GPR174/SCGN/CXCR6/CACNA1B/CCR5/AVPR1B/KCNA5/CXCL12/CNR1/GPR18/PRKCB/LPAR3/CASR/PTPRC/TRPV6/GLP1R/PKHD1/ATP13A4/XCR1/CCL21/CXCR5/CXCL9/FFAR1/MS4A1/IAPP/CCL19*	57
BP	GO:0008037	Cell recognition	37/917	228/18,800	1.10E-10	1.59E-08	1.36E-08	*NRCAM/AMIGO1/CD36/DSCAML1/CLGN/FCN2/CNTN2/DSCAM/CNR1/IGHV3-64/SPACA3/C4BPA/IGHV6-1/IGHV2-5/IGHV3-74/IGHV4-4/IGHV3-20/IGHV3-7/IGHV3-15/CCL21/IGHV1-45/IGHV3-72/IGHV3-48/PAEP/IGHV2-26/IGHV3-49/IGHV4-28/IGHV5-51/CRP/IGHV1-69/IGHV4-39/IGHV4-59/IGHA2/IGHV3-21/IGHV3-13/CCL19/IGHV1-18*	37
BP	GO:0050853	B-cell receptor signaling pathway	27/917	131/18,800	1.54E-10	2.14E-08	1.84E-08	*VAV3/PRKCB/IGHV3-64/PTPRC/IGHV6-1/IGHV2-5/IGHV3-74/IGHV4-4/IGHV3-20/IGHV3-7/IGHV3-15/IGHV1-45/IGHV3-72/IGHV3-48/IGHV2-26/IGHV3-49/IGHV4-28/MS4A1/IGHV5-51/IGHV1-69/IGHV4-39/IGHV4-59/BLK/IGHA2/IGHV3-21/IGHV3-13/IGHV1-18*	27
BP	GO:0050804	Modulation of chemical synaptic transmission	54/917	429/18,800	1.59E-10	2.14E-08	1.84E-08	*PRKAR2B/CEL/CHRNB3/NTRK2/P2RX1/NLGN1/CX3CR1/RELN/AKAP7/ADCY1/SLC8A3/SLC8A2/TACR1/CACNB2/KCNB1/SLC6A4/NRXN1/TNR/NPY5R/PHF24/NEURL1/LRRTM2/KIT/CHRNB2/CELF4/SLC4A10/GHRL/GRIK5/FAM107A/SYT4/MAPT/*	54

Supplementary Table S1 (*Continued*)

Ontology	ID	Description	GeneRatio	BgRatio	p-Value	*p*.adjust	q-Value	geneID	Count
								UNC13A/CCR2/LRRK2/CNTN2/SHISA9/SYP/SCGN/MAPK8IP2/ELAVL4/CBLN1/CACNA1B/CACNG5/CNR1/RIMS2/SNAP25/RIMS1/PRKCB/ITPKA/CPLX2/ADIPOQ/CAMK2B/GRM4/SCTR	
BP	GO:0051480	Regulation of cytosolic calcium ion concentration	48/917	356/18,800	1.62E-10	2.14E-08	1.84E-08	*PLA2G1B/KCNK3/P2RX1/CX3CR1/NOS1/AKAP6/CD36/EPO/SLC8A3/SLC8A2/TACR1/DMD/CACNB2/RIC3/SLC8A1/PTGFR/FASLG/GALR1/CYSLTR1/GHRL/ADCYAP1/ATP2B2/ANK2/CCR2/MCOLN3/CCKBR/SPINK1/GPR174/SCGN/CXCR6/CACNA1B/CCR5/AVPR1B/KCNA5/CNR1/GPR18/LPAR3/PTPRC/TRPV6/GLP1R/XCR1/CCL21/CXCR5/CXCL9/FFAR1/MS4A1/IAPP/CCL19*	48
BP	GO:0099177	Regulation of trans-synaptic signaling	54/917	430/18,800	1.74E-10	2.23E-08	1.92E-08	*PRKAR2B/CEL/CHRNB3/NTRK2/P2RX1/NLGN1/CX3CR1/RELN/AKAP7/ADCY1/SLC8A3/SLC8A2/TACR1/CACNB2/KCNB1/SLC6A4/NRXN1/TNR/NPY5R/PHF24/NEURL1/LRRTM2/KIT/CHRNB2/CELF4/SLC4A10/GHRL/GRIK5/FAM107A/SYT4/MAPT/UNC13A/CCR2/LRRK2/CNTN2/SHISA9/SYP/SCGN/MAPK8IP2/ELAVL4/CBLN1/CACNA1B/CACNG5/CNR1/RIMS2/SNAP25/RIMS1/PRKCB/ITPKA/CPLX2/ADIPOQ/CAMK2B/GRM4/SCTR*	54
BP	GO:0051047	Positive regulation of secretion	43/917	300/18,800	2.11E-10	2.64E-08	2.27E-08	*MYRIP/PLA2G1B/NLGN1/GPLD1/VIP/ABAT/CARTPT/TACR1/CACNB2/KCNB1/SLC6A4/SCAMP5/GALR1/CHRNB2/GHRL/ADCYAP1/SYT4/GPR27/CADPS/NKX6-1/SPINK1/PCSK1/SOX11/SYT9/NR0B2/AVPR1B/CXCL12/CNR1/CASR/RFX6/FGA/CYP4A11/CFTR/GCK/NR1H4/IGF1/FGB/CDK5R2/SLC30A8/FFAR1/UCN3/BLK/FGG*	43
BP	GO:0035637	Multicellular organismal signaling	30/917	164/18,800	3.30E-10	4.02E-08	3.46E-08	*NRCAM/NTRK2/P2RX1/KCNJ5/KCNA2/FGF12/CARTPT/SLC8A3/SLC8A2/SCN3A/CACNB2/NFASC/SLC8A1/TNR/SCN4A/KCND3/GHRL/ATP2B2/ABCC9/ANK2/SCN3B/SCN7A/CACNG5/ATP1A3/KCNA5/KCNJ3/KCNMB2/HCN4/KCNH6/GLRA1*	30
BP	GO:0007204	Positive regulation of cytosolic calcium ion concentration	44/917	325/18,800	8.29E-10	9.84E-08	8.46E-08	*PLA2G1B/P2RX1/CX3CR1/NOS1/AKAP6/CD36/EPO/SLC8A3/SLC8A2/TACR1/DMD/CACNB2/RIC3/SLC8A1/PTGFR/FASLG/GALR1/CYSLTR1/GHRL/ADCYAP1/ANK2/CCR2/MCOLN3/CCKBR/SPINK1/GPR174/CXCR6/CACNA1B/CCR5/AVPR1B/CNR1/GPR18/LPAR3/PTPRC/TRPV6/GLP1R/XCR1/CCL21/CXCR5/CXCL9/FFAR1/MS4A1/IAPP/CCL19*	44

(*Continued*)

 © 2023. The Author(s).

Supplementary Table S1 (*Continued*)

Ontology	ID	Description	GeneRatio	BgRatio	p-Value	*p*.adjust	q-Value	geneID	Count
BP	GO:0034765	Regulation of ion transmembrane transport	56/917	476/18,800	9.51E-10	1.10E-07	9.46E-08	*P2RX1/PM20D1/ABCB1/KCNJ5/AMIGO1/NLGN1/KCNA2/KCNAB1/NOS1/AKAP6/KCNJ16/RELN/FGF12/WNK2/RGN/DPP10/CHRM3/EPO/KCNH7/FXYD2/SCN3A/DMD/CACNB2/KCNB1/SLC8A1/PIRT/KCNA3/SCN4A/KCND3/CLTRN/PDZK1/CRHBP/ANK2/KCNV1/CCR2/SHISA9/KCNJ11/CACNA2D2/SCN3B/SCN7A/MAPK8IP2/MIR210/CACNA1B/CACNG5/DPP6/KCNJ3/KCNB2/KCNG3/ABCC8/CFTR/KCNJ6/CLIC3/MRLN/KCNK16/CXCL9/PLP1*	56
BP	GO:0001505	Regulation of neurotransmitter levels	34/917	213/18,800	9.77E-10	1.10E-07	9.49E-08	*SLC1A2/CHRNB3/P2RX1/NLGN1/NOS1/ABAT/ADCY1/CACNB2/SLC5A7/SLC6A4/NRXN1/PAH/PCLO/GRIK5/SYT4/UNC13A/CADPS/LRRK2/SLC29A4/SYP/SYT9/SLC18A2/CHAT/CNR1/RIMS2/SNAP25/RIMS1/PRKCB/CPLX2/GAD2/SYT5/GABRA2/GRM4/SYT8*	34
BP	GO:0090277	Positive regulation of peptide hormone secretion	21/917	91/18,800	1.94E-09	2.13E-07	1.84E-07	*MYRIP/GPLD1/ABAT/GHRL/ADCYAP1/GPR27/NKX6-1/SPINK1/NR0B2/CASR/RFX6/FGA/CFTR/GCK/NR1H4/FGB/SLC30A8/FFAR1/UCN3/BLK/FGG*	21
BP	GO:0006911	Phagocytosis, engulfment	25/917	128/18,800	2.40E-09	2.58E-07	2.22E-07	*CD36/XKR4/XKR7/IGHV3-64/IGHV6-1/IGHV2-5/IGHV3-74/IGHV4-4/IGHV3-20/IGHV3-7/IGHV3-15/IGHV1-45/IGHV3-72/IGHV3-48/IGHV2-26/IGHV3-49/IGHV4-28/IGHV5-51/IGHV1-69/IGHV4-39/IGHV4-59/IGHA2/IGHV3-21/IGHV3-13/IGHV1-18*	25
BP	GO:0002253	Activation of immune response	48/917	386/18,800	2.53E-09	2.62E-07	2.25E-07	*C5/GPLD1/CLU/PAK3/FYB2/C6/VAV3/C7/PVRIG/THEMIS/FCN2/C8A/LAX1/CD8A/PRKCB/IGHV3-64/PYHIN1/PTPRC/C4BPA/IGHV6-1/IGHV2-5/IGHV3-74/SH2D1A/TRAT1/IGHV4-4/PLPP4/IGHV3-20/KRT1/IGHV3-7/IGHV3-15/IGHV1-45/IGHV3-72/IGHV3-48/APCS/IGHV2-26/IGHV3-49/IGHV4-28/MS4A1/IGHV5-51/CRP/IGHV1-69/IGHV4-39/IGHV4-59/BLK/IGHA2/IGHV3-21/IGHV3-13/IGHV1-18*	48
BP	GO:0006813	Potassium ion transport	36/917	243/18,800	2.55E-09	2.62E-07	2.25E-07	*KCNK3/KCNJ5/AMIGO1/KCNA2/KCNAB1/NOS1/AKAP6/KCNJ16/WNK2/VIP/DPP10/KCNH7/FXYD2/KCNB1/KCNA3/KCND3/ABCC9/ANK2/KCNV1/KCNJ11/HPN/ATP1A3/DPP6/KCNA5/KCNJ3/KCNMB2/HCN4/SNAP25/KCNB2/KCNG3/ATP4A/ABCC8/KCNH6/GCK/KCNJ6/KCNK16*	36
BP	GO:0002793	Positive regulation of peptide secretion	21/917	93/18,800	2.95E-09	2.97E-07	2.56E-07	*MYRIP/GPLD1/ABAT/GHRL/ADCYAP1/GPR27/NKX6-1/SPINK1/NR0B2/CASR/RFX6/FGA/CFTR/GCK/NR1H4/FGB/SLC30A8/FFAR1/UCN3/BLK/FGG*	21

 © 2023. The Author(s).

Supplementary Table S1 (*Continued*)

Ontology	ID	Description	GeneRatio	BgRatio	p-Value	*p*.adjust	q-Value	geneID	Count
BP	GO:0019932	Second messenger-mediated signaling	41/917	303/18,800	3.18E-09	3.13E-07	2.69E-07	*BHLHA15/PEX5L/CX3CR1/C10orf71/NOS1/AKAP6/NR5A2/CD36/RGN/ADCY1/CHRM3/SLC8A2/P2RY12/DMD/KSR2/PDE3A/HOMER2/SLC8A1/PTGFR/SELP/PDE3B/ADCYAP1/MAPT/ANK2/CCR2/LRRK2/SPINK1/NEUROD1/CXCR6/SELE/CCR5/AGTR2/PTPRC/GLP1R/XCR1/EPHA5/TRAT1/IGF1/RUNDC3A/CXCR5/SCTR*	41
BP	GO:0046887	Positive regulation of hormone secretion	24/917	122/18,800	4.30E-09	4.15E-07	3.57E-07	*MYRIP/GPLD1/ABAT/TACR1/GALR1/GHRL/ADCYAP1/GPR27/NKX6-1/SPINK1/SOX11/NR0B2/CASR/RFX6/FGA/CFTR/GCK/NR1H4/FGB/SLC30A8/FFAR1/UCN3/BLK/FGG*	24
BP	GO:0099504	Synaptic vesicle cycle	30/917	183/18,800	4.97E-09	4.70E-07	4.04E-07	*BRSK2/CEL/CHRNB3/P2RX1/NLGN1/DNAJC6/SH3GL3/ADCY1/CACNB2/BSN/PACSIN1/PCLO/GRIK5/SYT4/UNC13A/CADPS/LRRK2/SYP/SYT9/SLC18A2/CNR1/RIMS2/SNAP25/RIMS1/PRKCB/SNAP91/SH3GL2/CPLX2/SYT5/SYT8*	30
BP	GO:0099003	Vesicle-mediated transport in synapse	31/917	197/18,800	7.59E-09	6.92E-07	5.95E-07	*BRSK2/CEL/CHRNB3/P2RX1/NLGN1/DNAJC6/SH3GL3/ADCY1/CACNB2/BSN/PACSIN1/PCLO/GRIK5/SYT4/UNC13A/CADPS/LRRK2/SYP/SYT9/SLC18A2/CNR1/RIMS2/SNAP25/RIMS1/PRKCB/SNAP91/SH3GL2/CPLX2/SYT5/CALY/SYT8*	31
BP	GO:0071805	Potassium ion transmembrane transport	33/917	219/18,800	7.62E-09	6.92E-07	5.95E-07	*KCNK3/KCNJ5/AMIGO1/KCNA2/KCNAB1/AKAP6/KCNJ16/WNK2/DPP10/KCNH7/FXYD2/KCNB1/KCNA3/KCND3/ABCC9/ANK2/KCNV1/KCNJ11/HPN/ATP1A3/DPP6/KCNA5/KCNJ3/KCNMB2/HCN4/SNAP25/KCNB2/KCNG3/ATP4A/ABCC8/KCNH6/KCNJ6/KCNK16*	33
BP	GO:0099024	Plasma membrane invagination	25/917	137/18,800	1.03E-08	9.16E-07	7.87E-07	*CD36/XKR4/XKR7/IGHV3-64/IGHV6-1/IGHV2-5/IGHV3-74/IGHV4-4/IGHV3-20/IGHV3-7/IGHV3-15/IGHV1-45/IGHV3-72/IGHV3-48/IGHV2-26/IGHV3-49/IGHV4-28/IGHV5-51/IGHV1-69/IGHV4-39/IGHV4-59/IGHA2/IGHV3-21/IGHV3-13/IGHV1-18*	25
BP	GO:0015844	Monoamine transport	19/917	83/18,800	1.31E-08	1.14E-06	9.83E-07	*P2RX1/SYT6/KCNA2/NOS1/VIP/ABAT/CARTPT/KCNB1/SLC6A4/CHRNB2/SYT4/SLC29A4/SYT9/SLC18A2/CXCL12/CNR1/SYT5/CHGA/SYT8*	19
BP	GO:0007416	Synapse assembly	29/917	180/18,800	1.34E-08	1.15E-06	9.87E-07	*ERBB4/NRCAM/CEL/NTRK2/AMIGO1/NLGN1/GABRB3/ADGRB3/SDK1/FLRT2/BSN/NRXN1/PCDHB5/PCLO/LRRTM2/CHRNB2/GHRL/SLITRK1/MUSK/DSCAM/CBLN1/IGFN1/GABRG2/GPM6A/CBLN4/CNTN5/GABRA2/LHFPL4/LRRTM3*	29

(*Continued*)

 © 2023. The Author(s).

 © 2023. The Author(s).

Supplementary Table S1 (*Continued*)

Ontology	ID	Description	GeneRatio	BgRatio	p-Value	*p*.adjust	q-Value	geneID	Count
BP	GO:0017157	Regulation of exocytosis	31/917	204/18,800	1.77E-08	1.49E-06	1.28E-06	*CHRNB3/P2RX1/SYT6/NLGN1/RAB3C/ADCY1/CACNB2/KCNB1/SCAMP5/PCLO/SYT4/CADPS/CCR2/LRRK2/STXBP5L/SYP/SYT9/CNR1/RIMS2/LGI3/PRKCB/TRPV6/FGA/CPLX2/CFTR/SYT5/FGB/CDK5R2/SYT8/BLK/FGG*	31
BP	GO:0060078	Regulation of postsynaptic membrane potential	24/917	131/18,800	1.87E-08	1.54E-06	1.33E-06	*CHRNB3/P2RX1/NLGN1/RELN/ABAT/SLC8A3/SLC8A2/NRXN1/CHRNB2/CELF4/GHRL/GRIK5/LRRK2/RGS7BP/MAPK8IP2/GLRA3/CBLN1/RIMS2/RIMS1/GABRA4/GABRG2/SEZ6/GLRA1/GABRA2*	24
BP	GO:0016064	Immunoglobulin mediated immune response	32/917	216/18,800	1.95E-08	1.58E-06	1.36E-06	*C5/CLU/C6/C7/C8A/IGHV3-64/PTPRC/C4BPA/TLR8/IGHV6-1/IGHV2-5/IGHV3-74/IGHV4-4/IGHV3-20/IGHV3-7/IGHV3-15/IGHV1-45/IGHV3-72/IGHV3-48/APCS/IGHV2-26/IGHV3-49/IGHV4-28/IGHV5-51/CRP/IGHV1-69/IGHV4-39/IGHV4-59/IGHA2/IGHV3-21/IGHV3-13/IGHV1-18*	32
BP	GO:0019724	B cell-mediated immunity	32/917	219/18,800	2.73E-08	2.18E-06	1.87E-06	*C5/CLU/C6/C7/C8A/IGHV3-64/PTPRC/C4BPA/TLR8/IGHV6-1/IGHV2-5/IGHV3-74/IGHV4-4/IGHV3-20/IGHV3-7/IGHV3-15/IGHV1-45/IGHV3-72/IGHV3-48/APCS/IGHV2-26/IGHV3-49/IGHV4-28/IGHV5-51/CRP/IGHV1-69/IGHV4-39/IGHV4-59/IGHA2/IGHV3-21/IGHV3-13/IGHV1-18*	32
BP	GO:0010324	Membrane invagination	25/917	144/18,800	2.91E-08	2.28E-06	1.96E-06	*CD36/XKR4/XKR7/IGHV3-64/IGHV6-1/IGHV2-5/IGHV3-74/IGHV4-4/IGHV3-20/IGHV3-7/IGHV3-15/IGHV1-45/IGHV3-72/IGHV3-48/IGHV2-26/IGHV3-49/IGHV4-28/IGHV5-51/IGHV1-69/IGHV4-39/IGHV4-59/IGHA2/IGHV3-21/IGHV3-13/IGHV1-18*	25
BP	GO:0050851	Antigen receptor-mediated signaling pathway	34/917	244/18,800	3.37E-08	2.60E-06	2.24E-06	*FYB2/VAV3/PVRIG/THEMIS/LAX1/CD8A/PRKCB/IGHV3-64/PTPRC/IGHV6-1/IGHV2-5/IGHV3-74/SH2D1A/TRAT1/IGHV4-4/IGHV3-20/IGHV3-7/IGHV3-15/IGHV1-45/IGHV3-72/IGHV3-48/IGHV2-26/IGHV3-49/IGHV4-28/MS4A1/IGHV5-51/IGHV1-69/IGHV4-39/IGHV4-59/BLK/IGHA2/IGHV3-21/IGHV3-13/IGHV1-18*	34
BP	GO:0016079	Synaptic vesicle exocytosis	21/917	107/18,800	4.14E-08	3.14E-06	2.70E-06	*CHRNB3/P2RX1/NLGN1/ADCY1/CACNB2/PCLO/GRIK5/SYT4/UNC13A/CADPS/LRRK2/SYP/SYT9/CNR1/RIMS2/SNAP25/RIMS1/PRKCB/CPLX2/SYT5/SYT8*	21
BP	GO:0007586	Digestion	24/917	137/18,800	4.59E-08	3.43E-06	2.95E-06	*AMY2A/CYP39A1/CEL/PNLIP/CLPS/PNLIPRP2/PRSS1/CD36/CTRB2/CTRB1/CHRM3/TACR1/PRSS2/GHRL/CCKBR/SPINK1/NEUROD1/FGF10/PRSS3/ARX/CLPSL1/UCN3/SST/MUC6*	24

Supplementary Table S1 (*Continued*)

Ontology	ID	Description	GeneRatio	BgRatio	p-Value	*p*.adjust	q-Value	geneID	Count
BP	GO:1903522	Regulation of blood circulation	35/917	260/18,800	5.14E-08	3.73E-06	3.20E-06	*SPX/MYH7/P2RX1/KCNJ5/NOS1/MYH6/CHRM3/SLC8A3/SLC8A2/TACR1/DMD/CACNB2/SLC8A1/KCND3/CYSLTR1/GHRL/ATP2B2/ABCC9/ANK2/SCN3B/ATP1A3/AVPR1B/KCNA5/KCNJ3/CHRM2/KCNMB2/HCN4/AGTR2/CASR/KCNH6/GLP1R/FGA/FGB/CHGA/FGG*	35
BP	GO:0098659	Inorganic cation import across plasma membrane	22/917	118/18,800	5.23E-08	3.73E-06	3.20E-06	*SCNN1B/P2RX1/KCNJ5/KCNJ16/WNK2/SLC8A3/SLC8A2/FXYD2/SLC8A1/SCNN1G/ABCC9/KCNJ11/CNGA3/MIR210/CACNA1B/ATP1A3/KCNJ3/HCN4/ATP4A/TRPV6/SLC39A5/KCNJ6*	22
BP	GO:0099587	Inorganic ion import across plasma membrane	22/917	118/18,800	5.23E-08	3.73E-06	3.20E-06	*SCNN1B/P2RX1/KCNJ5/KCNJ16/WNK2/SLC8A3/SLC8A2/FXYD2/SLC8A1/SCNN1G/ABCC9/KCNJ11/CNGA3/MIR210/CACNA1B/ATP1A3/KCNJ3/HCN4/ATP4A/TRPV6/SLC39A5/KCNJ6*	22
BP	GO:0098657	Import into cell	33/917	238/18,800	6.04E-08	4.24E-06	3.64E-06	*SPX/SLC1A2/SCNN1B/P2RX1/KCNJ5/SLC7A2/NOS1/KCNJ16/WNK2/CD36/SLC8A3/SLC8A2/FXYD2/SLC6A4/SLC8A1/CLTRN/SCNN1G/ABCC9/SLC29A4/KCNJ11/CNGA3/SLC18A2/ITGA4/MIR210/CACNA1B/ATP1A3/KCNJ3/HCN4/SNAP25/ATP4A/TRPV6/SLC39A5/KCNJ6*	33
BP	GO:0099565	Chemical synaptic transmission, postsynaptic	20/917	101/18,800	7.41E-08	5.12E-06	4.40E-06	*CHRNB3/P2RX1/NLGN1/RELN/ABAT/SLC8A3/SLC8A2/NRXN1/CHRNB2/CELF4/GHRL/GRIK5/LRRK2/MAPK8IP2/GLRA3/CBLN1/RIMS2/RIMS1/SEZ6/GLRA1*	20
BP	GO:0050871	Positive regulation of B cell activation	25/917	152/18,800	8.78E-08	5.97E-06	5.13E-06	*VAV3/CHRNB2/IGHV3-64/PTPRC/IGHV6-1/IGHV2-5/IGHV3-74/IGHV4-4/IGHV3-20/IGHV3-7/IGHV3-15/IGHV1-45/IGHV3-72/IGHV3-48/IGHV2-26/IGHV3-49/IGHV4-28/IGHV5-51/IGHV1-69/IGHV4-39/IGHV4-59/IGHA2/IGHV3-21/IGHV3-13/IGHV1-18*	25
BP	GO:0002768	Immune response-regulating cell surface receptor signaling pathway	40/917	328/18,800	9.25E-08	6.21E-06	5.34E-06	*GPLD1/PAK3/FYB2/VAV3/PVRIG/THEMIS/KIT/LAX1/CD8A/PRKCB/IGHV3-64/PTPRC/MS4A2/IGHV6-1/IGHV2-5/IGHV3-74/BTLA/SH2D1A/TRAT1/IGHV4-4/PLPP4/IGHV3-20/IGHV3-7/IGHV3-15/IGHV1-45/IGHV3-72/IGHV3-48/IGHV2-26/IGHV3-49/IGHV4-28/MS4A1/IGHV5-51/IGHV1-69/IGHV4-39/IGHV4-59/BLK/IGHA2/IGHV3-21/IGHV3-13/IGHV1-18*	40
BP	GO:0032024	Positive regulation of insulin secretion	16/917	67/18,800	9.72E-08	6.43E-06	5.53E-06	*MYRIP/GPLD1/ABAT/GHRL/GPR27/NKX6-1/NR0B2/CASR/RFX6/CFTR/GCK/NR1H4/SLC30A8/FFAR1/UCN3/BLK*	16

(*Continued*)

 © 2023. The Author(s).

Supplementary Table S1 (*Continued*)

Ontology	ID	Description	GeneRatio	BgRatio	p-Value	*p*.adjust	q-Value	geneID	Count
BP	GO:0007188	Adenylate cyclase-modulating G protein-coupled receptor signaling pathway	33/917	244/18,800	1.10E-07	7.19E-06	6.18E-06	*GPHA2/VIPR2/MTNR1A/VIP/ADCY1/CHRM3/ADGRB3/GPR37/P2RY12/PTGFR/GALR1/TSHR/GNAZ/ADCYAP1/GNAO1/ADGRG2/APLP1/SSTR2/AVPR1B/CHRM2/CNR1/RIMS2/NPY1R/GPR119/LPAR3/CASR/GLP1R/CHGA/CXCL9/UCN3/GRM4/SCTR/IAPP*	33
BP	GO:0006887	Exocytosis	42/917	357/18,800	1.22E-07	7.84E-06	6.74E-06	*BRSK2/CHRNB3/P2RX1/SYT6/SYCN/NLGN1/RAB3C/ADCY1/CACNB2/KCNB1/SCAMP5/PCLO/KIT/CLTRN/GRIK5/SYT4/CRHBP/UNC13A/CADPS/CCR2/LRRK2/STXBP5L/SYP/SYT9/CNR1/RIMS2/SNAP25/LGI3/RIMS1/PRKCB/TRPV6/FGA/CPLX2/CFTR/SYT5/FGB/CHGA/TRARG1/CDK5R2/SYT8/BLK/FGG*	42
BP	GO:0045055	Regulated exocytosis	32/917	234/18,800	1.32E-07	8.29E-06	7.13E-06	*CHRNB3/P2RX1/SYT6/NLGN1/ADCY1/CACNB2/KCNB1/SCAMP5/PCLO/KIT/CLTRN/GRIK5/SYT4/CRHBP/UNC13A/CADPS/CCR2/LRRK2/SYP/SYT9/CNR1/RIMS2/SNAP25/RIMS1/PRKCB/TRPV6/CPLX2/SYT5/CHGA/CDK5R2/SYT8/BLK*	32
BP	GO:0060079	Excitatory postsynaptic potential	19/917	95/18,800	1.33E-07	8.29E-06	7.13E-06	*CHRNB3/P2RX1/NLGN1/RELN/SLC8A3/SLC8A2/NRXN1/CHRNB2/CELF4/GHRL/GRIK5/LRRK2/MAPK8IP2/GLRA3/CBLN1/RIMS2/RIMS1/SEZ6/GLRA1*	19
BP	GO:0043270	Positive regulation of ion transport	35/917	273/18,800	1.74E-07	1.07E-05	9.23E-06	*PLA2G1B/P2RX1/ABCB1/AMIGO1/NOS1/AKAP6/RELN/FGF12/WNK2/RGN/ABAT/FXYD2/P2RY12/DMD/CACNB2/SLC6A4/SLC38A3/PIRT/CLTRN/CHRNB2/PDZK1/SYT4/ANK2/CCR2/SCN3B/MIR210/AVPR1B/CXCL12/CASR/ABCC8/CYP4A11/CFTR/CXCL9/PLP1/FFAR1*	35
BP	GO:0006814	Sodium ion transport	33/917	249/18,800	1.79E-07	1.09E-05	9.36E-06	*SCNN1B/NOS1/FGF12/WNK2/SLC6A16/SLC5A9/SLC8A3/SLC8A2/FXYD2/SCN3A/DMD/SLC5A7/SLC6A4/SLC8A1/SLC5A4/SLC38A3/SCN4A/SLC4A4/SLC4A10/SCNN1G/SLC17A4/SLC6A15/SLC17A1/SCN3B/SCN7A/SLC38A11/SLC38A4/ATP1A3/SLC5A8/HCN4/SLC10A2/ATP4A/SLC6A17*	33
BP	GO:0002429	Immune response-activating cell surface receptor signaling pathway	37/917	300/18,800	2.10E-07	1.25E-05	1.07E-05	*GPLD1/PAK3/FYB2/VAV3/PVRIG/THEMIS/LAX1/CD8A/PRKCB/IGHV3-64/PTPRC/IGHV6-1/IGHV2-5/IGHV3-74/SH2D1A/TRAT1/IGHV4-4/PLPP4/IGHV3-20/IGHV3-7/IGHV3-15/IGHV1-45/IGHV3-72/IGHV3-48/IGHV2-26/IGHV3-49/IGHV4-28/MS4A1/IGHV5-51/IGHV1-69/IGHV4-39/IGHV4-59/BLK/IGHA2/IGHV3-21/IGHV3-13/IGHV1-18*	37

 © 2023. The Author(s).

Supplementary Table S1 (*Continued*)

Ontology	ID	Description	GeneRatio	BgRatio	p-Value	*p*.adjust	q-Value	geneID	Count
BP	GO:0002757	Immune response-activating signal transduction	37/917	300/18,800	2.10E-07	1.25E-05	1.07E-05	*GPLD1/PAK3/FYB2/VAV3/PVRIG/THEMIS/LAX1/CD8A/PRKCB/IGHV3-64/PTPRC/IGHV6-1/IGHV2-5/IGHV3-74/SH2D1A/TRAT1/IGHV4-4/PLPP4/IGHV3-20/IGHV3-7/IGHV3-15/IGHV1-45/IGHV3-72/IGHV3-48/IGHV2-26/IGHV3-49/IGHV4-28/MS4A1/IGHV5-51/IGHV1-69/IGHV4-39/IGHV4-59/BLK/IGHA2/IGHV3-21/IGHV3-13/IGHV1-18*	37
BP	GO:0003018	Vascular process in circulatory system	34/917	263/18,800	2.13E-07	1.25E-05	1.07E-05	*SLC1A2/SCNN1B/P2RX1/ABCB1/SLC16A12/SLC7A2/NOS1/CD36/UTS2R/CHRM3/SLC8A2/TACR1/PDE3A/SLC6A4/SLC8A1/SLC38A3/SLCO1C1/C2CD4B/CYSLTR1/SLC4A4/ABCC9/SLC29A4/AVPR1B/KCNA5/KCNMB2/AGTR2/SH3GL2/CASR/ABCC8/FGA/FGB/SLC6A17/CRP/FGG*	34
BP	GO:0050714	Positive regulation of protein secretion	22/917	128/18,800	2.34E-07	1.36E-05	1.17E-05	*MYRIP/PLA2G1B/GPLD1/ABAT/GHRL/GPR27/NKX6-1/PCSK1/NR0B2/CASR/RFX6/FGA/CFTR/GCK/NR1H4/IGF1/FGB/SLC30A8/FFAR1/UCN3/BLK/FGG*	22
BP	GO:0051937	Catecholamine transport	16/917	72/18,800	2.83E-07	1.62E-05	1.39E-05	*SYT6/KCNA2/VIP/ABAT/CARTPT/KCNB1/CHRNB2/SYT4/SLC29A4/SYT9/SLC18A2/CXCL12/CNR1/SYT5/CHGA/SYT8*	16
BP	GO:0098742	Cell–cell adhesion via plasma membrane adhesion molecules	35/917	279/18,800	2.96E-07	1.67E-05	1.44E-05	*UMOD/MMP24/AMIGO1/PCDHA3/NLGN1/PCDHA10/PCDHA4/REG3A/DSCAML1/SDK1/PCDHB1/PCDHB5/SELP/RET/PTPRT/PCDHA6/SLITRK1/CDH22/PCDH9/PCDHA1/CNTN2/DSCAM/CBLN1/PCDHA11/SELE/IGFN1/PCDHA12/PCDHAC1/PCDHGB5/CADM3/CDH10/ADIPOQ/NECTIN4/CDH19/UNC5D*	35
BP	GO:0015837	Amine transport	19/917	100/18,800	3.09E-07	1.72E-05	1.48E-05	*SYT6/KCNA2/VIP/ABAT/CARTPT/KCNB1/SLC38A3/NPY5R/CLTRN/CHRNB2/SYT4/SYT9/SLC18A2/AVPR1B/CXCL12/CNR1/SYT5/CHGA/SYT8*	19
BP	GO:0050433	Regulation of catecholamine secretion	14/917	56/18,800	3.34E-07	1.84E-05	1.58E-05	*SYT6/KCNA2/VIP/ABAT/CARTPT/KCNB1/CHRNB2/SYT4/SYT9/CXCL12/CNR1/SYT5/CHGA/SYT8*	14
BP	GO:0051952	Regulation of amine transport	18/917	92/18,800	3.99E-07	2.17E-05	1.87E-05	*SYT6/KCNA2/VIP/ABAT/CARTPT/KCNB1/SLC38A3/NPY5R/CLTRN/CHRNB2/SYT4/SYT9/AVPR1B/CXCL12/CNR1/SYT5/CHGA/SYT8*	18
BP	GO:0050432	Catecholamine secretion	14/917	57/18,800	4.23E-07	2.27E-05	1.96E-05	*SYT6/KCNA2/VIP/ABAT/CARTPT/KCNB1/CHRNB2/SYT4/SYT9/CXCL12/CNR1/SYT5/CHGA/SYT8*	14

(*Continued*)

 © 2023. The Author(s).

Global Medical Genetics © 2023. The Author(s).

Supplementary Table S1 (*Continued*)

Ontology	ID	Description	GeneRatio	BgRatio	p-Value	*p*.adjust	q-Value	geneID	Count
BP	GO:0015850	Organic hydroxy compound transport	32/917	248/18,800	5.04E-07	2.68E-05	2.30E-05	*CEL/PNLIP/P2RX1/SYT6/KCNA2/CLU/NOS1/CD36/VIP/ABAT/CARTPT/KCNB1/SLC6A4/SLCO1C1/GALR1/CHRNB2/GHRL/SYT4/SLC29A4/SYT9/ABCA8/SLC18A2/NR0B2/SLC5A8/CXCL12/CNR1/SLC10A2/CFTR/SYT5/CHGA/MTTP/SYT8*	32
BP	GO:0009749	Response to glucose	27/917	189/18,800	5.20E-07	2.74E-05	2.35E-05	*BRSK2/GPLD1/CMA1/KCNB1/SLC8A1/AQP4/CLTRN/GHRL/GRIK5/GPR27/NKX6-1/PCSK1/NEUROD1/NR0B2/CASR/RFX6/ZBED6/EPHA5/CFTR/GCK/NKX2-2/NR1H4/ADIPOQ/PTPRN/SLC30A8/PCK1/UCN3*	27
BP	GO:0007269	Neurotransmitter secretion	23/917	145/18,800	5.56E-07	2.86E-05	2.46E-05	*CHRNB3/P2RX1/NLGN1/ADCY1/CACNB2/NRXN1/PCLO/GRIK5/SYT4/UNC13A/CADPS/LRRK2/SYP/SYT9/CNR1/RIMS2/SNAP25/RIMS1/PRKCB/CPLX2/SYT5/GRM4/SYT8*	23
BP	GO:0099643	Signal release from synapse	23/917	145/18,800	5.56E-07	2.86E-05	2.46E-05	*CHRNB3/P2RX1/NLGN1/ADCY1/CACNB2/NRXN1/PCLO/GRIK5/SYT4/UNC13A/CADPS/LRRK2/SYP/SYT9/CNR1/RIMS2/SNAP25/RIMS1/PRKCB/CPLX2/SYT5/GRM4/SYT8*	23
BP	GO:0007156	Homophilic cell adhesion via plasma membrane adhesion molecules	25/917	168/18,800	6.28E-07	3.19E-05	2.75E-05	*AMIGO1/PCDHA3/PCDHA10/PCDHA4/DSCAML1/SDK1/PCDHB1/PCDHB5/RET/PTPRT/PCDHA6/CDH22/PCDH9/PCDHA1/CNTN2/DSCAM/PCDHA11/IGFN1/PCDHA12/PCDHAC1/PCDHGB5/CADM3/CDH10/NECTIN4/CDH19*	25
BP	GO:0009746	Response to hexose	27/917	193/18,800	7.93E-07	3.99E-05	3.43E-05	*BRSK2/GPLD1/CMA1/KCNB1/SLC8A1/AQP4/CLTRN/GHRL/GRIK5/GPR27/NKX6-1/PCSK1/NEUROD1/NR0B2/CASR/RFX6/ZBED6/EPHA5/CFTR/GCK/NKX2-2/NR1H4/ADIPOQ/PTPRN/SLC30A8/PCK1/UCN3*	27
BP	GO:0002377	Immunoglobulin production	29/917	218/18,800	9.09E-07	4.52E-05	3.89E-05	*IGKV2-28/IGKV2D-28/PTPRC/IGLV5-37/IGLV7-46/IGLV7-43/IGKV1D-39/IGKV1-12/IGKV1-39/IGKV5-2/IGLV2-8/IGKV1D-13/IGLV2-18/IGKV1-17/IGKV2-24/IGKV1-6/IGLV4-60/IGLV6-57/IGKV1-16/IGLV4-69/IGLV10-54/IGLV3-27/IGLV5-45/IGKV4-1/IGLV3-19/IGKV3D-20/IGKV1-27/IGKV1-9/IGLV8-61*	29
BP	GO:0007218	Neuropeptide signaling pathway	19/917	108/18,800	1.07E-06	5.25E-05	4.51E-05	*GRPR/SORCS1/UTS2R/CARTPT/GPR37/NPY5R/GALR1/CYSLTR1/ADCYAP1/PRLHR/GLRA3/SSTR2/CPE/NPY1R/SSTR3/SCG5/NPY/GLRA1/PPY*	19
BP	GO:0048167	Regulation of synaptic plasticity	27/917	198/18,800	1.32E-06	6.43E-05	5.53E-05	*NTRK2/CX3CR1/RELN/ADCY1/SLC8A3/SLC8A2/KCNB1/TNR/NEURL1/LRRTM2/KIT/SLC4A10/FAM107A/SYT4/MAPT/CNTN2/SHISA9/SYP/SCGN/CBLN1/RIMS2/SNAP25/RIMS1/ITPKA/CPLX2/CAMK2B/SCTR*	27

Supplementary Table S1 (*Continued*)

Ontology	ID	Description	GeneRatio	BgRatio	p-Value	*p*.adjust	q-Value	geneID	Count
BP	GO:0098739	Import across plasma membrane	26/917	188/18,800	1.58E-06	7.59E-05	6.53E-05	*SLC1A2/SCNN1B/P2RX1/KCNJ5/SLC7A2/KCNJ16/WNK2/CD36/SLC8A3/SLC8A2/FXYD2/SLC8A1/CLTRN/SCNN1G/ABCC9/KCNJ11/CNGA3/MIR210/CACNA1B/ATP1A3/KCNJ3/HCN4/ATP4A/TRPV6/SLC39A5/KCNJ6*	26
BP	GO:0034284	Response to monosaccharide	27/917	200/18,800	1.61E-06	7.59E-05	6.53E-05	*BRSK2/GPLD1/CMA1/KCNB1/SLC8A1/AQP4/CLTRN/GHRL/GRIK5/GPR27/NKX6-1/PCSK1/NEUROD1/NR0B2/CASR/RFX6/ZBED6/EPHA5/CFTR/GCK/NKX2-2/NR1H4/ADIPOQ/PTPRN/SLC30A8/PCK1/UCN3*	27
BP	GO:0050864	Regulation of B cell activation	27/917	200/18,800	1.61E-06	7.59E-05	6.53E-05	*VAV3/CHRNB2/SFRP1/IGHV3-64/PTPRC/IGHV6-1/IGHV2-5/IGHV3-74/IGHV4-4/IGHV3-20/IGHV3-7/IGHV3-15/IGHV1-45/IGHV3-72/IGHV3-48/IGHV2-26/IGHV3-49/IGHV4-28/IGHV5-51/IGHV1-69/IGHV4-39/IGHV4-59/BLK/IGHA2/IGHV3-21/IGHV3-13/IGHV1-18*	27
BP	GO:0035725	Sodium ion transmembrane transport	25/917	177/18,800	1.68E-06	7.85E-05	6.75E-05	*SCNN1B/NOS1/FGF12/WNK2/SLC6A16/SLC8A3/SLC8A2/FXYD2/SCN3A/DMD/SLC6A4/SLC8A1/SCN4A/SLC4A4/SLC4A10/SCNN1G/SLC17A4/SLC6A15/SLC17A1/SCN3B/SCN7A/ATP1A3/HCN4/ATP4A/SLC6A17*	25
BP	GO:0003012	Muscle system process	46/917	449/18,800	1.70E-06	7.85E-05	6.75E-05	*SPX/MYH7/SCNN1B/SORBS2/P2RX1/KCNJ5/DTNA/MYLK2/NOS1/AKAP6/GATM/FGF12/MYH6/KLF15/ABAT/GAMT/CHRM3/SLC8A3/TACR1/DMD/CACNB2/SLC8A1/TMOD1/SCN4A/KCND3/KIT/CHRNB2/GHRL/GNAO1/ANK2/SCN3B/SCN7A/KLHL41/SSTR2/MYBPC2/KCNA5/KCNJ3/CHRM2/HCN4/KCNB2/MYOC/ABCC8/IGF1/CAMK2B/CHGA/GLRA1*	46
BP	GO:0019226	Transmission of nerve impulse	15/917	73/18,800	1.92E-06	8.75E-05	7.52E-05	*NRCAM/NTRK2/P2RX1/KCNA2/FGF12/CARTPT/SCN3A/NFASC/TNR/SCN4A/GHRL/SCN7A/CACNG5/KCNMB2/GLRA1*	15
BP	GO:1903035	Negative regulation of response to wounding	17/917	92/18,800	1.93E-06	8.75E-05	7.52E-05	*F11/RTN4RL1/ABAT/KIAA0319/MMRN1/KLKB1/TNR/VTN/AJAP1/APOH/ABCC8/FGA/FGB/KRT1/APCS/KLK8/FGG*	17
BP	GO:0019722	Calcium-mediated signaling	27/917	202/18,800	1.95E-06	8.78E-05	7.55E-05	*BHLHA15/CX3CR1/C10orf71/AKAP6/NR5A2/RGN/CHRM3/SLC8A2/P2RY12/DMD/KSR2/HOMER2/SLC8A1/PTGFR/SELP/MAPT/ANK2/CCR2/LRRK2/CXCR6/SELE/CCR5/PTPRC/XCR1/TRAT1/IGF1/CXCR5*	27

(*Continued*)

 © 2023. The Author(s).

Supplementary Table S1 (*Continued*)

Ontology	ID	Description	GeneRatio	BgRatio	p-Value	*p*.adjust	q-Value	geneID	Count
BP	GO:0006816	Calcium ion transport	44/917	424/18,800	2.03E-06	9.02E-05	7.76E-05	*BHLHA15/PLA2G1B/P2RX1/NOS1/AKAP6/TRPM6/RGN/EPO/SLC8A3/SLC8A2/DMD/CACNB2/HOMER2/SLC8A1/FASLG/CYSLTR1/CHRNB2/EGF/ATP2B2/ANK2/MCOLN3/CACNA2D2/SPINK1/TRPM3/CACNA1B/CACNG5/CCR5/CXCL12/PRKCB/CASR/PTPRC/GPM6A/TRPV6/XCR1/GCK/CAMK2B/PLPP4/CCL21/MRLN/CXCL9/PLP1/FFAR1/MS4A1/CCL19*	44
BP	GO:0017156	Calcium ion-regulated exocytosis	14/917	65/18,800	2.33E-06	0.000103	8.82E-05	*SYT6/KCNB1/SCAMP5/CLTRN/SYT4/UNC13A/CADPS/SYT9/RIMS2/RIMS1/TRPV6/SYT5/CDK5R2/SYT8*	14
BP	GO:0008217	Regulation of blood pressure	25/917	183/18,800	3.12E-06	0.000136	0.000117	*SPX/P2RX1/ACSM3/NOS1/KL/CMA1/MYH6/UTS2R/ABAT/CARTPT/TACR1/KLK1/CTSG/ANPEP/TRHDE/AVPR1B/CNR1/NPY1R/AGTR2/GLP1R/CYP4A11/ADIPOQ/NPY/CHGA/KLK2*	25
BP	GO:0006936	Muscle contraction	38/917	349/18,800	3.24E-06	0.00014	0.000121	*SPX/MYH7/SCNN1B/P2RX1/KCNJ5/DTNA/MYLK2/NOS1/FGF12/MYH6/ABAT/GAMT/CHRM3/SLC8A3/TACR1/DMD/CACNB2/SLC8A1/TMOD1/SCN4A/KCND3/KIT/CHRNB2/GHRL/GNAO1/ANK2/SCN3B/SCN7A/KLHL41/SSTR2/MYBPC2/KCNA5/KCNJ3/CHRM2/HCN4/KCNB2/CHGA/GLRA1*	38
BP	GO:0021700	Developmental maturation	34/917	296/18,800	3.29E-06	0.000141	0.000121	*BHLHA15/NRCAM/SYBU/CX3CR1/RELN/ZBTB16/EPO/ADGRB3/PDE3A/NRXN1/RET/NEURL1/BCL11A/UNC13A/LRRK2/NKX6-1/CNTN2/SPINK1/SYP/MYOC/SEZ6/ASCL1/CFTR/IGF1/ACTL6B/TDRD1/CAMK2B/PTPRN/CCL21/CDK5R2/FEV/PAEP/FGG/CCL19*	34
BP	GO:0031644	Regulation of nervous system process	21/917	138/18,800	3.39E-06	0.000144	0.000124	*SPX/NLGN1/RELN/FGF12/VIP/ABAT/CARTPT/SLC8A3/SLC8A2/NRXN1/TNR/CELF4/GHRL/LRRK2/SHISA9/CBLN1/CACNG5/RIMS2/RIMS1/GLRA1/KLK8*	21
BP	GO:0006909	Phagocytosis	35/917	310/18,800	3.50E-06	0.000147	0.000127	*CD36/RAB39A/VAV3/XKR4/FCN2/CCR2/XKR7/IGHV3-64/PTPRC/SPACA3/C4BPA/IGHV6-1/IGHV2-5/IGHV3-74/ADIPOQ/IGHV4-4/PLPP4/IGHV3-20/IGHV3-7/IGHV3-15/IGHV1-45/IGHV3-72/IGHV3-48/IGHV2-26/IGHV3-49/IGHV4-28/IGHV5-51/CRP/IGHV1-69/IGHV4-39/IGHV4-59/IGHA2/IGHV3-21/IGHV3-13/IGHV1-18*	35
BP	GO:0050807	Regulation of synapse organization	27/917	209/18,800	3.77E-06	0.000157	0.000135	*NRCAM/CTTNBP2/NTRK2/AMIGO1/NLGN1/PAK3/RELN/KIF1A/ADGRB3/FLRT2/NRXN1/NEURL1/LRRTM2/CHRNB2/GHRL/SLITRK1/MUSK/LRRK2/C1QL3/CBLN1/CTNNA2/ITPKA/GPM6A/CAMK2B/LHFPL4/KLK8/LRRTM3*	27

 © 2023. The Author(s).

Supplementary Table S1 (*Continued*)

Ontology	ID	Description	GeneRatio	BgRatio	p-Value	*p*.adjust	q-Value	geneID	Count
BP	GO:0015872	Dopamine transport	12/917	51/18,800	4.60E-06	0.00019	0.000163	*SYT6/KCNA2/ABAT/CHRNB2/SYT4/SLC29A4/SYT9/SLC18A2/CXCL12/CNR1/SYT5/SYT8*	12
BP	GO:1903236	Regulation of leukocyte tethering or rolling	7/917	16/18,800	4.99E-06	0.000204	0.000176	*SELP/CCR2/ITGA4/SELE/CXCL12/CCL21/FUT9*	7
BP	GO:0002764	Immune response-regulating signaling pathway	47/917	482/18,800	5.08E-06	0.000206	0.000177	*GPLD1/REG3G/PAK3/CD36/FYB2/VAV3/PVRIG/THEMIS/KIT/TLR7/LAX1/CD8A/PRKCB/IGHV3-64/PTPRC/MS4A2/TLR8/IGHV6-1/IGHV2-5/IGHV3-74/BTLA/TLR10/SH2D1A/TRAT1/NR1H4/IGHV4-4/PLPP4/IGHV3-20/IGHV3-7/IGHV3-15/IGHV1-45/IGHV3-72/S100A14/IGHV3-48/IGHV2-26/IGHV3-49/IGHV4-28/MS4A1/IGHV5-51/IGHV1-69/IGHV4-39/IGHV4-59/BLK/IGHA2/IGHV3-21/IGHV3-13/IGHV1-18*	47
BP	GO:0009743	Response to carbohydrate	28/917	226/18,800	5.69E-06	0.000229	0.000197	*BRSK2/GPLD1/CMA1/KCNB1/SLC8A1/AQP4/CLTRN/GHRL/GRIK5/GPR27/NKX6-1/PCSK1/NEUROD1/NR0B2/PRKCB/CASR/RFX6/ZBED6/EPHA5/CFTR/GCK/NKX2-2/NR1H4/ADIPOQ/PTPRN/SLC30A8/PCK1/UCN3*	28
BP	GO:0001678	Cellular glucose homeostasis	22/917	154/18,800	5.78E-06	0.00023	0.000198	*BRSK2/GPLD1/CMA1/KLF15/CARTPT/KCNB1/AQP4/CLTRN/GHRL/GRIK5/GPR27/NEUROD1/CASR/RFX6/ZBED6/ABCC8/EPHA5/CFTR/GCK/NR1H4/PTPRN/PCK1*	22
BP	GO:0050803	Regulation of synapse structure or activity	27/917	215/18,800	6.45E-06	0.000255	0.000219	*NRCAM/CTTNBP2/NTRK2/AMIGO1/NLGN1/PAK3/RELN/KIF1A/ADGRB3/FLRT2/NRXN1/NEURL1/LRRTM2/CHRNB2/GHRL/SLITRK1/MUSK/LRRK2/C1QL3/CBLN1/CTNNA2/ITPKA/GPM6A/CAMK2B/LHFPL4/KLK8/LRRTM3*	27
BP	GO:0010975	Regulation of neuron projection development	43/917	431/18,800	7.22E-06	0.000283	0.000244	*BRSK2/NRCAM/NTRK2/SERPINI1/AMIGO1/NLGN1/RTN4RL1/PAK3/MAP2/RELN/ALKAL2/KIF1A/SEMA6D/KIAA0319/EPO/ADGRB3/PACSIN1/TNR/RET/CHRNB2/FSTL4/SLITRK1/BCL11A/MAPT/SFRP1/SEMA3E/LRRK2/CNTN2/DSCAM/ELAVL4/CSMD3/MIR210/CTNNA2/CXCL12/CNR1/SNAP25/LPAR3/ITPKA/SEZ6/CAMK2B/BMP5/KLK8/FUT9*	43
BP	GO:0014046	Dopamine secretion	10/917	37/18,800	7.59E-06	0.000293	0.000252	*SYT6/KCNA2/ABAT/CHRNB2/SYT4/SYT9/CXCL12/CNR1/SYT5/SYT8*	10
BP	GO:0014059	Regulation of dopamine secretion	10/917	37/18,800	7.59E-06	0.000293	0.000252	*SYT6/KCNA2/ABAT/CHRNB2/SYT4/SYT9/CXCL12/CNR1/SYT5/SYT8*	10
BP	GO:0016358	Dendrite development	28/917	231/18,800	8.65E-06	0.000331	0.000284	*CTNND2/NLGN1/PAK3/MAP2/RELN/KIF1A/KIAA0319/ADGRB3/GPR37/SDK1/PACSIN1/NEURL1/CHRNB2/FSTL4/DCDC2/GHRL/BCL11A/LRRK2/MAPK8IP2/DSCAM/ELAVL4/CSMD3/CTNNA2/ITPKA/SEZ6/ACTL6B/CAMK2B/BMP5*	28

(*Continued*)

 © 2023. The Author(s).

Supplementary Table S1 (*Continued*)

Ontology	ID	Description	GeneRatio	BgRatio	p-Value	*p*.adjust	q-Value	geneID	Count
BP	GO:0042310	Vasoconstriction	15/917	82/18,800	8.73E-06	0.000331	0.000285	*SCNN1B/P2RX1/CHRM3/TACR1/SLC6A4/SLC8A1/CYSLTR1/AVPR1B/KCNA5/KCNMB2/CASR/FGA/FGB/CRP/FGG*	15
BP	GO:0042593	Glucose homeostasis	29/917	244/18,800	8.83E-06	0.000332	0.000286	*BHLHA15/BRSK2/PDK4/GPLD1/CMA1/KLF15/CARTPT/KCNB1/AQP4/CLTRN/GHRL/GRIK5/GPR27/G6PC1/NEUROD1/CNR1/CASR/RFX6/ZBED6/ABCC8/EPHA5/CFTR/GCK/NR1H4/ADIPOQ/PTPRN/G6PC2/PCK1/FFAR1*	29
BP	GO:0010038	Response to metal ion	37/917	351/18,800	9.16E-06	0.000342	0.000294	*KCNK3/MT1F/SYT6/NLGN1/GPLD1/BNIP3/ADGRV1/ABAT/ADCY1/SLC30A2/MT1G/KIT/DLG2/SYT4/MAPT/CRHBP/DPEP1/LRRK2/CNGA3/PCSK1/SYT9/KCNMB2/OTC/MT1H/CASR/ABCC8/TRPV6/ASCL1/FGA/SYT5/FGB/GLRA1/FABP4/SLC30A8/MTTP/SYT8/FGG*	37
BP	GO:0033500	Carbohydrate homeostasis	29/917	245/18,800	9.56E-06	0.000354	0.000304	*BHLHA15/BRSK2/PDK4/GPLD1/CMA1/KLF15/CARTPT/KCNB1/AQP4/CLTRN/GHRL/GRIK5/GPR27/G6PC1/NEUROD1/CNR1/CASR/RFX6/ZBED6/ABCC8/EPHA5/CFTR/GCK/NR1H4/ADIPOQ/PTPRN/G6PC2/PCK1/FFAR1*	29
BP	GO:0030534	Adult behavior	20/917	136/18,800	9.77E-06	0.000359	0.000309	*SLC1A2/FGF12/RNF180/ABAT/CARTPT/SDK1/HOMER2/NRXN1/TMOD1/CHRNB2/GHRL/SLITRK1/CRHBP/CNTN2/CXCL12/GABRG2/SEZ6/NPY/GLRA1/PAX5*	20
BP	GO:0098815	Modulation of excitatory postsynaptic potential	10/917	39/18,800	1.27E-05	0.000462	0.000397	*NLGN1/RELN/SLC8A3/SLC8A2/NRXN1/CELF4/LRRK2/CBLN1/RIMS2/RIMS1*	10
BP	GO:0002443	Leukocyte mediated immunity	44/917	457/18,800	1.39E-05	0.000503	0.000432	*C5/PLA2G1B/SCNN1B/CX3CR1/CLU/C6/C7/KIT/CTSG/DNASE1L3/CCR2/C8A/CD8A/IGHV3-64/PTPRC/C4BPA/TLR8/IGHV6-1/IGHV2-5/IGHV3-74/CPLX2/SH2D1A/IGHV4-4/CHGA/IGHV3-20/IGHV3-7/IGHV3-15/IGHV1-45/IGHV3-72/IGHV3-48/APCS/IGHV2-26/IGHV3-49/IGHV4-28/IGHV5-51/CRP/IGHV1-69/IGHV4-39/IGHV4-59/BLK/IGHA2/IGHV3-21/IGHV3-13/IGHV1-18*	44
BP	GO:0007611	Learning or memory	30/917	264/18,800	1.51E-05	0.00054	0.000464	*PRKAR2B/GRPR/NTRK2/CX3CR1/PAK5/GATM/RELN/VIP/ADCY1/ADGRB3/SLC8A3/SLC8A2/TACR1/SLC6A4/NRXN1/TNR/KIT/CHRNB2/MUSK/SYT4/MAPT/CRHBP/CNTN2/MAPK8IP2/ELAVL4/CNR1/SNAP25/ABCC8/GLP1R/KLK8*	30

Supplementary Table S1 (*Continued*)

Ontology	ID	Description	GeneRatio	BgRatio	p-Value	*p*.adjust	q-Value	geneID	Count
BP	GO:1903305	Regulation of regulated secretory pathway	20/917	140/18,800	1.52E-05	0.00054	0.000464	*CHRNB3/P2RX1/SYT6/NLGN1/ADCY1/CACNB2/KCNB1/SCAMP5/SYT4/CCR2/LRRK2/SYP/SYT9/CNR1/PRKCB/TRPV6/SYT5/CDK5R2/SYT8/BLK*	20
BP	GO:0051954	Positive regulation of amine transport	10/917	40/18,800	1.62E-05	0.000571	0.000491	*VIP/ABAT/CARTPT/KCNB1/SLC38A3/CLTRN/CHRNB2/SYT4/AVPR1B/CXCL12*	10
BP	GO:0050901	Leukocyte tethering or rolling	9/917	33/18,800	2.01E-05	0.000704	0.000605	*CX3CR1/SELP/CCR2/ITGA4/MADCAM1/SELE/CXCL12/CCL21/FUT9*	9
BP	GO:0042113	B cell activation	35/917	336/18,800	2.05E-05	0.000707	0.000608	*FLT3/VAV3/KIT/CHRNB2/SFRP1/LAX1/ITGA4/PRKCB/IGHV3-64/ONECUT1/PTPRC/IGHV6-1/IGHV2-5/IGHV3-74/IGHV4-4/IGHV3-20/IGHV3-7/IGHV3-15/CXCR5/IGHV1-45/IGHV3-72/IGHV3-48/IGHV2-26/IGHV3-49/IGHV4-28/MS4A1/IGHV5-51/IGHV1-69/IGHV4-39/IGHV4-59/BLK/IGHA2/IGHV3-21/IGHV3-13/IGHV1-18*	35
BP	GO:0061564	Axon development	45/917	479/18,800	2.06E-05	0.000707	0.000608	*BRSK2/NRCAM/NTRK2/AMIGO1/RTN4RL1/PAK3/MAP2/RELN/SEMA6D/ADCY1/KIAA0319/NFASC/DSCAML1/NCAM1/FLRT2/NRXN1/TNR/RET/CHRNB2/OLFM1/FSTL4/GDF7/SLITRK1/MAPT/SEMA3E/NKX6-1/CNTN2/KIF5C/APLP1/DSCAM/ITGA4/CTNNA2/DPYSL5/CXCL12/CNR1/GFRA3/LPAR3/FEZF1/EPHA5/CDK5R2/CNTN5/ARX/PLP1/UNC5D/KLK8*	45
BP	GO:0042742	Defense response to bacterium	37/917	364/18,800	2.06E-05	0.000707	0.000608	*PLA2G1B/SCNN1B/REG3G/CD36/SELP/FCN2/CTSG/IGHV3-64/FGA/IGHV6-1/IGHV2-5/IGHV3-74/NR1H4/IGHV4-4/FGB/CHGA/IGHV3-20/IGHV3-7/IGHV3-15/IGHV1-45/JCHAIN/IGHV3-72/S100A14/IGHV3-48/IGHV2-26/IGHV3-49/IGHV4-28/ZG16/IGHV5-51/CRP/IGHV1-69/IGHV4-39/IGHV4-59/IGHA2/IGHV3-21/IGHV3-13/IGHV1-18*	37
BP	GO:1990573	Potassium ion import across plasma membrane	11/917	50/18,800	2.27E-05	0.000773	0.000665	*KCNJ5/KCNJ16/WNK2/FXYD2/ABCC9/KCNJ11/ATP1A3/KCNJ3/HCN4/ATP4A/KCNJ6*	11
BP	GO:0055067	Monovalent inorganic cation homeostasis	21/917	157/18,800	2.60E-05	0.000878	0.000755	*SPX/SCNN1B/PDK4/RAB39A/TACR1/SLC8A1/FASLG/SLC26A4/SLC4A4/SLC4A10/SCNN1G/LRRK2/CCKBR/SCN7A/ATP1A3/AVPR1B/KCNA5/OTC/ATP4A/CYP4A11/CFTR*	21
BP	GO:0045666	Positive regulation of neuron differentiation	15/917	91/18,800	3.19E-05	0.001062	0.000913	*NRCAM/NAP1L2/PROX1/SH3GL3/EPO/GDF7/NKX6-1/SOX11/NEUROD1/CXCL12/DUOXA1/PCP4/FEZF1/ASCL1/NKX2-2*	15

(*Continued*)

 © 2023. The Author(s).

Supplementary Table S1 (*Continued*)

Ontology	ID	Description	GeneRatio	BgRatio	p-Value	*p*.adjust	q-Value	geneID	Count
BP	GO:0070509	Calcium ion import	15/917	91/18,800	3.19E-05	0.001062	0.000913	*P2RX1/SLC8A3/SLC8A2/CACNB2/SLC8A1/EGF/SPINK1/CACNA1B/CXCL12/CASR/TRPV6/GCK/PLPP4/MRLN/MS4A1*	15
BP	GO:0031016	Pancreas development	14/917	81/18,800	3.37E-05	0.001113	0.000957	*PTF1A/PROX1/NR5A2/NKX6-1/PCSK1/NEUROD1/INSM1/ONECUT1/RFX6/ZBED6/FGF10/NKX2-2/BMP5/MSLN*	14
BP	GO:0007409	Axonogenesis	41/917	430/18,800	3.39E-05	0.001113	0.000957	*BRSK2/NRCAM/NTRK2/AMIGO1/PAK3/MAP2/RELN/SEMA6D/ADCY1/KIAA0319/NFASC/DSCAML1/NCAM1/FLRT2/NRXN1/TNR/RET/CHRNB2/OLFM1/FSTL4/GDF7/SLITRK1/MAPT/SEMA3E/NKX6-1/CNTN2/KIF5C/APLP1/DSCAM/ITGA4/CTNNA2/DPYSL5/CXCL12/GFRA3/LPAR3/FEZF1/EPHA5/CDK5R2/CNTN5/ARX/UNC5D*	41
BP	GO:0015849	Organic acid transport	33/917	318/18,800	3.77E-05	0.001229	0.001056	*SPX/SLC1A2/PLA2G1B/NTRK2/ABCB1/SLC7A2/CD36/ABAT/ABCD2/SLC38A3/SLCO1C1/SLC26A4/NPY5R/CLTRN/SYT4/SLC6A15/SLC7A14/SLC29A4/SLC38A11/SLC22A9/SLC3A1/SLC38A4/NR0B2/AVPR1B/SLC5A8/SLC10A2/RBP2/CYP4A11/PMP2/SLC6A17/FABP4/PLA2G2D/SLC26A9*	33
BP	GO:0050890	Cognition	32/917	306/18,800	4.23E-05	0.00137	0.001178	*PRKAR2B/GRPR/NTRK2/CX3CR1/PAK5/GATM/RELN/VIP/ADCY1/ADGRB3/SLC8A3/SLC8A2/TACR1/SLC6A4/TUSC3/NRXN1/TNR/KIT/CHRNB2/FAM107A/MUSK/SYT4/MAPT/CRHBP/CNTN2/MAPK8IP2/ELAVL4/CNR1/SNAP25/ABCC8/GLP1R/KLK8*	32
BP	GO:0034329	Cell junction assembly	40/917	420/18,800	4.33E-05	0.001389	0.001195	*ERBB4/NRCAM/CEL/NTRK2/CTNND2/AMIGO1/NLGN1/MPP7/GABRB3/ADGRB3/SDK1/FLRT2/BSN/NRXN1/PCDHB5/PCLO/LRRTM2/CHRNB2/GHRL/SLITRK1/FAM107A/MUSK/CDH22/SFRP1/ANK2/DSCAM/CBLN1/IGFN1/MYOC/GABRG2/GPM6A/CDH10/CBLN4/NR1H4/CNTN5/CDH19/GABRA2/LHFPL4/COL17A1/LRRTM3*	40
BP	GO:0009991	Response to extracellular stimulus	44/917	479/18,800	4.35E-05	0.001389	0.001195	*BHLHA15/SPX/SLC1A2/BRSK2/ELAPOR1/ACACB/SCNN1B/PDK4/CLPS/PROX1/BNIP3/ALB/KL/EPO/CARTPT/KCNB1/SLC6A4/SLC8A1/SLC38A3/GHRL/FAM107A/SFRP1/LRRK2/CNTN2/SPINK1/PCSK1/ITGA4/SSTR2/OTC/CNR1/CBS/CASR/SSTR3/PTPRC/ASCL1/SLC39A5/NR1H4/ADIPOQ/NPY/CLPSL1/PCK1/UCN3/SCTR/SST*	44

Supplementary Table S1 (*Continued*)

Ontology	ID	Description	GeneRatio	BgRatio	p-Value	*p*.adjust	q-Value	geneID	Count
BP	GO:0034764	Positive regulation of transmembrane transport	25/917	213/18,800	4.44E-05	0.001409	0.001211	*SLC1A2/P2RX1/ABCB1/AMIGO1/NOS1/AKAP6/RELN/WNK2/RGN/KLF15/FXYD2/DMD/CACNB2/PIRT/CLTRN/PDZK1/ANK2/CCR2/MIR210/ABCC8/CFTR/IGF1/ADIPOQ/CXCL9/PLP1*	25
BP	GO:0034767	Positive regulation of ion transmembrane transport	21/917	163/18,800	4.57E-05	0.001436	0.001235	*P2RX1/ABCB1/AMIGO1/NOS1/AKAP6/RELN/WNK2/RGN/FXYD2/DMD/CACNB2/PIRT/CLTRN/PDZK1/ANK2/CCR2/MIR210/ABCC8/CFTR/CXCL9/PLP1*	21
BP	GO:0051223	Regulation of protein transport	45/917	495/18,800	4.59E-05	0.001436	0.001235	*MYRIP/BRSK2/PLA2G1B/CELA2A/GPLD1/CD36/ABAT/CARTPT/KCNB1/GHRL/PDZK1/SYT4/GPR27/SFRP1/LRRK2/NKX6-1/KCNJ11/PCSK1/STXBP5L/NEUROD1/SYT9/NR0B2/KCNA5/CNR1/SNAP25/CASR/RFX6/ZBED6/ABCC8/FGA/EPHA5/CFTR/GCK/NR1H4/IGF1/ADIPOQ/FGB/CHGA/G6PC2/SLC30A8/SFN/FFAR1/UCN3/BLK/FGG*	45
BP	GO:0001764	Neuron migration	21/917	164/18,800	5.01E-05	0.001555	0.001337	*NRCAM/NTRK2/NEXMIF/RELN/KIAA0319/FLRT2/DCDC2/ASTN1/NKX6-1/CNTN2/CTNNA2/CXCL12/GFRA3/FEZF1/GPM6A/DCX/ASCL1/CAMK2B/CDK5R2/ARX/UNC5D*	21
BP	GO:0140115	Export across plasma membrane	13/917	74/18,800	5.35E-05	0.001651	0.001419	*ABCB1/KCNA2/SLC8A2/FXYD2/KCNB1/SLC8A1/KCND3/SLC4A4/PDZK1/SLC29A4/ATP1A3/KCNA5/ATP4A*	13
BP	GO:0050878	Regulation of body fluid levels	37/917	382/18,800	5.82E-05	0.001784	0.001533	*ERBB4/CELA2A/CEL/SCNN1B/P2RX1/F11/CD36/VIP/ABAT/CDO1/VAV3/CHRM3/TACR1/MMRN1/P2RY12/KLKB1/AQP4/SELP/PRLR/NEURL1/VTN/SCNN1G/CTSG/APOH/TFPI2/SPINK1/SERPINA10/FGA/CYP4A11/CFTR/FGF10/FGB/KRT1/SFN/KRT16/BLK/FGG*	37
BP	GO:0002440	Production of molecular mediator of immune response	32/917	312/18,800	6.16E-05	0.001876	0.001613	*CD36/KIT/CCR2/IGKV2-28/IGKV2D-28/PTPRC/IGLV5-37/IGLV7-46/IGLV7-43/IGKV1D-39/IGKV1-12/IGKV1-39/IGKV5-2/IGLV2-8/IGKV1D-13/IGLV2-18/IGKV1-17/IGKV2-24/IGKV1-6/IGLV4-60/IGLV6-57/IGKV1-16/IGLV4-69/IGLV10-54/IGLV3-27/IGLV5-45/IGKV4-1/IGLV3-19/IGKV3D-20/IGKV1-27/IGKV1-9/IGLV8-61*	32
BP	GO:0007626	Locomotory behavior	23/917	192/18,800	6.45E-05	0.001951	0.001677	*SELENOP/PAK5/RELN/FGF12/ABAT/GPR37/TMOD1/TNR/USP2/CHRNB2/SLC4A10/ASTN1/LRRK2/MCOLN3/CNTN2/DSCAM/ELAVL4/SLC18A2/CXCL12/SNAP25/NPY1R/SEZ6/GLRA1*	23
BP	GO:1904862	Inhibitory synapse assembly	6/917	16/18,800	6.96E-05	0.002091	0.001798	*GABRB3/CBLN1/GABRG2/CBLN4/GABRA2/LHFPL4*	6
BP	GO:0061045	Negative regulation of wound healing	13/917	76/18,800	7.14E-05	0.002131	0.001832	*F11/ABAT/MMRN1/KLKB1/VTN/AJAP1/APOH/ABCC8/FGA/FGB/KRT1/APCS/FGG*	13

(*Continued*)

 © 2023. The Author(s).

Global Medical Genetics © 2023. The Author(s).

Supplementary Table S1 (*Continued*)

Ontology	ID	Description	GeneRatio	BgRatio	p-Value	*p*.adjust	q-Value	geneID	Count
BP	GO:0022898	Regulation of transmembrane transporter activity	29/917	273/18,800	7.21E-05	0.002138	0.001838	*PM20D1/ABCB1/AMIGO1/NLGN1/KCNAB1/NOS1/AKAP6/RELN/FGF12/WNK2/RGN/CHRM3/EPO/FXYD2/DMD/CACNB2/PIRT/CLTRN/PDZK1/CRHBP/ANK2/CCR2/SHISA9/SCN3B/MAPK8IP2/CACNG5/ABCC8/CFTR/MRLN*	29
BP	GO:1902476	Chloride transmembrane transport	12/917	66/18,800	7.27E-05	0.002144	0.001843	*GABRB3/ANO5/ANO3/SLC26A4/GLRA3/GABRA4/CASR/GABRG2/CFTR/GLRA1/GABRA2/SLC26A9*	12
BP	GO:0071333	Cellular response to glucose stimulus	18/917	132/18,800	7.45E-05	0.002179	0.001874	*BRSK2/GPLD1/CMA1/KCNB1/AQP4/CLTRN/GHRL/GRIK5/GPR27/NEUROD1/CASR/RFX6/ZBED6/EPHA5/CFTR/NR1H4/PTPRN/PCK1*	18
BP	GO:0051592	Response to calcium ion	19/917	144/18,800	7.49E-05	0.002179	0.001874	*SYT6/NLGN1/GPLD1/ADGRV1/ADCY1/SYT4/CRHBP/DPEP1/PCSK1/SYT9/KCNMB2/CASR/TRPV6/FGA/SYT5/FGB/MTTP/SYT8/FGG*	19
BP	GO:0061337	Cardiac conduction	15/917	98/18,800	7.74E-05	0.002238	0.001924	*KCNJ5/SLC8A3/SLC8A2/CACNB2/SLC8A1/KCND3/ATP2B2/ABCC9/ANK2/SCN3B/ATP1A3/KCNA5/KCNJ3/HCN4/KCNH6*	15
BP	GO:0051339	Regulation of lyase activity	13/917	77/18,800	8.21E-05	0.002359	0.002028	*GUCA1C/VIPR2/NOS1/ADGRV1/GALR1/TSHR/ADCYAP1/P2RY13/CCR2/MIR210/GLP1R/GUCA2A/GPR87*	13
BP	GO:0007187	G protein-coupled receptor signaling pathway, coupled to cyclic nucleotide second messenger	12/917	67/18,800	8.47E-05	0.002407	0.00207	*MTNR1A/CHRM3/TSHR/SSTR2/CHRM2/CNR1/NPY1R/AGTR2/LPAR3/SSTR3/XCR1/NPY*	12
BP	GO:0086091	Regulation of heart rate by cardiac conduction	9/917	39/18,800	8.48E-05	0.002407	0.00207	*KCNJ5/CACNB2/KCND3/ANK2/SCN3B/KCNA5/KCNJ3/HCN4/KCNH6*	9
BP	GO:0071331	Cellular response to hexose stimulus	18/917	134/18,800	9.08E-05	0.002562	0.002203	*BRSK2/GPLD1/CMA1/KCNB1/AQP4/CLTRN/GHRL/GRIK5/GPR27/NEUROD1/CASR/RFX6/ZBED6/EPHA5/CFTR/NR1H4/PTPRN/PCK1*	18
BP	GO:0060402	Calcium ion transport into cytosol	21/917	171/18,800	9.24E-05	0.002592	0.002229	*PLA2G1B/P2RX1/NOS1/AKAP6/EPO/SLC8A3/SLC8A2/DMD/SLC8A1/FASLG/ANK2/MCOLN3/CACNA1B/CCR5/PTPRC/TRPV6/XCR1/CCL21/CXCL9/MS4A1/CCL19*	21
BP	GO:0071322	Cellular response to carbohydrate stimulus	19/917	147/18,800	9.91E-05	0.002764	0.002376	*BRSK2/GPLD1/CMA1/KCNB1/AQP4/CLTRN/GHRL/GRIK5/GPR27/NEUROD1/PRKCB/CASR/RFX6/ZBED6/EPHA5/CFTR/NR1H4/PTPRN/PCK1*	19

Supplementary Table S1 (*Continued*)

Ontology	ID	Description	GeneRatio	BgRatio	p-Value	*p*.adjust	q-Value	geneID	Count
BP	GO:0008016	Regulation of heart contraction	24/917	211/18,800	0.000103	0.002807	0.002413	*SPX/MYH7/KCNJ5/NOS1/MYH6/SLC8A3/SLC8A2/DMD/CACNB2/SLC8A1/KCND3/ATP2B2/ABCC9/ANK2/SCN3B/ATP1A3/KCNA5/KCNJ3/CHRM2/HCN4/AGTR2/KCNH6/GLP1R/CHGA*	24
BP	GO:0050679	Positive regulation of epithelial cell proliferation	24/917	211/18,800	0.000103	0.002807	0.002413	*PROX1/REG3G/VIP/STOX1/TACR1/REG3A/EGF/GHRL/SFRP1/HPN/SPINK1/SOX11/ITGA4/ZBED9/VEGFD/CXCL12/SCG2/THBS4/PKHD1/FGF10/IGF1/BMP5/PTPRN/FGFBP1*	24
BP	GO:0007158	Neuron cell–cell adhesion	6/917	17/18,800	0.000103	0.002807	0.002413	*CEL/NLGN1/NRXN1/TNR/RET/ASTN1*	6
BP	GO:0048791	Calcium ion-regulated exocytosis of neurotransmitter	6/917	17/18,800	0.000103	0.002807	0.002413	*SYT4/UNC13A/RIMS2/RIMS1/SYT5/SYT8*	6
BP	GO:0017158	Regulation of calcium ion-dependent exocytosis	9/917	40/18,800	0.000105	0.002834	0.002436	*SYT6/KCNB1/SCAMP5/SYT4/SYT9/TRPV6/SYT5/CDK5R2/SYT8*	9
BP	GO:0042730	Fibrinolysis	7/917	24/18,800	0.000107	0.002879	0.002475	*F11/KLKB1/APOH/FGA/FGB/KRT1/FGG*	7
BP	GO:1904064	Positive regulation of cation transmembrane transport	19/917	148/18,800	0.000109	0.002907	0.002499	*P2RX1/AMIGO1/NOS1/AKAP6/RELN/WNK2/RGN/FXYD2/DMD/CACNB2/PIRT/CLTRN/PDZK1/ANK2/CCR2/MIR210/ABCC8/CXCL9/PLP1*	19
BP	GO:0071326	Cellular response to monosaccharide stimulus	18/917	136/18,800	0.00011	0.002931	0.00252	*BRSK2/GPLD1/CMA1/KCNB1/AQP4/CLTRN/GHRL/GRIK5/GPR27/NEUROD1/CASR/RFX6/ZBED6/EPHA5/CFTR/NR1H4/PTPRN/PCK1*	18
BP	GO:0002449	Lymphocyte mediated immunity	35/917	365/18,800	0.000112	0.002949	0.002536	*C5/CLU/C6/C7/CCR2/C8A/CD8A/IGHV3-64/PTPRC/C4BPA/TLR8/IGHV6-1/IGHV2-5/IGHV3-74/SH2D1A/IGHV4-4/IGHV3-20/IGHV3-7/IGHV3-15/IGHV1-45/IGHV3-72/IGHV3-48/APCS/IGHV2-26/IGHV3-49/IGHV4-28/IGHV5-51/CRP/IGHV1-69/IGHV4-39/IGHV4-59/IGHA2/IGHV3-21/IGHV3-13/IGHV1-18*	35
BP	GO:0035773	Insulin secretion involved in cellular response to glucose stimulus	11/917	59/18,800	0.000114	0.002998	0.002578	*BRSK2/GPLD1/CLTRN/GHRL/GPR27/RFX6/ZBED6/EPHA5/CFTR/NR1H4/PTPRN*	11
BP	GO:0050806	Positive regulation of synaptic transmission	20/917	161/18,800	0.000115	0.003002	0.002581	*NTRK2/NLGN1/CX3CR1/RELN/ADCY1/SLC8A3/SLC8A2/TACR1/CACNB2/NRXN1/TNR/LRRTM2/FAM107A/CCR2/SCGN/CACNG5/CNR1/RIMS2/SNAP25/RIMS1*	20

(*Continued*)

 © 2023. The Author(s).

Global Medical Genetics © 2023. The Author(s).

Supplementary Table S1 (*Continued*)

Ontology	ID	Description	GeneRatio	BgRatio	p-Value	*p*.adjust	q-Value	geneID	Count
BP	GO:0031639	Plasminogen activation	7/917	25/18,800	0.000142	0.003681	0.003165	*F11/KLKB1/APOH/HPN/FGA/FGB/FGG*	7
BP	GO:0060074	Synapse maturation	7/917	25/18,800	0.000142	0.003681	0.003165	*SYBU/CX3CR1/RELN/NRXN1/NEURL1/SEZ6/CAMK2B*	7
BP	GO:0002460	Adaptive immune response based on somatic recombination of immune receptors built from immunoglobulin superfamily domains	35/917	370/18,800	0.000146	0.003744	0.003219	*C5/CLU/C6/C7/CCR2/C8A/CD8A/IGHV3-64/PTPRC/C4BPA/TLR8/IGHV6-1/IGHV2-5/IGHV3-74/IGHV4-4/IGHV3-20/IGHV3-7/IGHV3-15/IGHV1-45/IGHV3-72/IGHV3-48/APCS/IGHV2-26/IGHV3-49/IGHV4-28/IGHV5-51/CRP/IGHV1-69/IGHV4-39/IGHV4-59/IGHA2/IGHV3-21/IGHV3-13/CCL19/IGHV1-18*	35
BP	GO:0051251	Positive regulation of lymphocyte activation	35/917	371/18,800	0.000153	0.00392	0.00337	*VAV3/ZBTB16/EPO/TACR1/CHRNB2/CCR2/TNFSF9/IGHV3-64/PTPRC/IGHV6-1/IGHV2-5/IGHV3-74/FGF10/IGF1/IGHV4-4/IGHV3-20/IGHV3-7/IGHV3-15/CCL21/IGHV1-45/IGHV3-72/PCK1/IGHV3-48/IGHV2-26/IGHV3-49/IGHV4-28/IGHV5-51/IGHV1-69/IGHV4-39/IGHV4-59/IGHA2/IGHV3-21/IGHV3-13/CCL19/IGHV1-18*	35
BP	GO:0042445	Hormone metabolic process	25/917	230/18,800	0.000154	0.00392	0.00337	*SCNN1B/GCNT4/PNLIP/MBOAT4/CHST9/CMA1/ADH1A/SLCO1C1/ADH1B/CTSG/HPN/PCSK1/UGT2B15/TPO/PRLHR/CPE/UGT2B11/CYP2C8/DUOXA1/PCSK2/SCG5/BMP5/TTR/UGT2B17/SLC30A8*	25
BP	GO:0048488	Synaptic vesicle endocytosis	11/917	61/18,800	0.000156	0.00392	0.00337	*CEL/NLGN1/DNAJC6/SH3GL3/PACSIN1/LRRK2/SYP/SNAP91/SH3GL2/SYT5/SYT8*	11
BP	GO:0140238	Presynaptic endocytosis	11/917	61/18,800	0.000156	0.00392	0.00337	*CEL/NLGN1/DNAJC6/SH3GL3/PACSIN1/LRRK2/SYP/SNAP91/SH3GL2/SYT5/SYT8*	11
BP	GO:0031667	Response to nutrient levels	40/917	446/18,800	0.000159	0.003985	0.003426	*BHLHA15/SPX/BRSK2/ELAPOR1/ACACB/SCNN1B/PDK4/CLPS/PROX1/BNIP3/ALB/KL/EPO/CARTPT/KCNB1/SLC6A4/SLC8A1/SLC38A3/GHRL/FAM107A/SFRP1/LRRK2/CNTN2/SPINK1/PCSK1/SSTR2/OTC/CNR1/CASR/SSTR3/ASCL1/SLC39A5/NR1H4/ADIPOQ/NPY/CLPSL1/PCK1/UCN3/SCTR/SST*	40
BP	GO:0002027	Regulation of heart rate	15/917	105/18,800	0.000172	0.00427	0.003671	*SPX/MYH7/KCNJ5/MYH6/DMD/CACNB2/SLC8A1/KCND3/ANK2/SCN3B/KCNA5/KCNJ3/HCN4/AGTR2/KCNH6*	15
BP	GO:1903034	Regulation of response to wounding	20/917	166/18,800	0.000175	0.004291	0.003689	*F11/RTN4RL1/REG3G/CD36/ABAT/KIAA0319/MMRN1/REG3A/KLKB1/TNR/VTN/AJAP1/APOH/ABCC8/FGA/FGB/KRT1/APCS/KLK8/FGG*	20

Supplementary Table S1 (*Continued*)

Ontology	ID	Description	GeneRatio	BgRatio	p-Value	*p*.adjust	q-Value	geneID	Count
BP	GO:0035296	Regulation of tube diameter	18/917	141/18,800	0.000175	0.004291	0.003689	*SCNN1B/P2RX1/NOS1/UTS2R/CHRM3/TACR1/SLC6A4/SLC8A1/CYSLTR1/AVPR1B/KCNA5/KCNMB2/AGTR2/CASR/FGA/FGB/CRP/FGG*	18
BP	GO:0097746	Blood vessel diameter maintenance	18/917	141/18,800	0.000175	0.004291	0.003689	*SCNN1B/P2RX1/NOS1/UTS2R/CHRM3/TACR1/SLC6A4/SLC8A1/CYSLTR1/AVPR1B/KCNA5/KCNMB2/AGTR2/CASR/FGA/FGB/CRP/FGG*	18
BP	GO:0045664	Regulation of neuron differentiation	22/917	192/18,800	0.000177	0.004291	0.003689	*NRCAM/NAP1L2/NLGN1/PROX1/SH3GL3/EPO/SLC6A4/RET/GDF7/BCL11A/SFRP1/LRRK2/NKX6-1/CNTN2/SOX11/NEUROD1/CXCL12/DUOXA1/PCP4/FEZF1/ASCL1/NKX2-2*	22
BP	GO:0032411	Positive regulation of transporter activity	16/917	117/18,800	0.000178	0.004291	0.003689	*ABCB1/AMIGO1/AKAP6/RELN/WNK2/RGN/FXYD2/DMD/CACNB2/PIRT/PDZK1/ANK2/CCR2/ABCC8/CFTR/ADIPOQ*	16
BP	GO:0035249	Synaptic transmission, glutamatergic	14/917	94/18,800	0.000179	0.004291	0.003689	*P2RX1/NLGN1/RELN/NRXN1/TNR/GRIK5/UNC13A/CCR2/LRRK2/MAPK8IP2/CACNG5/GRIA2/CNR1/GRM4*	14
BP	GO:1901606	Alpha-amino acid catabolic process	14/917	94/18,800	0.000179	0.004291	0.003689	*GLS2/TDH/NOS1/CDO1/HOGA1/PAH/HPD/ASPA/HMGCLL1/MAT1A/PRODH2/OTC/CBS/GAD2*	14
BP	GO:0045921	Positive regulation of exocytosis	13/917	83/18,800	0.00018	0.004304	0.0037	*NLGN1/CACNB2/KCNB1/SCAMP5/SYT4/CADPS/SYT9/CNR1/FGA/CFTR/FGB/CDK5R2/FGG*	13
BP	GO:0016486	Peptide hormone processing	8/917	34/18,800	0.000182	0.004312	0.003707	*MBOAT4/CMA1/CTSG/PCSK1/CPE/PCSK2/SCG5/SLC30A8*	8
BP	GO:0032414	Positive regulation of ion transmembrane transporter activity	15/917	106/18,800	0.000191	0.004509	0.003877	*ABCB1/AMIGO1/AKAP6/RELN/WNK2/RGN/FXYD2/DMD/CACNB2/PIRT/PDZK1/ANK2/CCR2/ABCC8/CFTR*	15
BP	GO:0035150	Regulation of tube size	18/917	142/18,800	0.000192	0.004509	0.003877	*SCNN1B/P2RX1/NOS1/UTS2R/CHRM3/TACR1/SLC6A4/SLC8A1/CYSLTR1/AVPR1B/KCNA5/KCNMB2/AGTR2/CASR/FGA/FGB/CRP/FGG*	18
BP	GO:0060047	Heart contraction	26/917	247/18,800	0.000193	0.004521	0.003887	*SPX/MYH7/KCNJ5/MYLK2/NOS1/FGF12/MYH6/SLC8A3/SLC8A2/DMD/CACNB2/SLC8A1/KCND3/ATP2B2/ABCC9/ANK2/SCN3B/ATP1A3/KCNA5/KCNJ3/CHRM2/HCN4/AGTR2/KCNH6/GLP1R/CHGA*	26
BP	GO:0015711	Organic anion transport	34/917	361/18,800	0.000196	0.004549	0.003911	*SLC1A2/PLA2G1B/NTRK2/SLC16A12/SLC7A2/CA4/SLCO4C1/CD36/ABAT/SLC38A3/SLCO1C1/SLC2A2/SLC26A4/NPY5R/SLC4A4/CLTRN/SLC4A10/SLC17A4/SYT4/SLC6A15/SLC17A1/SLC22A9/SLC3A1/NR0B2/AVPR1B/SLC5A8/SLC10A2/CASR/CYP4A11/CFTR/SLC6A17/MTTP/PLA2G2D/SLC26A9*	34

(*Continued*)

 © 2023. The Author(s).

Global Medical Genetics © 2023. The Author(s).

Supplementary Table S1 (*Continued*)

Ontology	ID	Description	GeneRatio	BgRatio	p-Value	*p*.adjust	q-Value	geneID	Count
BP	GO:0050867	Positive regulation of cell activation	39/917	436/18,800	0.000202	0.004663	0.004009	*VAV3/ZBTB16/EPO/TACR1/SELP/CHRNB2/CCR2/LRRK2/IL33/TNFSF9/IGHV3-64/PTPRC/SPACA3/IGHV6-1/IGHV2-5/IGHV3-74/FGF10/IGF1/IGHV4-4/IGHV3-20/IGHV3-7/IGHV3-15/CCL21/IGHV1-45/IGHV3-72/PCK1/IGHV3-48/IGHV2-26/IGHV3-49/IGHV4-28/IGHV5-51/IGHV1-69/IGHV4-39/IGHV4-59/IGHA2/IGHV3-21/IGHV3-13/CCL19/IGHV1-18*	39
BP	GO:0002696	Positive regulation of leukocyte activation	38/917	421/18,800	0.000203	0.004663	0.004009	*VAV3/ZBTB16/EPO/TACR1/CHRNB2/CCR2/LRRK2/IL33/TNFSF9/IGHV3-64/PTPRC/SPACA3/IGHV6-1/IGHV2-5/IGHV3-74/FGF10/IGF1/IGHV4-4/IGHV3-20/IGHV3-7/IGHV3-15/CCL21/IGHV1-45/IGHV3-72/PCK1/IGHV3-48/IGHV2-26/IGHV3-49/IGHV4-28/IGHV5-51/IGHV1-69/IGHV4-39/IGHV4-59/IGHA2/IGHV3-21/IGHV3-13/CCL19/IGHV1-18*	38
BP	GO:0006837	Serotonin transport	6/917	19/18,800	0.000208	0.004669	0.004014	*P2RX1/NOS1/SLC6A4/SLC29A4/SLC18A2/CNR1*	6
BP	GO:0010738	Regulation of protein kinase A signaling	6/917	19/18,800	0.000208	0.004669	0.004014	*AKAP6/ADGRV1/AKAP7/LRRK2/ADIPOQ/IAPP*	6
BP	GO:0086014	Atrial cardiac muscle cell action potential	6/917	19/18,800	0.000208	0.004669	0.004014	*KCNJ5/CACNB2/ANK2/SCN3B/KCNA5/KCNJ3*	6
BP	GO:0086026	Atrial cardiac muscle cell to AV node cell signaling	6/917	19/18,800	0.000208	0.004669	0.004014	*KCNJ5/CACNB2/ANK2/SCN3B/KCNA5/KCNJ3*	6
BP	GO:0086066	Atrial cardiac muscle cell to AV node cell communication	6/917	19/18,800	0.000208	0.004669	0.004014	*KCNJ5/CACNB2/ANK2/SCN3B/KCNA5/KCNJ3*	6
BP	GO:0051965	Positive regulation of synapse assembly	11/917	63/18,800	0.00021	0.004695	0.004037	*NTRK2/AMIGO1/NLGN1/ADGRB3/FLRT2/NRXN1/LRRTM2/GHRL/SLITRK1/CBLN1/LRRTM3*	11
BP	GO:0032409	Regulation of transporter activity	30/917	305/18,800	0.000217	0.004824	0.004148	*PM20D1/ABCB1/AMIGO1/NLGN1/KCNAB1/NOS1/AKAP6/RELN/FGF12/WNK2/RGN/CHRM3/EPO/FXYD2/DMD/CACNB2/PIRT/CLTRN/PDZK1/CRHBP/ANK2/CCR2/SHISA9/SCN3B/MAPK8IP2/CACNG5/ABCC8/CFTR/ADIPOQ/MRLN*	30
BP	GO:0032412	Regulation of ion transmembrane transporter activity	27/917	263/18,800	0.000222	0.004907	0.004219	*ABCB1/AMIGO1/NLGN1/KCNAB1/NOS1/AKAP6/RELN/FGF12/WNK2/RGN/CHRM3/EPO/FXYD2/DMD/CACNB2/PIRT/PDZK1/CRHBP/ANK2/CCR2/SHISA9/SCN3B/MAPK8IP2/CACNG5/ABCC8/CFTR/MRLN*	27

Supplementary Table S1 (*Continued*)

Ontology	ID	Description	GeneRatio	BgRatio	p-Value	*p*.adjust	q-Value	geneID	Count
BP	GO:0006937	Regulation of muscle contraction	20/917	170/18,800	0.000241	0.005266	0.004527	*SPX/MYH7/P2RX1/MYLK2/NOS1/ABAT/CHRM3/SLC8A3/TACR1/DMD/SLC8A1/SCN4A/KIT/GHRL/ANK2/SSTR2/CHRM2/HCN4/KCNB2/CHGA*	20
BP	GO:0048469	Cell maturation	21/917	183/18,800	0.000241	0.005266	0.004527	*BHLHA15/NRCAM/EPO/ADGRB3/PDE3A/RET/BCL11A/LRRK2/NKX6-1/CNTN2/SPINK1/MYOC/ASCL1/CFTR/ACTL6B/TDRD1/CCL21/FEV/PAEP/FGG/CCL19*	21
BP	GO:0019229	Regulation of vasoconstriction	11/917	64/18,800	0.000243	0.005266	0.004527	*P2RX1/CHRM3/TACR1/CYSLTR1/AVPR1B/KCNA5/KCNMB2/CASR/FGA/FGB/FGG*	11
BP	GO:0031638	Zymogen activation	11/917	64/18,800	0.000243	0.005266	0.004527	*CUZD1/F11/KLKB1/KLK1/APOH/HPN/FGA/PRSS3/FGB/KLK2/FGG*	11
BP	GO:0051960	Regulation of nervous system development	39/917	440/18,800	0.000243	0.005266	0.004527	*NTRK2/AMIGO1/NLGN1/CX3CR1/PAK3/MAP2/RELN/SEMA6D/KIAA0319/ADGRB3/FLRT2/NRXN1/TNR/NEURL1/LRRTM2/KIT/FSTL4/GHRL/ELAPOR2/SLITRK1/ASPA/SYT4/MAPT/SEMA3E/NKX6-1/SOX11/DSCAM/CBLN1/CXCL12/LPAR3/FEZF1/ITPKA/ABCC8/ASCL1/RXRG/NKX2-2/CAMK2B/KLK8/LRRTM3*	39
BP	GO:0006968	Cellular defense response	10/917	54/18,800	0.000246	0.005287	0.004545	*UMOD/CX3CR1/PNLIPRP2/DCDC2/CCR2/IL33/CCR5/SH2D1A/TRAT1/CXCL9*	10
BP	GO:0033605	Positive regulation of catecholamine secretion	5/917	13/18,800	0.000253	0.005423	0.004663	*VIP/CARTPT/KCNB1/CHRNB2/CXCL12*	5
BP	GO:0090066	Regulation of anatomical structure size	42/917	487/18,800	0.000257	0.005476	0.004708	*NRCAM/SCNN1B/P2RX1/NPHS1/NOS1/PAK3/MAP2/UTS2R/SEMA6D/VAV3/KIAA0319/CHRM3/SPTB/TACR1/SLC6A4/SLC8A1/TMOD1/AQP4/TNR/RET/CYSLTR1/OLFM1/FSTL4/MAPT/SEMA3E/LRRK2/NKX6-1/CNTN2/DSCAM/AVPR1B/KCNA5/CXCL12/KCNMB2/AGTR2/LPAR3/CASR/FGA/FGB/CCL21/SCTR/CRP/FGG*	42
BP	GO:1904062	Regulation of cation transmembrane transport	33/917	352/18,800	0.000263	0.005575	0.004793	*P2RX1/PM20D1/AMIGO1/NLGN1/KCNAB1/NOS1/AKAP6/RELN/FGF12/WNK2/RGN/DPP10/EPO/FXYD2/DMD/CACNB2/SLC8A1/PIRT/CLTRN/PDZK1/CRHBP/ANK2/CCR2/SHISA9/SCN3B/MAPK8IP2/MIR210/CACNG5/DPP6/ABCC8/MRLN/CXCL9/PLP1*	33
BP	GO:0042551	Neuron maturation	9/917	45/18,800	0.000273	0.005761	0.004953	*NRCAM/ADGRB3/RET/BCL11A/LRRK2/CNTN2/MYOC/ACTL6B/FEV*	9
BP	GO:0086010	Membrane depolarization during action potential	8/917	36/18,800	0.000278	0.005815	0.005	*SCN3A/CACNB2/SLC8A1/SCN4A/ANK2/SCN3B/SCN7A/HCN4*	8
BP	GO:0140448	Signaling receptor ligand precursor processing	8/917	36/18,800	0.000278	0.005815	0.005	*MBOAT4/CMA1/CTSG/PCSK1/CPE/PCSK2/SCG5/SLC30A8*	8

(*Continued*)

Supplementary Table S1 (*Continued*)

Ontology	ID	Description	GeneRatio	BgRatio	p-Value	*p*.adjust	q-Value	geneID	Count
BP	GO:0010232	Vascular transport	13/917	87/18,800	0.000292	0.006053	0.005204	*SLC1A2/ABCB1/SLC16A12/SLC7A2/CD36/SLC8A2/SLC38A3/SLCO1C1/SLC4A4/ABCC9/SLC29A4/AVPR1B/SLC6A17*	13
BP	GO:0150104	Transport across blood–brain barrier	13/917	87/18,800	0.000292	0.006053	0.005204	*SLC1A2/ABCB1/SLC16A12/SLC7A2/CD36/SLC8A2/SLC38A3/SLCO1C1/SLC4A4/ABCC9/SLC29A4/AVPR1B/SLC6A17*	13
BP	GO:0048588	Developmental cell growth	24/917	227/18,800	0.000314	0.006496	0.005585	*NRCAM/SORBS2/AKAP6/MAP2/SEMA6D/KIAA0319/TNR/OLFM1/FSTL4/BCL11A/SYT4/MAPT/SEMA3E/UNC13A/SPAG6/NKX6-1/DSCAM/ITGA4/CXCL12/RIMS2/RIMS1/SH3GL2/LPAR3/IGF1*	24
BP	GO:0006821	Chloride transport	15/917	111/18,800	0.000319	0.006548	0.005629	*ABCB1/GABRB3/ANO5/ANO3/SLC26A4/SLC4A10/GLRA3/GABRA4/CASR/GABRG2/CFTR/CLIC3/GLRA1/GABRA2/SLC26A9*	15
BP	GO:0010876	Lipid localization	39/917	446/18,800	0.00032	0.006548	0.005629	*SPX/PLA2G1B/FITM1/CEL/ACACB/PNLIP/ABCB1/ABCA3/CLU/CD36/XKR4/LPA/ABCD2/TTPA/ANO3/FASLG/SLCO1C1/GALR1/GHRL/APOH/ABCA8/SLC22A9/NR0B2/AVPR1B/XKR7/SLC5A8/SLC10A2/RBP2/CYP4A11/CFTR/ADIPOQ/VSTM2A/PMP2/FABP4/MTTP/PLA2G2D/SERPINA5/CIDEA/CRP*	39
BP	GO:2000300	Regulation of synaptic vesicle exocytosis	10/917	56/18,800	0.000335	0.006822	0.005865	*CHRNB3/P2RX1/NLGN1/ADCY1/CACNB2/SYT4/LRRK2/SYP/CNR1/PRKCB*	10
BP	GO:0051962	Positive regulation of nervous system development	27/917	270/18,800	0.000338	0.006863	0.005901	*NTRK2/AMIGO1/NLGN1/CX3CR1/PAK3/RELN/ADGRB3/FLRT2/NRXN1/NEURL1/LRRTM2/KIT/GHRL/SLITRK1/ASPA/MAPT/NKX6-1/SOX11/DSCAM/CBLN1/CXCL12/LPAR3/ITPKA/ASCL1/NKX2-2/CAMK2B/LRRTM3*	27
BP	GO:0046683	Response to organophosphorus	16/917	124/18,800	0.000349	0.007055	0.006066	*AQP8/P2RX1/AKAP6/CDO1/P2RY12/PDE3A/SLC6A4/SLC8A1/CRHBP/ABCC9/KCNJ11/CNGA3/HCN4/CFTR/ADIPOQ/PCK1*	16
BP	GO:0009063	Cellular amino acid catabolic process	15/917	112/18,800	0.000353	0.007095	0.0061	*GLS2/TDH/NOS1/ABAT/CDO1/HOGA1/PAH/HPD/ASPA/HMGCLL1/MAT1A/PRODH2/OTC/CBS/GAD2*	15
BP	GO:0003015	Heart process	26/917	257/18,800	0.000361	0.007237	0.006222	*SPX/MYH7/KCNJ5/MYLK2/NOS1/FGF12/MYH6/SLC8A3/SLC8A2/DMD/CACNB2/SLC8A1/KCND3/ATP2B2/ABCC9/ANK2/SCN3B/ATP1A3/KCNA5/KCNJ3/CHRM2/HCN4/AGTR2/KCNH6/GLP1R/CHGA*	26
BP	GO:0048638	Regulation of developmental growth	31/917	329/18,800	0.000365	0.007281	0.00626	*ERBB4/NRCAM/ACACB/PROX1/AKAP6/MAP2/MYH6/TGFBR3/GAMT/SEMA6D/KIAA0319/SLC6A4/TNR/OLFM1/FSTL4/GHRL/BCL11A/MUSK/SYT4/MAPT/SEMA3E/UNC13A/NKX6-1/DSCAM/CXCL12/RIMS2/NPY1R/RIMS1/LPAR3/IGF1/ARX*	31

 © 2023. The Author(s).

Supplementary Table S1 (*Continued*)

Ontology	ID	Description	GeneRatio	BgRatio	p-Value	*p*.adjust	q-Value	geneID	Count
BP	GO:0031279	Regulation of cyclase activity	12/917	78/18,800	0.000375	0.007454	0.006409	*GUCA1C/VIPR2/NOS1/ADGRV1/GALR1/TSHR/ADCYAP1/P2RY13/CCR2/GLP1R/GUCA2A/GPR87*	12
BP	GO:0042136	Neurotransmitter biosynthetic process	5/917	14/18,800	0.000378	0.007475	0.006427	*NOS1/SLC5A7/PAH/CHAT/GAD2*	5
BP	GO:0098661	Inorganic anion transmembrane transport	13/917	90/18,800	0.000409	0.008064	0.006933	*GABRB3/ANO5/ANO3/SLC26A4/SLC17A1/GLRA3/GABRA4/CASR/GABRG2/CFTR/GLRA1/GABRA2/SLC26A9*	13
BP	GO:0007617	Mating behavior	8/917	38/18,800	0.000412	0.008072	0.00694	*P2RX1/MTNR1A/VIP/ABAT/TACR1/SLC6A4/MAPK8IP2/CNR1*	8
BP	GO:0030900	Forebrain development	34/917	376/18,800	0.000415	0.008107	0.00697	*SLC1A2/ERBB4/CCDC141/NTRK2/PROX1/BNIP3/RTN4RL1/RELN/P2RY12/SLC8A1/TNR/FOXP2/CHRNB2/SLC4A10/GDF7/LRRK2/CNTN2/PCSK1/NEUROD1/APLP1/ELAVL4/EOMES/SSTR2/CXCL12/FEZF1/SSTR3/ASCL1/PITX1/EPHA5/FGF10/NPY/CDK5R2/ARX/PAX5*	34
BP	GO:0071695	Anatomical structure maturation	25/917	246/18,800	0.000434	0.008447	0.007262	*BHLHA15/NRCAM/CX3CR1/ZBTB16/EPO/ADGRB3/PDE3A/RET/BCL11A/LRRK2/NKX6-1/CNTN2/SPINK1/MYOC/ASCL1/CFTR/IGF1/ACTL6B/TDRD1/CCL21/CDK5R2/FEV/PAEP/FGG/CCL19*	25
BP	GO:0006941	Striated muscle contraction	20/917	178/18,800	0.000443	0.008582	0.007378	*MYH7/KCNJ5/DTNA/MYLK2/NOS1/FGF12/MYH6/SLC8A3/DMD/CACNB2/SLC8A1/SCN4A/KCND3/ANK2/SCN3B/KLHL41/KCNA5/KCNJ3/HCN4/CHGA*	20
BP	GO:0030195	Negative regulation of blood coagulation	9/917	48/18,800	0.000453	0.008728	0.007504	*F11/ABAT/KLKB1/VTN/APOH/FGA/FGB/KRT1/FGG*	9
BP	GO:0051963	Regulation of synapse assembly	14/917	103/18,800	0.000471	0.009046	0.007777	*NTRK2/AMIGO1/NLGN1/ADGRB3/FLRT2/NRXN1/LRRTM2/CHRNB2/GHRL/SLITRK1/MUSK/CBLN1/LHFPL4/LRRTM3*	14
BP	GO:0007411	Axon guidance	24/917	234/18,800	0.000491	0.009387	0.008071	*NRCAM/RELN/SEMA6D/NFASC/DSCAML1/NCAM1/FLRT2/NRXN1/TNR/RET/GDF7/SEMA3E/CNTN2/KIF5C/DSCAM/DPYSL5/CXCL12/GFRA3/FEZF1/EPHA5/CDK5R2/CNTN5/ARX/UNC5D*	24
BP	GO:0002691	Regulation of cellular extravasation	8/917	39/18,800	0.000496	0.009407	0.008088	*PDGFD/SELP/CCR2/ITGA4/SELE/CXCL12/CCL21/FUT9*	8
BP	GO:0090278	Negative regulation of peptide hormone secretion	8/917	39/18,800	0.000496	0.009407	0.008088	*CARTPT/KCNB1/GHRL/CRHBP/SFRP1/KCNJ11/ABCC8/CHGA*	8
BP	GO:0007620	Copulation	6/917	22/18,800	0.000504	0.009521	0.008186	*P2RX1/VIP/ABAT/TACR1/SLC6A4/CNR1*	6

(*Continued*)

 © 2023. The Author(s).

Supplementary Table S1 (*Continued*)

Ontology	ID	Description	GeneRatio	BgRatio	p-Value	*p*.adjust	q-Value	geneID	Count
BP	GO:0097485	Neuron projection guidance	24/917	235/18,800	0.000522	0.009819	0.008442	*NRCAM/RELN/SEMA6D/NFASC/DSCAML1/NCAM1/FLRT2/NRXN1/TNR/RET/GDF7/SEMA3E/CNTN2/KIF5C/DSCAM/DPYSL5/CXCL12/GFRA3/FEZF1/EPHA5/CDK5R2/CNTN5/ARX/UNC5D*	24
BP	GO:0097553	Calcium ion transmembrane import into cytosol	18/917	154/18,800	0.000524	0.009819	0.008442	*P2RX1/NOS1/AKAP6/SLC8A3/SLC8A2/DMD/SLC8A1/FASLG/ANK2/MCOLN3/CACNA1B/CCR5/PTPRC/TRPV6/XCR1/CCL21/CXCL9/CCL19*	18
BP	GO:1900047	Negative regulation of hemostasis	9/917	49/18,800	0.000531	0.009905	0.008516	*F11/ABAT/KLKB1/VTN/APOH/FGA/FGB/KRT1/FGG*	9
BP	GO:0051966	Regulation of synaptic transmission, glutamatergic	11/917	70/18,800	0.000541	0.009996	0.008594	*NLGN1/RELN/NRXN1/TNR/UNC13A/CCR2/LRRK2/MAPK8IP2/CACNG5/CNR1/GRM4*	11
BP	GO:0036376	Sodium ion export across plasma membrane	5/917	15/18,800	0.000544	0.009996	0.008594	*FXYD2/SLC8A1/SLC4A4/ATP1A3/ATP4A*	5
BP	GO:0048172	Regulation of short-term neuronal synaptic plasticity	5/917	15/18,800	0.000544	0.009996	0.008594	*SLC8A2/SLC4A10/SYT4/SHISA9/SYP*	5
BP	GO:0099509	Regulation of presynaptic cytosolic calcium ion concentration	5/917	15/18,800	0.000544	0.009996	0.008594	*P2RX1/CACNB2/ATP2B2/SCGN/CNR1*	5
BP	GO:0046942	Carboxylic acid transport	27/917	279/18,800	0.000565	0.010322	0.008874	*SLC1A2/PLA2G1B/NTRK2/SLC16A12/SLC7A2/CD36/ABAT/SLC38A3/SLCO1C1/SLC26A4/NPY5R/CLTRN/SLC17A4/SYT4/SLC6A15/SLC17A1/SLC22A9/SLC3A1/NR0B2/AVPR1B/SLC5A8/SLC10A2/CASR/CYP4A11/SLC6A17/PLA2G2D/SLC26A9*	27
BP	GO:0007189	Adenylate cyclase-activating G protein-coupled receptor signaling pathway	18/917	155/18,800	0.000567	0.010322	0.008874	*GPHA2/VIP/ADCY1/ADGRB3/PTGFR/GALR1/TSHR/ADCYAP1/ADGRG2/APLP1/CNR1/GPR119/LPAR3/GLP1R/CHGA/CXCL9/UCN3/IAPP*	18
BP	GO:0002792	Negative regulation of peptide secretion	8/917	40/18,800	0.000594	0.010776	0.009265	*CARTPT/KCNB1/GHRL/CRHBP/SFRP1/KCNJ11/ABCC8/CHGA*	8
BP	GO:0019933	Camp-mediated signaling	9/917	50/18,800	0.00062	0.011116	0.009557	*PEX5L/AKAP6/ADCY1/PDE3A/PDE3B/ADCYAP1/GLP1R/EPHA5/SCTR*	9
BP	GO:0048168	Regulation of neuronal synaptic plasticity	9/917	50/18,800	0.00062	0.011116	0.009557	*SLC8A2/NEURL1/KIT/SLC4A10/SYT4/CNTN2/SHISA9/SYP/CAMK2B*	9
BP	GO:0061178	Regulation of insulin secretion involved in cellular response to glucose stimulus	9/917	50/18,800	0.00062	0.011116	0.009557	*BRSK2/GPLD1/GHRL/GPR27/RFX6/ZBED6/EPHA5/CFTR/NR1H4*	9

 © 2023. The Author(s).

Supplementary Table S1 (*Continued*)

Ontology	ID	Description	GeneRatio	BgRatio	p-Value	*p*.adjust	q-Value	geneID	Count
BP	GO:0007612	Learning	17/917	145/18,800	0.000715	0.012772	0.010981	*PRKAR2B/NTRK2/PAK5/RELN/ADGRB3/SLC8A3/SLC8A2/TACR1/NRXN1/TNR/KIT/CHRNB2/CNTN2/MAPK8IP2/ELAVL4/SNAP25/ABCC8*	17
BP	GO:0007157	Heterophilic cell-cell adhesion via plasma membrane cell adhesion molecules	9/917	51/18,800	0.00072	0.012824	0.011025	*UMOD/AMIGO1/NLGN1/REG3A/SELP/CBLN1/SELE/CADM3/NECTIN4*	9
BP	GO:0035883	Enteroendocrine cell differentiation	7/917	32/18,800	0.00074	0.013117	0.011277	*NKX6-1/NEUROD1/INSM1/RFX6/ZBED6/NKX2-2/BMP5*	7
BP	GO:1990138	Neuron projection extension	19/917	172/18,800	0.000751	0.013268	0.011407	*NRCAM/MAP2/SEMA6D/KIAA0319/TNR/OLFM1/BCL11A/SYT4/MAPT/SEMA3E/UNC13A/SPAG6/NKX6-1/DSCAM/ITGA4/CXCL12/RIMS2/RIMS1/SH3GL2*	19
BP	GO:0061041	Regulation of wound healing	16/917	133/18,800	0.000765	0.013448	0.011562	*F11/REG3G/CD36/ABAT/MMRN1/REG3A/KLKB1/VTN/AJAP1/APOH/ABCC8/FGA/FGB/KRT1/APCS/FGG*	16
BP	GO:0070588	Calcium ion transmembrane transport	29/917	314/18,800	0.000767	0.013448	0.011562	*BHLHA15/P2RX1/NOS1/AKAP6/TRPM6/RGN/SLC8A3/SLC8A2/DMD/CACNB2/SLC8A1/FASLG/ATP2B2/ANK2/MCOLN3/CACNA2D2/TRPM3/CACNA1B/CACNG5/CCR5/PTPRC/GPM6A/TRPV6/XCR1/CCL21/MRLN/CXCL9/PLP1/CCL19*	29
BP	GO:0036465	Synaptic vesicle recycling	11/917	73/18,800	0.000779	0.013554	0.011654	*CEL/NLGN1/DNAJC6/SH3GL3/PACSIN1/LRRK2/SYP/SNAP91/SH3GL2/SYT5/SYT8*	11
BP	GO:0140029	Exocytic process	11/917	73/18,800	0.000779	0.013554	0.011654	*P2RX1/NLGN1/RAB3C/KCNB1/GRIK5/UNC13A/SYT9/SNAP25/CPLX2/CFTR/TRARG1*	11
BP	GO:0014074	Response to purine-containing compound	16/917	134/18,800	0.000831	0.014335	0.012325	*AQP8/P2RX1/AKAP6/CDO1/P2RY12/PDE3A/SLC6A4/SLC8A1/CRHBP/ABCC9/KCNJ11/CNGA3/HCN4/CFTR/ADIPOQ/PCK1*	16
BP	GO:0006869	Lipid transport	34/917	391/18,800	0.000831	0.014335	0.012325	*SPX/PLA2G1B/CEL/PNLIP/ABCB1/ABCA3/CLU/CD36/XKR4/LPA/ABCD2/TTPA/ANO3/FASLG/SLCO1C1/GALR1/GHRL/APOH/ABCA8/SLC22A9/NR0B2/AVPR1B/XKR7/SLC5A8/SLC10A2/RBP2/CYP4A11/CFTR/ADIPOQ/PMP2/FABP4/MTTP/PLA2G2D/SERPINA5*	34
BP	GO:0050819	Negative regulation of coagulation	9/917	52/18,800	0.000834	0.014335	0.012325	*F11/ABAT/KLKB1/VTN/APOH/FGA/FGB/KRT1/FGG*	9
BP	GO:0007274	Neuromuscular synaptic transmission	6/917	24/18,800	0.000836	0.014335	0.012325	*DTNA/MYLK2/SLC5A7/CHRNB2/CHAT/RIMBP2*	6

(*Continued*)

 © 2023. The Author(s).

Global Medical Genetics © 2023. The Author(s).

Supplementary Table S1 (*Continued*)

Ontology	ID	Description	GeneRatio	BgRatio	p-Value	*p*.adjust	q-Value	geneID	Count
BP	GO:0016485	Protein processing	24/917	243/18,800	0.000841	0.014362	0.012347	*CUZD1/MBOAT4/F11/CMA1/KLKB1/TLL1/KLK1/CTSG/APOH/LRRK2/CNTN2/HPN/PCSK1/CPE/PCSK2/SCG5/FGA/KLK13/PRSS3/FGB/SLC30A8/TMPRSS4/KLK2/FGG*	24
BP	GO:0007613	Memory	15/917	122/18,800	0.000881	0.014985	0.012883	*CX3CR1/PAK5/RELN/ADCY1/SLC8A3/SLC8A2/TACR1/SLC6A4/CHRNB2/MUSK/SYT4/MAPT/CNR1/ABCC8/KLK8*	15
BP	GO:0045907	Positive regulation of vasoconstriction	7/917	33/18,800	0.0009	0.015198	0.013067	*TACR1/CYSLTR1/AVPR1B/CASR/FGA/FGB/FGG*	7
BP	GO:1902656	Calcium ion import into cytosol	7/917	33/18,800	0.0009	0.015198	0.013067	*P2RX1/SLC8A3/SLC8A2/SLC8A1/CACNA1B/TRPV6/MS4A1*	7
BP	GO:0046928	Regulation of neurotransmitter secretion	12/917	86/18,800	0.000925	0.015571	0.013388	*CHRNB3/P2RX1/NLGN1/ADCY1/CACNB2/SYT4/LRRK2/SYP/CNR1/RIMS1/PRKCB/CPLX2*	12
BP	GO:0050773	Regulation of dendrite development	13/917	98/18,800	0.000936	0.015695	0.013494	*PAK3/RELN/KIAA0319/ADGRB3/PACSIN1/CHRNB2/BCL11A/ELAVL4/CSMD3/ITPKA/SEZ6/CAMK2B/BMP5*	13
BP	GO:0010043	Response to zinc ion	9/917	53/18,800	0.000962	0.016028	0.01378	*KCNK3/MT1F/SLC30A2/MT1G/OTC/MT1H/ABCC8/GLRA1/SLC30A8*	9
BP	GO:1901605	Alpha-amino acid metabolic process	21/917	203/18,800	0.000963	0.016028	0.01378	*GNMT/GLS2/TDH/NOS1/CDO1/HOGA1/PAH/HPD/NAT8L/PSAT1/ASPA/DPEP1/HMGCLL1/MAT1A/PRODH2/ART4/OTC/CBS/GAD2/ATCAY/NR1H4*	21
BP	GO:0009410	Response to xenobiotic stimulus	35/917	411/18,800	0.001015	0.016787	0.014433	*AOX1/SLC1A2/KCNK3/ACACB/SCNN1B/ABCB1/ABCA3/GSTA2/GPLD1/NOS1/ABAT/VAV3/ADCY1/PDE3A/SLC6A4/SLC8A1/SULT1C4/RET/PPM1E/CRHBP/SFRP1/DPEP1/NKX6-1/KCNJ11/PCSK1/UGT2B15/NEUROD1/UGT2B11/CYP2C8/OTC/ABCC8/GAD2/ADIPOQ/CA9/SST*	35
BP	GO:0099173	Postsynapse organization	18/917	163/18,800	0.001026	0.016787	0.014433	*NRCAM/CEL/CTNND2/NLGN1/PAK3/RELN/KIF1A/CNKSR2/NRXN1/LRRTM2/GHRL/MUSK/LRRK2/INA/CBLN1/ITPKA/CAMK2B/LHFPL4*	18
BP	GO:0019233	Sensory perception of pain	13/917	99/18,800	0.00103	0.016787	0.014433	*SPX/MMP24/KCNA2/VIP/TACR1/PHF24/CHRNB2/CCR2/SCN3B/CXCL12/CNR1/NPY1R/IAPP*	13
BP	GO:0051588	Regulation of neurotransmitter transport	13/917	99/18,800	0.00103	0.016787	0.014433	*CHRNB3/P2RX1/NLGN1/NOS1/ADCY1/CACNB2/SYT4/LRRK2/SYP/CNR1/RIMS1/PRKCB/CPLX2*	13
BP	GO:0045956	Positive regulation of calcium ion-dependent exocytosis	5/917	17/18,800	0.001034	0.016787	0.014433	*KCNB1/SCAMP5/SYT4/SYT9/CDK5R2*	5

Supplementary Table S1 (*Continued*)

Ontology	ID	Description	GeneRatio	BgRatio	p-Value	*p*.adjust	q-Value	geneID	Count
BP	GO:0051969	Regulation of transmission of nerve impulse	5/917	17/18,800	0.001034	0.016787	0.014433	*FGF12/CARTPT/TNR/GHRL/GLRA1*	5
BP	GO:0072376	Protein activation cascade	5/917	17/18,800	0.001034	0.016787	0.014433	*KLKB1/APOH/FGA/FGB/FGG*	5
BP	GO:0021537	Telencephalon development	24/917	247/18,800	0.001056	0.017027	0.014639	*SLC1A2/ERBB4/CCDC141/NTRK2/PROX1/BNIP3/RTN4RL1/RELN/P2RY12/SLC8A1/TNR/FOXP2/LRRK2/CNTN2/NEUROD1/EOMES/CXCL12/FEZF1/ASCL1/EPHA5/NPY/CDK5R2/ARX/PAX5*	24
BP	GO:0098656	Anion transmembrane transport	24/917	247/18,800	0.001056	0.017027	0.014639	*SLC1A2/ABCB1/SLC16A12/SLC7A2/GABRB3/ANO5/ANO3/SLC38A3/SLC26A4/SLC4A4/CLTRN/SLC4A10/SLC17A1/GLRA3/SLC22A9/SLC3A1/SLC5A8/GABRA4/CASR/GABRG2/CFTR/GLRA1/GABRA2/SLC26A9*	24
BP	GO:0071248	Cellular response to metal ion	20/917	191/18,800	0.001083	0.017331	0.014901	*KCNK3/MT1F/SYT6/NLGN1/GPLD1/BNIP3/ADGRV1/ADCY1/MT1G/DLG2/SYT4/CRHBP/DPEP1/LRRK2/SYT9/MT1H/SYT5/GLRA1/FABP4/SYT8*	20
BP	GO:0035590	Purinergic nucleotide receptor signaling pathway	7/917	34/18,800	0.001086	0.017331	0.014901	*P2RX1/P2RY12/CTSG/P2RY13/GPR34/CNTN2/GPR87*	7
BP	GO:0086019	Cell-cell signaling involved in cardiac conduction	7/917	34/18,800	0.001086	0.017331	0.014901	*KCNJ5/CACNB2/ANK2/SCN3B/KCNA5/KCNJ3/HCN4*	7
BP	GO:0002067	Glandular epithelial cell differentiation	9/917	54/18,800	0.001105	0.017577	0.015112	*PROX1/NKX6-1/NEUROD1/INSM1/RFX6/ZBED6/ASCL1/NKX2-2/BMP5*	9
BP	GO:0060560	Developmental growth involved in morphogenesis	23/917	234/18,800	0.001139	0.018029	0.015501	*NRCAM/MAP2/SEMA6D/KIAA0319/TNR/OLFM1/FSTL4/BCL11A/SYT4/MAPT/SFRP1/SEMA3E/UNC13A/SPAG6/NKX6-1/DSCAM/ITGA4/CXCL12/RIMS2/RIMS1/SH3GL2/LPAR3/FGF10*	23
BP	GO:0060048	Cardiac muscle contraction	16/917	138/18,800	0.001141	0.018029	0.015501	*MYH7/KCNJ5/MYLK2/NOS1/FGF12/MYH6/DMD/CACNB2/SLC8A1/KCND3/ANK2/SCN3B/KCNA5/KCNJ3/HCN4/CHGA*	16
BP	GO:0019098	Reproductive behavior	8/917	44/18,800	0.001152	0.018132	0.015589	*P2RX1/MTNR1A/VIP/ABAT/TACR1/SLC6A4/MAPK8IP2/CNR1*	8
BP	GO:0002526	Acute inflammatory response	14/917	113/18,800	0.001198	0.018743	0.016114	*REG3G/KL/EPO/TACR1/REG3A/KLKB1/NPY5R/C2CD4B/DNASE1L3/CNR1/NPY/PLA2G2D/APCS/CRP*	14

(*Continued*)

 © 2023. The Author(s).

Global Medical Genetics © 2023. The Author(s).

Supplementary Table S1 (*Continued*)

Ontology	ID	Description	GeneRatio	BgRatio	p-Value	*p*.adjust	q-Value	geneID	Count
BP	GO:0050900	Leukocyte migration	33/917	384/18,800	0.001199	0.018743	0.016114	*C5/PLA2G1B/UMOD/PDGFD/GP2/CX3CR1/VAV3/TACR1/P2RY12/SELP/RET/KIT/CTSG/CCR2/CCL14/ITGA4/VEGFD/MADCAM1/SELE/CCR5/CXCL12/GPR18/SCG2/THBS4/GPR15/CHGA/BMP5/CCL21/CXCR5/CXCL9/S100A14/FUT9/CCL19*	33
BP	GO:0086001	Cardiac muscle cell action potential	11/917	77/18,800	0.001226	0.019102	0.016423	*KCNJ5/FGF12/DMD/CACNB2/SLC8A1/KCND3/ANK2/SCN3B/KCNA5/KCNJ3/HCN4*	11
BP	GO:0048813	Dendrite morphogenesis	16/917	139/18,800	0.001233	0.019148	0.016462	*CTNND2/NLGN1/PAK3/MAP2/RELN/KIF1A/ADGRB3/CHRNB2/DCDC2/LRRK2/MAPK8IP2/DSCAM/ELAVL4/CTNNA2/ITPKA/CAMK2B*	16
BP	GO:0090257	Regulation of muscle system process	24/917	250/18,800	0.001247	0.019299	0.016592	*SPX/MYH7/P2RX1/MYLK2/NOS1/AKAP6/ABAT/CHRM3/SLC8A3/TACR1/DMD/SLC8A1/SCN4A/KIT/GHRL/ANK2/SSTR2/CHRM2/HCN4/KCNB2/ABCC8/IGF1/CAMK2B/CHGA*	24
BP	GO:0070098	Chemokine-mediated signaling pathway	12/917	89/18,800	0.001257	0.019394	0.016674	*CX3CR1/CCR2/CCL14/CXCR6/CCR5/CXCL12/ACKR1/XCR1/CCL21/CXCR5/CXCL9/CCL19*	12
BP	GO:0060998	Regulation of dendritic spine development	9/917	55/18,800	0.001265	0.019453	0.016725	*NLGN1/PAK3/RELN/KIF1A/SDK1/NEURL1/FSTL4/ITPKA/CAMK2B*	9
BP	GO:0030193	Regulation of blood coagulation	10/917	66/18,800	0.001278	0.019581	0.016835	*F11/CD36/ABAT/KLKB1/VTN/APOH/FGA/FGB/KRT1/FGG*	10
BP	GO:0019228	Neuronal action potential	7/917	35/18,800	0.001301	0.019873	0.017086	*P2RX1/KCNA2/FGF12/SCN3A/SCN4A/SCN7A/KCNMB2*	7
BP	GO:0010959	Regulation of metal ion transport	34/917	403/18,800	0.001392	0.020961	0.018021	*PLA2G1B/P2RX1/AMIGO1/KCNAB1/NOS1/AKAP6/FGF12/WNK2/RGN/VIP/DPP10/EPO/FXYD2/DMD/CACNB2/HOMER2/SLC8A1/EGF/ANK2/SCN3B/SPINK1/MIR210/DPP6/KCNA5/CXCL12/CASR/ABCC8/GCK/CAMK2B/PLPP4/MRLN/CXCL9/PLP1/FFAR1*	34
BP	GO:0050770	Regulation of axonogenesis	17/917	154/18,800	0.001404	0.020961	0.018021	*BRSK2/NTRK2/AMIGO1/PAK3/MAP2/SEMA6D/KIAA0319/TNR/RET/FSTL4/SLITRK1/MAPT/SEMA3E/CNTN2/DSCAM/CXCL12/LPAR3*	17
BP	GO:0051610	Serotonin uptake	4/917	11/18,800	0.001409	0.020961	0.018021	*NOS1/SLC6A4/SLC29A4/SLC18A2*	4
BP	GO:0086070	SA node cell to atrial cardiac muscle cell communication	4/917	11/18,800	0.001409	0.020961	0.018021	*ANK2/SCN3B/KCNA5/HCN4*	4
BP	GO:0097048	Dendritic cell apoptotic process	4/917	11/18,800	0.001409	0.020961	0.018021	*CXCL12/CCL21/BLK/CCL19*	4
BP	GO:0099171	Presynaptic modulation of chemical synaptic transmission	4/917	11/18,800	0.001409	0.020961	0.018021	*CACNB2/SYT4/CNR1/PRKCB*	4

Supplementary Table S1 (Continued)

Ontology	ID	Description	GeneRatio	BgRatio	p-Value	*p*.adjust	q-Value	geneID	Count
BP	GO:1903238	Positive regulation of leukocyte tethering or rolling	4/917	11/18,800	0.001409	0.020961	0.018021	*SELP/CCR2/ITGA4/SELE*	4
BP	GO:2000668	Regulation of dendritic cell apoptotic process	4/917	11/18,800	0.001409	0.020961	0.018021	*CXCL12/CCL21/BLK/CCL19*	4
BP	GO:0046395	Carboxylic acid catabolic process	23/917	238/18,800	0.001428	0.02114	0.018175	*GLS2/TDH/CEL/ACACB/NOS1/ABAT/CDO1/ACADL/HOGA1/ABCD2/PAH/HPD/ASPA/HMGCLL1/MAT1A/PRODH2/OTC/CNR1/CBS/GAD2/CYP4A11/ADIPOQ/PCK1*	23
BP	GO:0035418	Protein localization to synapse	10/917	67/18,800	0.001438	0.02114	0.018175	*NPHS1/NLGN1/RELN/BSN/NRXN1/PCLO/DLG2/MAPT/KIF5C/SNAP25*	10
BP	GO:0043113	Receptor clustering	9/917	56/18,800	0.001443	0.02114	0.018175	*NLGN1/RELN/NRXN1/DLG2/GRIK5/MUSK/ITGA4/MADCAM1/LHFPL4*	9
BP	GO:0055078	Sodium ion homeostasis	9/917	56/18,800	0.001443	0.02114	0.018175	*SPX/SCNN1B/TACR1/SLC8A1/SCNN1G/SCN7A/ATP1A3/ATP4A/CYP4A11*	9
BP	GO:0061756	Leukocyte adhesion to vascular endothelial cell	9/917	56/18,800	0.001443	0.02114	0.018175	*CX3CR1/SELP/CCR2/ITGA4/MADCAM1/SELE/CXCL12/CCL21/FUT9*	9
BP	GO:0060284	Regulation of cell development	40/917	500/18,800	0.001518	0.022157	0.019049	*NTRK2/AMIGO1/CX3CR1/PAK3/MAP2/RELN/SEMA6D/KIAA0319/TTPA/PDE3A/TNR/RET/NEURL1/KIT/FSTL4/SLITRK1/BCL11A/ASPA/SYT4/MAPT/SEMA3E/LRRK2/NKX6-1/SOX11/DSCAM/CXCL12/LPAR3/MYOC/FEZF1/ITPKA/ABCC8/ASCL1/FGA/NKX2-2/IGF1/ADIPOQ/CAMK2B/FGB/PAEP/FGG*	40
BP	GO:0031018	Endocrine pancreas development	8/917	46/18,800	0.001556	0.022647	0.019471	*NKX6-1/NEUROD1/INSM1/ONECUT1/RFX6/ZBED6/NKX2-2/BMP5*	8
BP	GO:1900046	Regulation of hemostasis	10/917	68/18,800	0.001613	0.023402	0.02012	*F11/CD36/ABAT/KLKB1/VTN/APOH/FGA/FGB/KRT1/FGG*	10
BP	GO:0035774	Positive regulation of insulin secretion involved in cellular response to glucose stimulus	6/917	27/18,800	0.001623	0.023402	0.02012	*GPLD1/GHRL/GPR27/RFX6/CFTR/NR1H4*	6
BP	GO:0051350	Negative regulation of lyase activity	6/917	27/18,800	0.001623	0.023402	0.02012	*ADGRV1/GALR1/P2RY13/CCR2/MIR210/GPR87*	6
BP	GO:0098900	Regulation of action potential	9/917	57/18,800	0.001641	0.023589	0.020281	*FGF12/CD36/SLC8A2/TACR1/KCNB1/CHRNB2/ANK2/HCN4/CNR1*	9

(Continued)

Global Medical Genetics © 2023. The Author(s).

Supplementary Table S1 (*Continued*)

Ontology	ID	Description	GeneRatio	BgRatio	p-Value	*p*.adjust	q-Value	geneID	Count
BP	GO:0002065	Columnar/cuboidal epithelial cell differentiation	12/917	92/18,800	0.001682	0.024101	0.020721	*PROX1/ENAM/NKX6-1/SOX11/NEUROD1/INSM1/RFX6/ZBED6/ASCL1/NKX2-2/NPY/BMP5*	12
BP	GO:0016054	Organic acid catabolic process	23/917	242/18,800	0.001777	0.025338	0.021785	*GLS2/TDH/CEL/ACACB/NOS1/ABAT/CDO1/ACADL/HOGA1/ABCD2/PAH/HPD/ASPA/HMGCLL1/MAT1A/PRODH2/OTC/CNR1/CBS/GAD2/CYP4A11/ADIPOQ/PCK1*	23
BP	GO:1901890	Positive regulation of cell junction assembly	13/917	105/18,800	0.001779	0.025338	0.021785	*NTRK2/AMIGO1/NLGN1/ADGRB3/FLRT2/NRXN1/LRRTM2/GHRL/SLITRK1/SFRP1/CBLN1/MYOC/LRRTM3*	13
BP	GO:0002673	Regulation of acute inflammatory response	8/917	47/18,800	0.001797	0.025427	0.021861	*KLKB1/NPY5R/C2CD4B/DNASE1L3/CNR1/NPY/PLA2G2D/APCS*	8
BP	GO:0042220	Response to cocaine	8/917	47/18,800	0.001797	0.025427	0.021861	*SLC1A2/ABAT/SDK1/HOMER2/CHRNB2/CRHBP/ELAVL4/CNR1*	8
BP	GO:0032094	Response to food	7/917	37/18,800	0.00183	0.025824	0.022202	*SPX/SCNN1B/CLPS/CARTPT/GHRL/NPY/CLPSL1*	7
BP	GO:0022029	Telencephalon cell migration	9/917	58/18,800	0.00186	0.026009	0.022362	*CCDC141/RELN/P2RY12/TNR/LRRK2/CNTN2/CXCL12/CDK5R2/ARX*	9
BP	GO:0045761	Regulation of adenylate cyclase activity	9/917	58/18,800	0.00186	0.026009	0.022362	*VIPR2/ADGRV1/GALR1/TSHR/ADCYAP1/P2RY13/CCR2/GLP1R/GPR87*	9
BP	GO:0086065	Cell communication involved in cardiac conduction	9/917	58/18,800	0.00186	0.026009	0.022362	*KCNJ5/CACNB2/SLC8A1/ANK2/SCN3B/ATP1A3/KCNA5/KCNJ3/HCN4*	9
BP	GO:0007631	Feeding behavior	13/917	106/18,800	0.00194	0.027039	0.023247	*NTRK2/CARTPT/TACR1/NPY5R/GHRL/CNTN2/DACH1/PRLHR/CNR1/NPY1R/NPY/PPY/IAPP*	13
BP	GO:0051259	Protein complex oligomerization	23/917	244/18,800	0.001978	0.027134	0.023328	*GNMT/SLC1A2/ACACB/ABCA3/KCNA2/TRPM6/KCTD16/KCNB1/AQP4/KCNA3/KCND3/KCNV1/PEG10/MAT1A/TRPM3/GLRA3/KCNA5/SCARA5/KCNB2/KCNG3/KCTD8/KRT1/MS4A1*	23
BP	GO:0001835	Blastocyst hatching	6/917	28/18,800	0.001982	0.027134	0.023328	*AKAP3/ARHGDIG/PPP4R4/ST8SIA6/ZBED6/PLPP4*	6
BP	GO:0035188	Hatching	6/917	28/18,800	0.001982	0.027134	0.023328	*AKAP3/ARHGDIG/PPP4R4/ST8SIA6/ZBED6/PLPP4*	6
BP	GO:0042133	Neurotransmitter metabolic process	6/917	28/18,800	0.001982	0.027134	0.023328	*NOS1/ABAT/SLC5A7/PAH/CHAT/GAD2*	6
BP	GO:0071684	Organism emergence from protective structure	6/917	28/18,800	0.001982	0.027134	0.023328	*AKAP3/ARHGDIG/PPP4R4/ST8SIA6/ZBED6/PLPP4*	6

Supplementary Table S1 (*Continued*)

Ontology	ID	Description	GeneRatio	BgRatio	p-Value	*p*.adjust	q-Value	geneID	Count
BP	GO:0090330	Regulation of platelet aggregation	6/917	28/18,800	0.001982	0.027134	0.023328	*CELA2A/ABAT/MMRN1/CTSG/BLK/FGG*	6
BP	GO:0051260	Protein homooligomerization	19/917	187/18,800	0.002024	0.027413	0.023569	*GNMT/SLC1A2/ACACB/ABCA3/KCNA2/KCTD16/KCNB1/AQP4/KCNA3/KCND3/KCNV1/PEG10/MAT1A/GLRA3/KCNA5/SCARA5/KCNB2/KCNG3/KCTD8*	19
BP	GO:0048709	Oligodendrocyte differentiation	12/917	94/18,800	0.002026	0.027413	0.023569	*NTRK2/CLU/BNIP3/SOX6/SLC8A3/ASPA/NKX6-1/CNTN2/SOX11/ASCL1/NKX2-2/PLP1*	12
BP	GO:1905954	Positive regulation of lipid localization	12/917	94/18,800	0.002026	0.027413	0.023569	*FITM1/ACACB/ABCA3/CD36/FASLG/GALR1/GHRL/AVPR1B/CYP4A11/ADIPOQ/VSTM2A/CIDEA*	12
BP	GO:0072148	Epithelial cell fate commitment	4/917	12/18,800	0.002032	0.027413	0.023569	*PROX1/NEUROD1/NKX2-2/ARX*	4
BP	GO:1901142	Insulin metabolic process	4/917	12/18,800	0.002032	0.027413	0.023569	*CELA2A/CPE/PCSK2/SLC30A8*	4
BP	GO:0050805	Negative regulation of synaptic transmission	10/917	71/18,800	0.002247	0.03014	0.025913	*KCNB1/SLC6A4/TNR/NPY5R/CELF4/SYT4/MAPT/LRRK2/CBLN1/ADIPOQ*	10
BP	GO:0050818	Regulation of coagulation	10/917	71/18,800	0.002247	0.03014	0.025913	*F11/CD36/ABAT/KLKB1/VTN/APOH/FGA/FGB/KRT1/FGG*	10
BP	GO:0019935	Cyclic-nucleotide-mediated signaling	11/917	83/18,800	0.002273	0.030405	0.026141	*PEX5L/AKAP6/CD36/ADCY1/PDE3A/PDE3B/ADCYAP1/GLP1R/EPHA5/RUNDC3A/SCTR*	11
BP	GO:0061387	Regulation of extent of cell growth	13/917	108/18,800	0.002296	0.030625	0.02633	*NRCAM/MAP2/SEMA6D/KIAA0319/TNR/OLFM1/FSTL4/MAPT/SEMA3E/NKX6-1/DSCAM/CXCL12/LPAR3*	13
BP	GO:1903793	Positive regulation of anion transport	8/917	49/18,800	0.002365	0.031402	0.026998	*ABCB1/ABAT/SLC38A3/CLTRN/SYT4/AVPR1B/CYP4A11/CFTR*	8
BP	GO:00468,88	Negative regulation of hormone secretion	9/917	60/18,800	0.002368	0.031402	0.026998	*CARTPT/KCNB1/GHRL/CRHBP/SFRP1/KCNJ11/ABCC8/ADIPOQ/CHGA*	9
BP	GO:0003309	Type B pancreatic cell differentiation	6/917	29/18,800	0.002397	0.031425	0.027018	*NKX6-1/INSM1/RFX6/ZBED6/NKX2-2/BMP5*	6
BP	GO:0007271	Synaptic transmission, cholinergic	6/917	29/18,800	0.002397	0.031425	0.027018	*CHRNB3/CHRM3/TACR1/RIC3/SLC5A7/CHRNB2*	6
BP	GO:0015701	Bicarbonate transport	6/917	29/18,800	0.002397	0.031425	0.027018	*CA4/SLC26A4/SLC4A4/SLC4A10/CFTR/SLC26A9*	6
BP	GO:0051968	Positive regulation of synaptic transmission, glutamatergic	6/917	29/18,800	0.002397	0.031425	0.027018	*NLGN1/RELN/NRXN1/TNR/CCR2/CACNG5*	6

(*Continued*)

 © 2023. The Author(s).

Supplementary Table S1 (*Continued*)

Ontology	ID	Description	GeneRatio	BgRatio	p-Value	*p*.adjust	q-Value	geneID	Count
BP	GO:0051899	Membrane depolarization	11/917	84/18,800	0.002503	0.032632	0.028055	*SCN3A/CACNB2/SLC8A1/SCN4A/CHRNB2/ANK2/LRRK2/SCN3B/SCN7A/HCN4/MYOC*	11
BP	GO:0071277	Cellular response to calcium ion	11/917	84/18,800	0.002503	0.032632	0.028055	*SYT6/NLGN1/GPLD1/ADGRV1/ADCY1/SYT4/CRHBP/DPEP1/SYT9/SYT5/SYT8*	11
BP	GO:1904994	Regulation of leukocyte adhesion to vascular endothelial cell	7/917	39/18,800	0.002513	0.032663	0.028082	*SELP/CCR2/ITGA4/SELE/CXCL12/CCL21/FUT9*	7
BP	GO:1990868	Response to chemokine	12/917	97/18,800	0.002648	0.034233	0.029432	*CX3CR1/CCR2/CCL14/CXCR6/CCR5/CXCL12/ACKR1/XCR1/CCL21/CXCR5/CXCL9/CCL19*	12
BP	GO:1990869	Cellular response to chemokine	12/917	97/18,800	0.002648	0.034233	0.029432	*CX3CR1/CCR2/CCL14/CXCR6/CCR5/CXCL12/ACKR1/XCR1/CCL21/CXCR5/CXCL9/CCL19*	12
BP	GO:0021885	Forebrain cell migration	9/917	61/18,800	0.00266	0.034293	0.029483	*CCDC141/RELN/P2RY12/TNR/LRRK2/CNTN2/CXCL12/CDK5R2/ARX*	9
BP	GO:0099054	Presynapse assembly	8/917	50/18,800	0.002697	0.034578	0.029728	*CEL/NLGN1/BSN/PCLO/SLITRK1/CBLN1/CNTN5/LRRTM3*	8
BP	GO:1903307	Positive regulation of regulated secretory pathway	8/917	50/18,800	0.002697	0.034578	0.029728	*NLGN1/CACNB2/KCNB1/SCAMP5/SYT4/SYT9/CNR1/CDK5R2*	8
BP	GO:0043266	Regulation of potassium ion transport	13/917	110/18,800	0.002705	0.034579	0.02973	*AMIGO1/KCNAB1/NOS1/AKAP6/WNK2/VIP/DPP10/FXYD2/ANK2/DPP6/KCNA5/ABCC8/GCK*	13
BP	GO:0045123	Cellular extravasation	10/917	73/18,800	0.00277	0.035321	0.030367	*PDGFD/CX3CR1/SELP/CCR2/ITGA4/MADCAM1/SELE/CXCL12/CCL21/FUT9*	10
BP	GO:0033604	Negative regulation of catecholamine secretion	4/917	13/18,800	0.002822	0.035882	0.03085	*ABAT/SYT4/CNR1/CHGA*	4
BP	GO:0086011	Membrane repolarization during action potential	6/917	30/18,800	0.002874	0.03615	0.03108	*KCNJ5/KCNH7/KCND3/KCNA5/KCNJ3/KCNH6*	6
BP	GO:0120162	Positive regulation of cold-induced thermogenesis	12/917	98/18,800	0.002887	0.03615	0.03108	*ESRRG/GATM/CD36/ACADL/KSR2/PRLR/TSHR/GHRL/ADCYAP1/CCR2/ADIPOQ/FABP4*	12
BP	GO:0007194	Negative regulation of adenylate cyclase activity	5/917	21/18,800	0.00289	0.03615	0.03108	*ADGRV1/GALR1/P2RY13/CCR2/GPR87*	5
BP	GO:0032098	Regulation of appetite	5/917	21/18,800	0.00289	0.03615	0.03108	*SPX/CARTPT/GHRL/NPY/SCTR*	5
BP	GO:0044241	Lipid digestion	5/917	21/18,800	0.00289	0.03615	0.03108	*CEL/PNLIP/PNLIPRP2/CD36/ARX*	5

 © 2023. The Author(s).

Supplementary Table S1 (*Continued*)

Ontology	ID	Description	GeneRatio	BgRatio	p-Value	*p*.adjust	q-Value	geneID	Count
BP	GO:0099558	Maintenance of synapse structure	5/917	21/18,800	0.00289	0.03615	0.03108	*ADGRB3/BSN/PCLO/CBLN1/CBLN4*	5
BP	GO:0010720	Positive regulation of cell development	26/917	297/18,800	0.002949	0.036785	0.031626	*NTRK2/AMIGO1/CX3CR1/PAK3/RELN/PDE3A/RET/NEURL1/KIT/SLITRK1/ASPA/MAPT/NKX6-1/SOX11/DSCAM/CXCL12/LPAR3/MYOC/ITPKA/ASCL1/FGA/NKX2-2/ADIPOQ/CAMK2B/FGB/FGG*	26
BP	GO:0099175	Regulation of postsynapse organization	11/917	86/18,800	0.003019	0.037562	0.032294	*NRCAM/NLGN1/PAK3/RELN/KIF1A/LRRTM2/GHRL/LRRK2/CBLN1/ITPKA/CAMK2B*	11
BP	GO:0051932	Synaptic transmission, gabaergic	8/917	51/18,800	0.003065	0.038028	0.032695	*NLGN1/TACR1/NPY5R/PHF24/CNR1/GABRA4/GABRG2/GABRA2*	8
BP	GO:2001257	Regulation of cation channel activity	18/917	180/18,800	0.003114	0.038533	0.033129	*AMIGO1/NLGN1/KCNAB1/NOS1/AKAP6/RELN/FGF12/EPO/DMD/CACNB2/PIRT/CRHBP/ANK2/CCR2/SHISA9/MAPK8IP2/CACNG5/ABCC8*	18
BP	GO:0015908	Fatty acid transport	12/917	99/18,800	0.003143	0.03879	0.03335	*SPX/PLA2G1B/CD36/ABCD2/SLC22A9/AVPR1B/SLC5A8/RBP2/CYP4A11/PMP2/FABP4/PLA2G2D*	12
BP	GO:0006939	Smooth muscle contraction	13/917	112/18,800	0.003171	0.03903	0.033556	*SPX/SCNN1B/P2RX1/ABAT/CHRM3/TACR1/SLC8A1/KIT/CHRNB2/GHRL/SSTR2/CHRM2/KCNB2*	13
BP	GO:0045987	Positive regulation of smooth muscle contraction	6/917	31/18,800	0.00342	0.041937	0.036055	*SPX/ABAT/CHRM3/TACR1/KIT/GHRL*	6
BP	GO:0018958	Phenol-containing compound metabolic process	13/917	113/18,800	0.003427	0.041937	0.036055	*GCNT4/KL/RNF180/ABAT/GPR37/PAH/SLCO1C1/CHRNB2/HPN/TPO/INSM1/DUOXA1/NPY*	13
BP	GO:1901652	Response to peptide	38/917	491/18,800	0.003464	0.041937	0.036055	*PLA2G1B/CELA2A/GRB14/PDK4/GPLD1/REG3G/KL/CD36/KLF15/TGFBR3/REG1B/CDO1/REG3A/ECHDC3/TSHR/GHRL/PDE3B/REG1A/CRHBP/NKX6-1/SPINK1/PCSK1/ITGA4/CACNA1B/ATP1A3/CXCL12/OTC/PRKCB/AGTR2/CASR/ABCC8/GLP1R/GCK/NR1H4/IGF1/ADIPOQ/TRARG1/PCK1*	38
BP	GO:0007215	Glutamate receptor signaling pathway	8/917	52/18,800	0.003471	0.041937	0.036055	*KCNB1/HOMER2/FRRS1L/GRIK5/GRIA2/GRIA4/PLP1/GRM4*	8
BP	GO:0086009	Membrane repolarization	8/917	52/18,800	0.003471	0.041937	0.036055	*KCNJ5/AKAP6/KCNH7/KCND3/ANK2/KCNA5/KCNJ3/KCNH6*	8
BP	GO:0097479	Synaptic vesicle localization	8/917	52/18,800	0.003471	0.041937	0.036055	*BRSK2/NLGN1/MAP2/PCLO/LRRK2/KIF5C/SNAP91/AP3B2*	8

(*Continued*)

Global Medical Genetics © 2023. The Author(s).

Global Medical Genetics © 2023. The Author(s).

Supplementary Table S1 (*Continued*)

Ontology	ID	Description	GeneRatio	BgRatio	p-Value	*p*.adjust	q-Value	geneID	Count
BP	GO:0099172	Presynapse organization	8/917	52/18,800	0.003471	0.041937	0.036055	*CEL/NLGN1/BSN/PCLO/SLITRK1/CBLN1/CNTN5/LRRTM3*	8
BP	GO:0015698	Inorganic anion transport	18/917	182/18,800	0.003505	0.042247	0.036322	*ABCB1/GABRB3/ANO5/ANO3/SLC26A4/SLC4A4/SLC4A10/ABCC9/SLC17A1/GLRA3/GABRA4/CASR/GABRG2/CFTR/CLIC3/GLRA1/GABRA2/SLC26A9*	18
BP	GO:0006883	Cellular sodium ion homeostasis	5/917	22/18,800	0.003592	0.042844	0.036836	*SCNN1B/SLC8A1/SCNN1G/ATP1A3/ATP4A*	5
BP	GO:0086012	Membrane depolarization during cardiac muscle cell action potential	5/917	22/18,800	0.003592	0.042844	0.036836	*CACNB2/SLC8A1/ANK2/SCN3B/HCN4*	5
BP	GO:0099500	Vesicle fusion to plasma membrane	5/917	22/18,800	0.003592	0.042844	0.036836	*GRIK5/SYT9/SNAP25/CPLX2/TRARG1*	5
BP	GO:2000310	Regulation of NMDA receptor activity	5/917	22/18,800	0.003592	0.042844	0.036836	*NLGN1/RELN/CRHBP/CCR2/MAPK8IP2*	5
BP	GO:0043200	Response to amino acid	13/917	114/18,800	0.003701	0.044025	0.037851	*CASTOR3/SLC1A2/NTRK2/AMIGO1/PDGFD/CASTOR2/CDO1/NEURL1/BCL11A/GLRA3/GLRA1/PCK1/SST*	13
BP	GO:0098930	Axonal transport	9/917	64/18,800	0.003711	0.044032	0.037857	*SYBU/MAP2/KIF1A/DLG2/MAPT/KIF5C/AGBL4/AP3B2/CALY*	9
BP	GO:0021953	Central nervous system neuron differentiation	17/917	169/18,800	0.003765	0.044297	0.038084	*NTRK2/PROX1/MAP2/CHRNB2/SLC4A10/GDF7/MAPT/NKX6-1/CNTN2/ELAVL4/CBLN1/EOMES/AGBL4/ASCL1/NKX2-2/NPY/ARX*	17
BP	GO:0002864	Regulation of acute inflammatory response to antigenic stimulus	4/917	14/18,800	0.0038	0.044297	0.038084	*NPY5R/CNR1/NPY/PLA2G2D*	4
BP	GO:0034392	Negative regulation of smooth muscle cell apoptotic process	4/917	14/18,800	0.0038	0.044297	0.038084	*APOH/MIR210/GRIA4/IGF1*	4
BP	GO:0035589	G protein-coupled purinergic nucleotide receptor signaling pathway	4/917	14/18,800	0.0038	0.044297	0.038084	*P2RY12/P2RY13/GPR34/GPR87*	4
BP	GO:0048681	Negative regulation of axon regeneration	4/917	14/18,800	0.0038	0.044297	0.038084	*RTN4RL1/KIAA0319/TNR/KLK8*	4

Supplementary Table S1 (*Continued*)

Ontology	ID	Description	GeneRatio	BgRatio	p-Value	*p*.adjust	q-Value	geneID	Count
BP	GO:0050930	Induction of positive chemotaxis	4/917	14/18,800	0.0038	0.044297	0.038084	*VEGFD/CXCL12/SCG2/FGF10*	4
BP	GO:0090128	Regulation of synapse maturation	4/917	14/18,800	0.0038	0.044297	0.038084	*RELN/NRXN1/NEURL1/CAMK2B*	4
BP	GO:0014003	Oligodendrocyte development	7/917	42/18,800	0.003883	0.045151	0.038819	*CLU/ASPA/CNTN2/SOX11/ASCL1/NKX2-2/PLP1*	7
BP	GO:0007589	Body fluid secretion	11/917	89/18,800	0.003951	0.045824	0.039397	*ERBB4/CEL/SCNN1B/VIP/CDO1/CHRM3/TACR1/PRLR/NEURL1/SPINK1/FGF10*	11
BP	GO:0071711	Basement membrane organization	6/917	32/18,800	0.004038	0.046603	0.040067	*GAS2/CMA1/LAMB4/FLRT2/HPN/MMP11*	6
BP	GO:0098703	Calcium ion import across plasma membrane	6/917	32/18,800	0.004038	0.046603	0.040067	*P2RX1/SLC8A3/SLC8A2/SLC8A1/CACNA1B/TRPV6*	6
BP	GO:0006940	Regulation of smooth muscle contraction	9/917	65/18,800	0.004125	0.047488	0.040828	*SPX/P2RX1/ABAT/CHRM3/TACR1/KIT/GHRL/CHRM2/KCNB2*	9
BP	GO:0097529	Myeloid leukocyte migration	21/917	229/18,800	0.004186	0.048076	0.041333	*C5/PLA2G1B/UMOD/PDGFD/GP2/CX3CR1/VAV3/P2RY12/KIT/CTSG/CCR2/CCL14/VEGFD/CXCL12/SCG2/THBS4/CHGA/CCL21/CXCL9/S100A14/CCL19*	21
BP	GO:0070252	Actin-mediated cell contraction	12/917	103/18,800	0.004357	0.049915	0.042915	*MYH7/KCNJ5/MYLK2/FGF12/MYH6/CACNB2/KCND3/ANK2/SCN3B/KCNA5/KCNJ3/HCN4*	12
CC	GO:0019814	Immunoglobulin complex	51/967	167/19,594	7.67E-27	3.61E-24	2.76E-24	*IGKV2-28/IGKV2D-28/IGHV3-64/IGHV6-1/IGHV2-5/IGHV3-74/IGLV5-37/IGHV4-4/IGLV7-46/IGHV3-20/IGLV7-43/IGKV1D-39/IGKV1-12/IGHV3-7/IGHV3-15/IGKV1-39/IGHV1-45/JCHAIN/IGKV5-2/IGLV2-8/IGKV1D-13/IGHV3-72/IGLV2-18/IGKV1-17/IGKV2-24/IGHV3-48/IGHV2-26/IGHV3-49/IGHV4-28/IGKV1-6/IGLV4-60/IGLV6-57/IGKV1-16/IGLV4-69/IGLV10-54/IGHV5-51/IGLV3-27/IGLV5-45/IGKV4-1/IGHV1-69/IGLV3-19/IGHV4-39/IGKV3D-20/IGKV1-27/IGHV4-59/IGHA2/IGHV3-21/IGKV1-9/IGHV3-13/IGLV8-61/IGHV1-18*	51
CC	GO:0009897	External side of plasma membrane	73/967	455/19,594	2.88E-19	6.78E-17	5.19E-17	*NRCAM/SCNN1B/P2RX1/ABCB1/NLGN1/LIFR/GP2/CX3CR1/CA4/CD36/IL12RB2/TGFBR3/NCAM1/FASLG/AQP4/SELP/PRLR/KIT/CHRNB2/SCNN1G/IL5RA/ASTN1/ANPEP/CCR2/GFRA1/ITGA4/CXCR6/SELE/CCR5/CD8A/CXCL12/KCNJ3/GRIA2/SCARA5/GFRA3/IGHV3-64/CLEC10A/PTPRC/MS4A2/PKHD1/TLR8/FGA/XCR1/IGHV6-1/IGHV2-5/IGHV3-74/EPHA5/CLEC17A/IGHV4-4/FGB/GLRA1/IGHV3-20/IGHV3-7/IGHV3-15/CXCR5/*	73

(*Continued*)

 © 2023. The Author(s).

Supplementary Table S1 (*Continued*)

Ontology	ID	Description	GeneRatio	BgRatio	p-Value	*p*.adjust	q-Value	geneID	Count
								IGHV1-45/CXCL9/IGHV3-72/SERPINA5/IGHV3-48/IGHV2-26/IGHV3-49/IGHV4-28/MS4A1/IGHV5-51/IGHV1-69/IGHV4-39/IGHV4-59/IGHA2/IGHV3-21/FGG/IGHV3-13/IGHV1-18	
CC	GO:0098793	Presynapse	72/967	492/19,594	8.81E-17	1.38E-14	1.06E-14	*SLC1A2/ERBB4/CTTNBP2/CEL/NTRK2/P2RX1/SYT6/NLGN1/KCNA2/SV2B/RAB3C/KCTD16/DNAJC6/SH3GL3/SLC8A3/SLC8A2/CACNB2/SLC6A4/SLC8A1/BSN/NRXN1/SCAMP5/PACSIN1/KCNA3/PCLO/CHRNB2/SLC4A10/GRIK5/ATP2B2/SYT4/CRHBP/SLC18A3/UNC13A/CADPS/LRRK2/SLC29A4/RGS7BP/PCSK1/SYP/SYT9/SCGN/SLC18A2/SVOP/AVPR1B/KCNJ3/TRIM9/CNR1/RIMS2/SNAP25/LGI3/RIMS1/PRKCB/SNAP91/SH3GL2/CASR/ABCC8/GPM6A/CADM3/CPLX2/CDH10/KCTD8/GAD2/SYT5/ATCAY/NPY/SLC6A17/PTPRN/CNTN5/GABRA2/UCN3/GRM4/SYT8*	72
CC	GO:0043025	Neuronal cell body	69/967	482/19,594	1.25E-15	1.47E-13	1.12E-13	*PRKAR2B/SORBS2/CTNND2/TTLL7/SERPINI1/AMIGO1/CX3CR1/KCNA2/RTN4RL1/MAP2/CNKSR2/VIP/SLC8A3/SLC8A2/RIC3/KCNB1/SLC5A7/SLC8A1/NRXN1/RET/KCND3/NEURL1/OLFM1/SLC4A10/PRPH/DLG2/ADCYAP1/GRIK5/ATP2B2/ASTN1/SYT4/MAPT/CRHBP/CCR2/LRRK2/CNTN2/RGS7BP/HPN/CNGA3/PCSK1/KIF5C/GFRA1/MAPK8IP2/DSCAM/ELAVL4/GLRA3/ITGA4/TUBB4A/DPYSL5/CACNA1B/ATP1A3/AVPR1B/GRIA2/NRSN1/KCNB2/PCSK2/GRIA4/CASR/GPM6A/SEZ6/ASCL1/CPLX2/EPHA5/SYT5/NPY/GLRA1/PTPRN/GABRA2/SST*	69
CC	GO:0097060	Synaptic membrane	59/967	373/19,594	1.69E-15	1.59E-13	1.21E-13	*SLC1A2/ERBB4/NRCAM/CEL/CHRNB3/SYNE1/P2RX1/NLGN1/KCNA2/KCTD16/GABRB3/CNKSR2/ADCY1/CHRM3/DMD/KCNB1/MAGEE1/SLC6A4/SLC8A1/NRXN1/KCNA3/KCND3/LRRTM2/CHRNB2/DLG2/SLITRK1/GRIK5/ATP2B2/MUSK/ANK2/UNC13A/CNTN2/SHISA9/RGS7BP/SYP/GLRA3/CBLN1/CACNG5/KCNJ3/CHRM2/GRIA2/CNR1/RIMS2/SNAP25/RIMS1/SNAP91/GRIA4/GABRA4/GABRG2/GPM6A/CADM3/CDH10/KCTD8/GAD2/GLRA1/CNTN5/GABRA2/LHFPL4/LRRTM3*	59
CC	GO:0043204	Perikaryon	34/967	153/19,594	9.19E-14	7.21E-12	5.51E-12	*CTNND2/TTLL7/SERPINI1/AMIGO1/KCNA2/RTN4RL1/VIP/SLC8A3/SLC8A2/KCNB1/SLC5A7/NEURL1/OLFM1/SLC4A10/PRPH/DLG2/ADCYAP1/GRIK5/ASTN1/CRHBP/CCR2/LRRK2/RGS7BP/CNGA3/PCSK1/ELAVL4/GLRA3/AVPR1B/KCNB2/PCSK2/CPLX2/NPY/GLRA1/PTPRN*	34

 © 2023. The Author(s).

Supplementary Table S1 (*Continued*)

Ontology	ID	Description	GeneRatio	BgRatio	p-Value	*p*.adjust	q-Value	geneID	Count
CC	GO:0034702	Ion channel complex	48/967	294/19,594	2.28E-13	1.53E-11	1.17E-11	*CHRNB3/SCNN1B/KCNJ5/KCNA2/KCNAB1/AKAP6/KCNJ16/GABRB3/DPP10/SCN3A/CACNB2/KCNB1/KCNA3/SCN4A/UNC80/KCND3/CHRNB2/SCNN1G/GRIK5/ABCC9/KCNV1/SHISA9/KCNJ11/CACNA2D2/SCN3B/CNGA3/SCN7A/GLRA3/CACNA1B/CACNG5/DPP6/KCNA5/KCNJ3/KCNMB2/GRIA2/HCN4/KCNB2/KCNG3/GRIA4/GABRA4/GABRG2/ABCC8/TRPV6/CFTR/KCNJ6/CLIC3/GLRA1/GABRA2*	48
CC	GO:0042571	Immunoglobulin complex, circulating	23/967	77/19,594	1.30E-12	7.66E-11	5.86E-11	*IGHV3-64/IGHV6-1/IGHV2-5/IGHV3-74/IGHV4-4/IGHV3-20/IGHV3-7/IGHV3-15/IGHV1-45/JCHAIN/IGHV3-72/IGHV3-48/IGHV2-26/IGHV3-49/IGHV4-28/IGHV5-51/IGHV1-69/IGHV4-39/IGHV4-59/IGHA2/IGHV3-21/IGHV3-13/IGHV1-18*	23
CC	GO:0045211	Postsynaptic membrane	44/967	271/19,594	2.79E-12	1.46E-10	1.12E-10	*ERBB4/NRCAM/CEL/CHRNB3/SYNE1/P2RX1/NLGN1/KCTD16/GABRB3/CNKSR2/ADCY1/CHRM3/DMD/KCNB1/MAGEE1/SLC6A4/SLC8A1/KCNA3/KCND3/LRRTM2/CHRNB2/DLG2/SLITRK1/GRIK5/ATP2B2/MUSK/ANK2/CNTN2/SHISA9/RGS7BP/GLRA3/CBLN1/CACNG5/CHRM2/GRIA2/GRIA4/GABRA4/GABRG2/CDH10/KCTD8/GLRA1/GABRA2/LHFPL4/LRRTM3*	44
CC	GO:0034703	Cation channel complex	38/967	221/19,594	1.49E-11	7.00E-10	5.35E-10	*SCNN1B/KCNJ5/KCNA2/KCNAB1/AKAP6/KCNJ16/DPP10/SCN3A/CACNB2/KCNB1/KCNA3/SCN4A/UNC80/KCND3/SCNN1G/GRIK5/ABCC9/KCNV1/SHISA9/KCNJ11/CACNA2D2/SCN3B/CNGA3/SCN7A/CACNA1B/CACNG5/DPP6/KCNA5/KCNJ3/KCNMB2/GRIA2/HCN4/KCNB2/KCNG3/GRIA4/ABCC8/TRPV6/KCNJ6*	38
CC	GO:0099240	Intrinsic component of synaptic membrane	31/967	160/19,594	4.96E-11	2.12E-09	1.62E-09	*SLC1A2/ERBB4/NRCAM/CEL/P2RX1/NLGN1/KCNA2/ADCY1/SLC6A4/SLC8A1/KCNA3/KCND3/LRRTM2/CHRNB2/SLITRK1/GRIK5/ATP2B2/CNTN2/SHISA9/RGS7BP/CACNG5/KCNJ3/CNR1/GABRA4/GABRG2/GPM6A/CADM3/CDH10/CNTN5/GABRA2/LRRTM3*	31
CC	GO:0044306	Neuron projection terminus	27/967	129/19,594	1.40E-10	5.49E-09	4.20E-09	*SLC1A2/NTRK2/KCNA2/SLC8A3/SLC8A2/DMD/SLC8A1/BSN/PACSIN1/KCNA3/SLC4A10/GRIK5/CRHBP/SLC18A3/UNC13A/LRRK2/PCSK1/SYP/SCGN/SLC18A2/AVPR1B/PRKCB/CASR/CPLX2/NPY/PTPRN/UCN3*	27
CC	GO:1902495	Transmembrane transporter complex	50/967	377/19,594	1.87E-10	6.77E-09	5.18E-09	*CHRNB3/SCNN1B/KCNJ5/KCNA2/KCNAB1/AKAP6/KCNJ16/GABRB3/DPP10/FXYD2/SCN3A/CACNB2/KCNB1/KCNA3/SCN4A/UNC80/KCND3/CHRNB2/SCNN1G/GRIK5/ABCC9/KCNV1/SHISA9/KCNJ11/CACNA2D2/SCN3B/CNGA3/SCN7A/GLRA3/CACNA1B/CACNG5/ATP1A3/DPP6/KCNA5/KCNJ3/KCNMB2/*	50

(*Continued*)

 © 2023. The Author(s).

Supplementary Table S1 (*Continued*)

Ontology	ID	Description	GeneRatio	BgRatio	p-Value	*p*.adjust	q-Value	geneID	Count
								GRIA2/HCN4/KCNB2/KCNG3/GRIA4/GABRA4/GABRG2/ABCC8/TRPV6/CFTR/KCNJ6/CLIC3/GLRA1/GABRA2	
CC	GO:0150034	Distal axon	40/967	270/19,594	4.77E-10	1.60E-08	1.23E-08	*BRSK2/NTRK2/KCNA2/MAP2/SLC8A3/SLC8A2/SLC8A1/PACSIN1/KCNA3/OLFM1/SLC4A10/GRIK5/PCDH9/MAPT/CRHBP/SLC18A3/UNC13A/LRRK2/PCSK1/KIF5C/SYP/SCGN/DSCAM/ELAVL4/SLC18A2/ITGA4/STMN4/AVPR1B/CNR1/SNAP25/NRSN1/PRKCB/CASR/GPM6A/CPLX2/ATCAY/NPY/PTPRN/CDK5R2/UCN3*	40
CC	GO:0099699	Integral component of synaptic membrane	28/967	149/19,594	8.86E-10	2.78E-08	2.13E-08	*SLC1A2/ERBB4/NRCAM/CEL/P2RX1/NLGN1/KCNA2/ADCY1/SLC6A4/SLC8A1/KCNA3/KCND3/LRRTM2/CHRNB2/SLITRK1/GRIK5/ATP2B2/SHISA9/CACNG5/KCNJ3/CNR1/GABRA4/GABRG2/GPM6A/CADM3/CDH10/GABRA2/LRRTM3*	28
CC	GO:0031045	Dense core granule	12/967	26/19,594	9.92E-10	2.92E-08	2.23E-08	*SPX/MYRIP/KIF1A/SYT4/CRHBP/CADPS/SYT9/SCG2/SYT5/NPY/CHGA/SYT8*	12
CC	GO:0043679	Axon terminus	24/967	113/19,594	1.07E-09	2.98E-08	2.27E-08	*NTRK2/KCNA2/SLC8A3/SLC8A2/SLC8A1/PACSIN1/KCNA3/SLC4A10/GRIK5/CRHBP/SLC18A3/UNC13A/LRRK2/PCSK1/SYP/SCGN/SLC18A2/AVPR1B/PRKCB/CASR/CPLX2/NPY/PTPRN/UCN3*	24
CC	GO:1990351	Transporter complex	50/967	399/19,594	1.36E-09	3.57E-08	2.73E-08	*CHRNB3/SCNN1B/KCNJ5/KCNA2/KCNAB1/AKAP6/KCNJ16/GABRB3/DPP10/FXYD2/SCN3A/CACNB2/KCNB1/KCNA3/SCN4A/UNC80/KCND3/CHRNB2/SCNN1G/GRIK5/ABCC9/KCNV1/SHISA9/KCNJ11/CACNA2D2/SCN3B/CNGA3/SCN7A/GLRA3/CACNA1B/CACNG5/ATP1A3/DPP6/KCNA5/KCNJ3/KCNMB2/GRIA2/HCN4/KCNB2/KCNG3/GRIA4/GABRA4/GABRG2/ABCC8/TRPV6/CFTR/KCNJ6/CLIC3/GLRA1/GABRA2*	50
CC	GO:0034705	Potassium channel complex	21/967	89/19,594	1.56E-09	3.86E-08	2.95E-08	*KCNJ5/KCNA2/KCNAB1/KCNJ16/DPP10/KCNB1/KCNA3/KCND3/GRIK5/ABCC9/KCNV1/KCNJ11/DPP6/KCNA5/KCNJ3/KCNMB2/HCN4/KCNB2/KCNG3/ABCC8/KCNJ6*	21
CC	GO:0098936	Intrinsic component of postsynaptic membrane	24/967	123/19,594	6.45E-09	1.52E-07	1.16E-07	*ERBB4/NRCAM/CEL/P2RX1/NLGN1/ADCY1/SLC6A4/SLC8A1/KCNA3/KCND3/LRRTM2/CHRNB2/SLITRK1/GRIK5/ATP2B2/CNTN2/SHISA9/RGS7BP/CACNG5/GABRA4/GABRG2/CDH10/GABRA2/LRRTM3*	24

 © 2023. The Author(s).

Supplementary Table S1 (*Continued*)

Ontology	ID	Description	GeneRatio	BgRatio	p-Value	*p*.adjust	q-Value	geneID	Count
CC	GO:0042734	Presynaptic membrane	26/967	143/19,594	7.28E-09	1.63E-07	1.25E-07	*SLC1A2/ERBB4/P2RX1/KCNA2/KCTD16/SLC6A4/NRXN1/KCNA3/CHRNB2/GRIK5/ATP2B2/UNC13A/RGS7BP/SYP/KCNJ3/CNR1/RIMS2/SNAP25/RIMS1/SNAP91/GPM6A/CADM3/CDH10/KCTD8/GAD2/CNTN5*	26
CC	GO:0062023	Collagen-containing extracellular matrix	49/967	429/19,594	4.12E-08	8.83E-07	6.75E-07	*FGL1/CLU/PRSS1/CMA1/LAMB4/MMRN1/NCAM1/GDF10/SBSPON/ANGPTL1/ATRNL1/AMBP/TNR/VTN/FCN2/COL21A1/CTSG/APOH/SFRP1/MATN4/FREM1/COL25A1/TNXB/APLP1/CBLN1/ABI3BP/CXCL12/COL14A1/MYOC/OGN/COL7A1/THBS4/FGA/CBLN4/COL19A1/FGF10/VIT/ADIPOQ/FGB/KRT1/COL11A1/FIBCD1/SERPINA5/ANGPTL7/COL17A1/APCS/LRRC15/ZG16/FGG*	49
CC	GO:0099634	Postsynaptic specialization membrane	22/967	116/19,594	4.69E-08	9.61E-07	7.35E-07	*ERBB4/NRCAM/CEL/NLGN1/CNKSR2/ADCY1/KCND3/LRRTM2/CHRNB2/DLG2/SLITRK1/GRIK5/ATP2B2/SHISA9/RGS7BP/CACNG5/GRIA2/GABRA4/GABRG2/CDH10/GABRA2/LRRTM3*	22
CC	GO:0098948	Intrinsic component of postsynaptic specialization membrane	18/967	80/19,594	5.04E-08	9.89E-07	7.56E-07	*ERBB4/NRCAM/CEL/NLGN1/ADCY1/KCND3/LRRTM2/CHRNB2/SLITRK1/GRIK5/ATP2B2/SHISA9/RGS7BP/GABRA4/GABRG2/CDH10/GABRA2/LRRTM3*	18
CC	GO:0099055	Integral component of postsynaptic membrane	22/967	118/19,594	6.47E-08	1.22E-06	9.31E-07	*ERBB4/NRCAM/CEL/P2RX1/NLGN1/ADCY1/SLC6A4/SLC8A1/KCNA3/KCND3/LRRTM2/CHRNB2/SLITRK1/GRIK5/ATP2B2/SHISA9/CACNG5/GABRA4/GABRG2/CDH10/GABRA2/LRRTM3*	22
CC	GO:0099060	Integral component of postsynaptic specialization membrane	17/967	77/19,594	1.59E-07	2.87E-06	2.20E-06	*ERBB4/NRCAM/CEL/NLGN1/ADCY1/KCND3/LRRTM2/CHRNB2/SLITRK1/GRIK5/ATP2B2/SHISA9/GABRA4/GABRG2/CDH10/GABRA2/LRRTM3*	17
CC	GO:0098978	Glutamatergic synapse	39/967	319/19,594	1.72E-07	2.99E-06	2.29E-06	*SLC1A2/PRKAR2B/ERBB4/NRCAM/CTTNBP2/P2RX1/NLGN1/PAK3/CNKSR2/SH3GL3/ADCY1/HOMER2/BSN/TNR/KCNA3/PCLO/LRRTM2/GHRL/SLITRK1/GRIK5/ATP2B2/SYT4/CADPS/LRRK2/SHISA9/RGS7BP/ELAVL4/CBLN1/CACNG5/CNR1/SNAP25/SH3GL2/GABRG2/GPM6A/CPLX2/CDH10/SLC6A17/CALY/LRRTM3*	39
CC	GO:0008076	Voltage-gated potassium channel complex	17/967	79/19,594	2.35E-07	3.96E-06	3.03E-06	*KCNJ5/KCNA2/KCNAB1/KCNJ16/DPP10/KCNB1/KCNA3/KCND3/KCNV1/DPP6/KCNA5/KCNJ3/KCNMB2/HCN4/KCNB2/KCNG3/KCNJ6*	17

(*Continued*)

 © 2023. The Author(s).

Supplementary Table S1 (*Continued*)

Ontology	ID	Description	GeneRatio	BgRatio	p-Value	*p*.adjust	q-Value	geneID	Count
CC	GO:0098889	Intrinsic component of presynaptic membrane	16/967	74/19,594	4.95E-07	8.05E-06	6.15E-06	*SLC1A2/ERBB4/P2RX1/KCNA2/SLC6A4/KCNA3/CHRNB2/GRIK5/ATP2B2/RGS7BP/KCNJ3/CNR1/GPM6A/CADM3/CDH10/CNTN5*	16
CC	GO:0072562	Blood microparticle	23/967	147/19,594	8.78E-07	1.38E-05	1.05E-05	*CLU/ALB/PRSS1/GC/AMBP/VTN/FCN2/AFM/C8A/IGKV2D-28/C4BPA/FGA/FGB/KRT1/IGHV3-7/IGKV1-39/JCHAIN/IGKV1-17/APCS/IGKV4-1/IGHA2/FGG/IGHV3-13*	23
CC	GO:0098992	Neuronal dense core vesicle	7/967	13/19,594	9.22E-07	1.40E-05	1.07E-05	*KIF1A/SYT4/CADPS/SCG2/SYT5/NPY/CHGA*	7
CC	GO:0099572	Postsynaptic specialization	39/967	341/19,594	9.59E-07	1.41E-05	1.08E-05	*ERBB4/NRCAM/CEL/NTRK2/CTNND2/NLGN1/NOS1/BNIP3/PAK3/CNKSR2/DNAJC6/SH3GL3/ADCY1/SLC8A3/SLC8A2/ANKS1B/HOMER2/SLC8A1/BSN/PCLO/KCND3/NEURL1/LRRTM2/CHRNB2/DLG2/SLITRK1/GRIK5/BCL11A/ATP2B2/SHISA9/RGS7BP/MAPK8IP2/CACNG5/GRIA2/GABRA4/GABRG2/CDH10/GABRA2/LRRTM3*	39
CC	GO:0044304	Main axon	14/967	61/19,594	1.19E-06	1.70E-05	1.30E-05	*SLC1A2/NRCAM/KCNA2/KCNAB1/MAP2/NFASC/DLG2/MAPT/CRHBP/CNTN2/CNGA3/TUBB4A/MYOC/UCN3*	14
CC	GO:0098982	GABA-ergic synapse	15/967	70/19,594	1.27E-06	1.76E-05	1.34E-05	*ERBB4/BSN/PCLO/KCND3/LRRTM2/SLITRK1/ATP2B2/CNR1/GABRA4/GABRG2/CDH10/CBLN4/SLC6A17/CNTN5/GABRA2*	15
CC	GO:0030672	Synaptic vesicle membrane	18/967	103/19,594	2.66E-06	3.49E-05	2.66E-05	*SYT6/SV2B/RAB3C/BSN/SCAMP5/SYT4/UNC13A/LRRK2/SYP/SYT9/SLC18A2/SVOP/ABCC8/GAD2/SYT5/SLC6A17/GABRA2/SYT8*	18
CC	GO:0099501	Exocytic vesicle membrane	18/967	103/19,594	2.66E-06	3.49E-05	2.66E-05	*SYT6/SV2B/RAB3C/BSN/SCAMP5/SYT4/UNC13A/LRRK2/SYP/SYT9/SLC18A2/SVOP/ABCC8/GAD2/SYT5/SLC6A17/GABRA2/SYT8*	18
CC	GO:0099056	Integral component of presynaptic membrane	14/967	67/19,594	3.92E-06	4.99E-05	3.81E-05	*SLC1A2/ERBB4/P2RX1/KCNA2/SLC6A4/KCNA3/CHRNB2/GRIK5/ATP2B2/KCNJ3/CNR1/GPM6A/CADM3/CDH10*	14
CC	GO:0016324	Apical plasma membrane	37/967	358/19,594	1.86E-05	0.00023	0.000176	*SCNN1B/SORBS2/ABCB1/UMOD/GP2/CA4/TRPM6/KL/CD36/SLC38A3/SLC2A2/SLC26A4/SLC4A10/SCNN1G/PDZK1/ATP2B2/SLC17A4/AJAP1/CD300LG/DPEP1/ANK2/ADGRG2/SLC17A1/SLC29A4/HPN/SLC3A1/KCNA5/SLC5A8/SLC10A2/CASR/ATP4A/PKHD1/CBLIF/CYP4A11/CFTR/PAPPA2/SLC26A9*	37

 © 2023. The Author(s).

Supplementary Table S1 (*Continued*)

Ontology	ID	Description	GeneRatio	BgRatio	p-Value	*p*.adjust	q-Value	geneID	Count
CC	GO:0098984	Neuron to neuron synapse	36/967	347/19,594	2.21E-05	0.000267	0.000204	*ERBB4/NRCAM/NTRK2/CTNND2/NLGN1/NOS1/BNIP3/PAK3/CNKSR2/DNAJC6/SH3GL3/AKAP7/ADCY1/SLC8A3/SLC8A2/ANKS1B/HOMER2/SLC8A1/BSN/PCLO/KCND3/NEURL1/LRRTM2/DLG2/SLITRK1/GRIK5/BCL11A/ATP2B2/SHISA9/RGS7BP/SYT9/MAPK8IP2/CACNG5/ATP1A3/GRIA2/LRRTM3*	36
CC	GO:0030658	Transport vesicle membrane	25/967	205/19,594	2.84E-05	0.000335	0.000256	*SYT6/CA4/SV2B/RAB3C/BSN/SCAMP5/SYT4/SLC18A3/UNC13A/LRRK2/SYP/SYT9/SCGN/SLC18A2/SCG3/SVOP/CPE/ABCC8/GAD2/SYT5/SLC6A17/PTPRN/SLC30A8/GABRA2/SYT8*	25
CC	GO:0014069	Postsynaptic density	33/967	318/19,594	4.77E-05	0.000548	0.000419	*ERBB4/NRCAM/NTRK2/CTNND2/NLGN1/NOS1/BNIP3/PAK3/CNKSR2/DNAJC6/SH3GL3/ADCY1/SLC8A3/SLC8A2/ANKS1B/HOMER2/SLC8A1/BSN/PCLO/KCND3/NEURL1/LRRTM2/DLG2/SLITRK1/GRIK5/BCL11A/ATP2B2/SHISA9/RGS7BP/MAPK8IP2/CACNG5/GRIA2/LRRTM3*	33
CC	GO:0032279	Asymmetric synapse	33/967	323/19,594	6.48E-05	0.000727	0.000556	*ERBB4/NRCAM/NTRK2/CTNND2/NLGN1/NOS1/BNIP3/PAK3/CNKSR2/DNAJC6/SH3GL3/ADCY1/SLC8A3/SLC8A2/ANKS1B/HOMER2/SLC8A1/BSN/PCLO/KCND3/NEURL1/LRRTM2/DLG2/SLITRK1/GRIK5/BCL11A/ATP2B2/SHISA9/RGS7BP/MAPK8IP2/CACNG5/GRIA2/LRRTM3*	33
CC	GO:0045177	Apical part of cell	40/967	424/19,594	6.95E-05	0.000762	0.000582	*AQP8/EPB41L4B/SCNN1B/SORBS2/ABCB1/UMOD/GP2/CA4/TRPM6/KL/CD36/HOMER2/SLC38A3/SLC2A2/SLC26A4/SLC4A10/SCNN1G/PDZK1/ATP2B2/SLC17A4/AJAP1/CD300LG/DPEP1/ANK2/ADGRG2/SLC17A1/SLC29A4/HPN/SLC3A1/KCNA5/SLC5A8/SLC10A2/CASR/ATP4A/PKHD1/CBLIF/CYP4A11/CFTR/PAPPA2/SLC26A9*	40
CC	GO:0043195	Terminal bouton	10/967	48/19,594	9.66E-05	0.001022	0.000781	*NTRK2/GRIK5/SLC18A3/UNC13A/LRRK2/SYP/SCGN/SLC18A2/CPLX2/NPY*	10
CC	GO:0044298	Cell body membrane	8/967	31/19,594	9.77E-05	0.001022	0.000781	*AMIGO1/CX3CR1/KCNA2/P2RY12/KCNB1/ATP2B2/ATP1A3/KCNB2*	8
CC	GO:0030133	Transport vesicle	38/967	402/19,594	0.0001	0.001028	0.000786	*SPX/MYRIP/CTTNBP2/SYT6/CA4/SV2B/RAB3C/BSN/SCAMP5/SYT4/SLC18A3/UNC13A/LRRK2/IL33/PCSK1/SYP/SYT9/SCGN/SLC18A2/SCG3/SVOP/CPE/TRIM9/SNAP25/NRSN1/LGI3/SNAP91/PCSK2/ABCC8/GAD2/SYT5/IGF1/CHGA/SLC6A17/PTPRN/SLC30A8/GABRA2/SYT8*	38

(*Continued*)

 © 2023. The Author(s).

Global Medical Genetics © 2023. The Author(s).

Supplementary Table S1 (*Continued*)

Ontology	ID	Description	GeneRatio	BgRatio	p-Value	*p*.adjust	q-Value	geneID	Count
CC	GO:0008021	Synaptic vesicle	23/967	196/19,594	0.000106	0.001063	0.000812	*CTTNBP2/SYT6/SV2B/RAB3C/BSN/SCAMP5/SYT4/UNC13A/LRRK2/SYP/SYT9/SLC18A2/SVOP/TRIM9/SNAP25/LGI3/SNAP91/ABCC8/GAD2/SYT5/SLC6A17/GABRA2/SYT8*	23
CC	GO:0098839	Postsynaptic density membrane	14/967	89/19,594	0.000111	0.001093	0.000835	*ERBB4/NRCAM/CNKSR2/ADCY1/LRRTM2/DLG2/SLITRK1/GRIK5/ATP2B2/SHISA9/RGS7BP/CACNG5/GRIA2/LRRTM3*	14
CC	GO:0016323	Basolateral plasma membrane	25/967	226/19,594	0.000141	0.001355	0.001035	*ERBB4/UMOD/SLC16A12/SLCO4C1/KCNJ16/CHRM3/SLC8A2/SLC38A3/AQP4/SLCO1C1/TSHR/SLC4A4/SLC4A10/DLG2/ATP2B2/AJAP1/CD300LG/ANK2/SLC29A4/ABCA8/SLC22A9/CASR/SLC39A5/MTTP/CA9*	25
CC	GO:0070382	Exocytic vesicle	24/967	214/19,594	0.000154	0.00145	0.001108	*CTTNBP2/SYT6/SV2B/RAB3C/BSN/SCAMP5/SYT4/UNC13A/LRRK2/SYP/SYT9/SLC18A2/SVOP/TRIM9/SNAP25/LGI3/SNAP91/ABCC8/GAD2/SYT5/IGF1/SLC6A17/GABRA2/SYT8*	24
CC	GO:0034706	Sodium channel complex	7/967	26/19,594	0.000201	0.001855	0.001418	*SCNN1B/SCN3A/SCN4A/SCNN1G/GRIK5/SCN3B/SCN7A*	7
CC	GO:0099146	Intrinsic component of postsynaptic density membrane	10/967	54/19,594	0.00027	0.002447	0.00187	*ERBB4/NRCAM/ADCY1/LRRTM2/SLITRK1/GRIK5/ATP2B2/SHISA9/RGS7BP/LRRTM3*	10
CC	GO:0043194	Axon initial segment	6/967	20/19,594	0.000303	0.002697	0.002061	*NRCAM/KCNA2/MAP2/NFASC/CNTN2/CNGA3*	6
CC	GO:0043197	Dendritic spine	20/967	172/19,594	0.000329	0.00284	0.00217	*PRKAR2B/CTTNBP2/NTRK2/NLGN1/NOS1/SLC8A3/SLC8A2/ANKS1B/SLC8A1/KCND3/NEURL1/ATP2B2/MAPT/SHISA9/RGS7BP/GRIA2/GRIA4/ITPKA/GPM6A/SEZ6*	20
CC	GO:0032809	Neuronal cell body membrane	7/967	28/19,594	0.000332	0.00284	0.00217	*AMIGO1/CX3CR1/KCNA2/KCNB1/ATP2B2/ATP1A3/KCNB2*	7
CC	GO:0044309	Neuron spine	20/967	173/19,594	0.000355	0.00299	0.002285	*PRKAR2B/CTTNBP2/NTRK2/NLGN1/NOS1/SLC8A3/SLC8A2/ANKS1B/SLC8A1/KCND3/NEURL1/ATP2B2/MAPT/SHISA9/RGS7BP/GRIA2/GRIA4/ITPKA/GPM6A/SEZ6*	20
CC	GO:0097440	Apical dendrite	5/967	15/19,594	0.000575	0.004748	0.003629	*CLU/MAP2/NEURL1/SLC4A10/SEZ6*	5
CC	GO:0009925	Basal plasma membrane	25/967	251/19,594	0.000696	0.005648	0.004317	*ERBB4/UMOD/SLC16A12/SLCO4C1/KCNJ16/CHRM3/SLC8A2/SLC38A3/AQP4/SLCO1C1/TSHR/SLC4A4/SLC4A10/DLG2/ATP2B2/AJAP1/CD300LG/ANK2/SLC29A4/ABCA8/SLC22A9/CASR/SLC39A5/MTTP/CA9*	25
CC	GO:0042383	Sarcolemma	16/967	131/19,594	0.000736	0.005872	0.004488	*SNTG2/NOS1/AKAP6/SLC8A3/SLC8A2/DMD/CACNB2/KCNB1/SLC8A1/AQP4/KCND3/ANK2/KCNJ11/PGM5/KCNJ3/ABCC8*	16

Supplementary Table S1 (*Continued*)

Ontology	ID	Description	GeneRatio	BgRatio	p-Value	*p*.adjust	q-Value	geneID	Count
CC	GO:0099061	Integral component of postsynaptic density membrane	9/967	51/19,594	0.000784	0.006155	0.004704	*ERBB4/NRCAM/ADCY1/LRRTM2/SLITRK1/GRIK5/ATP2B2/SHISA9/LRRTM3*	9
CC	GO:0098831	Presynaptic active zone cytoplasmic component	5/967	16/19,594	0.000802	0.006192	0.004733	*BSN/PCLO/UNC13A/RIMS2/RIMS1*	5
CC	GO:0097386	Glial cell projection	7/967	33/19,594	0.000965	0.007172	0.005481	*SLC1A2/NFASC/AQP4/SYT4/MAPT/CNGA3/SCN7A*	7
CC	GO:0044224	Juxtaparanode region of axon	4/967	10/19,594	0.000974	0.007172	0.005481	*KCNA2/KCNAB1/DLG2/CNTN2*	4
CC	GO:0099569	Presynaptic cytoskeleton	4/967	10/19,594	0.000974	0.007172	0.005481	*BSN/PCLO/RIMS2/RIMS1*	4
CC	GO:0031091	Platelet alpha granule	12/967	91/19,594	0.00169	0.012245	0.009359	*CLU/ALB/CD36/MMRN1/SELP/EGF/VEGFD/FGA/IGF1/FGB/SERPINA5/FGG*	12
CC	GO:0031225	Anchored component of membrane	18/967	169/19,594	0.00177	0.012634	0.009656	*UMOD/GP2/CA4/RTN4RL1/RAB3C/NCAM1/DPEP1/CNTN2/RGS7BP/GFRA1/ART4/ART3/GFRA3/PKHD1/GAD2/CNTN5/XPNPEP2/MSLN*	18
CC	GO:0045178	Basal part of cell	25/967	269/19,594	0.001849	0.013	0.009937	*ERBB4/UMOD/SLC16A12/SLCO4C1/KCNJ16/CHRM3/SLC8A2/SLC38A3/AQP4/SLCO1C1/TSHR/SLC4A4/SLC4A10/DLG2/ATP2B2/AJAP1/CD300LG/ANK2/SLC29A4/ABCA8/SLC22A9/CASR/SLC39A5/MTTP/CA9*	25
CC	GO:0060077	Inhibitory synapse	5/967	19/19,594	0.001887	0.013069	0.009989	*GABRG2/GAD2/GLRA1/GABRA2/LHFPL4*	5
CC	GO:0032589	Neuron projection membrane	9/967	58/19,594	0.002017	0.013768	0.010523	*SLC1A2/ADGRV1/KCNB1/ATP2B2/MAPT/SHISA9/GABRA4/GABRG2/GABRA2*	9
CC	GO:0045121	Membrane raft	28/967	326/19,594	0.003237	0.021783	0.016649	*SLC1A2/PRKAR2B/P2RX1/GP2/NOS1/AKAP6/RTN4RL1/CD36/FYB2/ADCY1/DMD/SLC6A4/FASLG/TNR/KCNA3/RET/CHRNB2/PDZK1/ATP2B2/MAPT/ANK2/LRRK2/SELE/KCNA5/CD8A/CNR1/PTPRC/MS4A1*	28
CC	GO:0060076	Excitatory synapse	8/967	51/19,594	0.003295	0.021859	0.016708	*NLGN1/BSN/LRRTM2/ATP2B2/SYP/CBLN1/KCNJ3/GRIA2*	8
CC	GO:0098857	Membrane microdomain	28/967	327/19,594	0.003379	0.022106	0.016896	*SLC1A2/PRKAR2B/P2RX1/GP2/NOS1/AKAP6/RTN4RL1/CD36/FYB2/ADCY1/DMD/SLC6A4/FASLG/TNR/KCNA3/RET/CHRNB2/PDZK1/ATP2B2/MAPT/ANK2/LRRK2/SELE/KCNA5/CD8A/CNR1/PTPRC/MS4A1*	28
CC	GO:0048786	Presynaptic active zone	10/967	75/19,594	0.003685	0.023736	0.018142	*P2RX1/BSN/PCLO/ATP2B2/UNC13A/SYP/RIMS2/RIMS1/GPM6A/CDH10*	10
CC	GO:0034707	Chloride channel complex	8/967	52/19,594	0.003729	0.023736	0.018142	*GABRB3/GLRA3/GABRA4/GABRG2/CFTR/CLIC3/GLRA1/GABRA2*	8

(*Continued*)

 © 2023. The Author(s).

Supplementary Table S1 (*Continued*)

Ontology	ID	Description	GeneRatio	BgRatio	p-Value	*p*.adjust	q-Value	geneID	Count
CC	GO:0099059	Integral component of presynaptic active zone membrane	4/967	14/19,594	0.003965	0.024898	0.01903	*P2RX1/ATP2B2/GPM6A/CDH10*	4
CC	GO:0031093	Platelet alpha granule lumen	9/967	67/19,594	0.005462	0.033851	0.025874	*CLU/ALB/MMRN1/EGF/VEGFD/FGA/IGF1/FGB/FGG*	9
CC	GO:0030426	Growth cone	16/967	161/19,594	0.006097	0.037292	0.028504	*MAP2/OLFM1/PCDH9/MAPT/LRRK2/KIF5C/DSCAM/ELAVL4/ITGA4/STMN4/CNR1/SNAP25/NRSN1/GPM6A/ATCAY/CDK5R2*	16
CC	GO:0031528	Microvillus membrane	5/967	26/19,594	0.008018	0.048419	0.037008	*PDZK1/DPEP1/MTTP/S100P/CA9*	5
CC	GO:0001518	Voltage-gated sodium channel complex	4/967	17/19,594	0.008377	0.049321	0.037697	*SCN3A/SCN4A/SCN3B/SCN7A*	4
CC	GO:0098945	Intrinsic component of presynaptic active zone membrane	4/967	17/19,594	0.008377	0.049321	0.037697	*P2RX1/ATP2B2/GPM6A/CDH10*	4
CC	GO:0030427	Site of polarized growth	16/967	167/19,594	0.008581	0.0499	0.03814	*MAP2/OLFM1/PCDH9/MAPT/LRRK2/KIF5C/DSCAM/ELAVL4/ITGA4/STMN4/CNR1/SNAP25/NRSN1/GPM6A/ATCAY/CDK5R2*	16
MF	GO:0003823	Antigen binding	39/906	174/18,410	8.93E-16	7.23E-13	6.02E-13	*GP2/FCN2/ITGA4/TRAV29DV5/IGKV2D-28/IGHV3-64/IGHV6-1/IGHV2-5/IGHV3-74/IGHV4-4/IGHV3-20/IGLV7-43/IGKV1D-39/IGHV3-7/IGHV3-15/IGKV1-39/IGHV1-45/JCHAIN/IGKV5-2/IGLV2-8/IGHV3-72/IGKV1-17/IGHV3-48/IGHV2-26/IGHV3-49/IGHV4-28/IGLV6-57/IGKV1-16/IGHV5-51/IGLV3-27/IGKV4-1/IGHV1-69/IGLV3-19/IGHV4-39/IGHV4-59/IGHA2/IGHV3-21/IGHV3-13/IGHV1-18*	39
MF	GO:0022836	Gated channel activity	55/906	340/18,410	4.95E-15	1.34E-12	1.11E-12	*CHRNB3/KCNK3/SCNN1B/P2RX1/PEX5L/KCNJ5/KCNA2/KCNAB1/KCNJ16/GABRB3/KCNH7/SCN3A/CACNB2/ANO5/KCNB1/ANO3/KCNA3/SCN4A/KCND3/CHRNB2/SCNN1G/GRIK5/ABCC9/KCNV1/MCOLN3/KCNJ11/CACNA2D2/SCN3B/CNGA3/SCN7A/TRPM3/GLRA3/TMEM63C/CACNA1B/CACNG5/KCNA5/KCNJ3/KCNMB2/GRIA2/HCN4/CNR1/SNAP25/KCNB2/KCNG3/GRIA4/GABRA4/GABRG2/ABCC8/KCNH6/CFTR/KCNJ6/CLIC3/GLRA1/KCNK16/GABRA2*	55
MF	GO:0046873	Metal ion transmembrane transporter activity	63/906	428/18,410	4.95E-15	1.34E-12	1.11E-12	*SLC1A2/KCNK3/SCNN1B/KCNJ5/KCNA2/KCNAB1/TRPM6/KCNJ16/SLC5A9/KCNH7/SLC8A3/SLC8A2/SCN3A/SLC30A2/CACNB2/KCNB1/SLC5A7/SLC6A4/SLC8A1/SLC5A4/TUSC3/KCNA3/SCN4A/KCND3/SLC4A4/SLC4A10/SCNN1G/GRIK5/ATP2B2/SLC17A4/ABCC9/KCNV1/SLC6A15/MCOLN3/SLC17A1/KCNJ11/*	63

 © 2023. The Author(s).

Supplementary Table S1 (*Continued*)

Ontology	ID	Description	GeneRatio	BgRatio	p-Value	*p*.adjust	q-Value	geneID	Count
								CACNA2D2/SCN3B/SCN7A/TRPM3/SLC18A2/CACNA1B/CACNG5/ATP1A3/KCNA5/SLC5A8/KCNJ3/KCNMB2/HCN4/CNR1/SNAP25/SLC10A2/KCNB2/KCNG3/ATP4A/ABCC8/GPM6A/TRPV6/KCNH6/SLC39A5/KCNJ6/KCNK16/SLC30A8	
MF	GO:0022803	Passive transmembrane transporter activity	68/906	490/18,410	7.86E-15	1.59E-12	1.32E-12	*AQP8/CHRNB3/KCNK3/SCNN1B/P2RX1/PEX5L/KCNJ5/OTOP3/KCNA2/KCNAB1/AQP12B/TRPM6/KCNJ16/GABRB3/AQP12A/KCNH7/SCN3A/CACNB2/ANO5/KCNB1/ANO3/AQP4/KCNA3/SCN4A/UNC80/KCND3/CHRNB2/SCNN1G/GRIK5/ABCC9/KCNV1/MCOLN3/KCNJ11/CACNA2D2/SCN3B/CNGA3/SCN7A/TRPM3/GLRA3/TMEM63C/CACNA1B/CACNG5/KCNA5/SLC5A8/KCNJ3/KCNMB2/GRIA2/HCN4/CNR1/SNAP25/KCNB2/KCNG3/GRIA4/GABRA4/GABRG2/ABCC8/GPM6A/TRPV6/KCNH6/CFTR/KCNJ6/GJD2/CLIC3/GLRA1/KCNK16/GABRA2/SLC26A9/GJB4*	68
MF	GO:0015267	Channel activity	67/906	489/18,410	2.35E-14	3.80E-12	3.16E-12	*AQP8/CHRNB3/KCNK3/SCNN1B/P2RX1/PEX5L/KCNJ5/OTOP3/KCNA2/KCNAB1/AQP12B/TRPM6/KCNJ16/GABRB3/AQP12A/KCNH7/SCN3A/CACNB2/ANO5/KCNB1/ANO3/AQP4/KCNA3/SCN4A/UNC80/KCND3/CHRNB2/SCNN1G/GRIK5/ABCC9/KCNV1/MCOLN3/KCNJ11/CACNA2D2/SCN3B/CNGA3/SCN7A/TRPM3/GLRA3/TMEM63C/CACNA1B/CACNG5/KCNA5/KCNJ3/KCNMB2/GRIA2/HCN4/CNR1/SNAP25/KCNB2/KCNG3/GRIA4/GABRA4/GABRG2/ABCC8/GPM6A/TRPV6/KCNH6/CFTR/KCNJ6/GJD2/CLIC3/GLRA1/KCNK16/GABRA2/SLC26A9/GJB4*	67
MF	GO:0005216	Ion channel activity	61/906	442/18,410	2.58E-13	3.48E-11	2.90E-11	*CHRNB3/KCNK3/SCNN1B/P2RX1/PEX5L/KCNJ5/OTOP3/KCNA2/KCNAB1/TRPM6/KCNJ16/GABRB3/KCNH7/SCN3A/CACNB2/ANO5/KCNB1/ANO3/KCNA3/SCN4A/UNC80/KCND3/CHRNB2/SCNN1G/GRIK5/ABCC9/KCNV1/MCOLN3/KCNJ11/CACNA2D2/SCN3B/CNGA3/SCN7A/TRPM3/GLRA3/TMEM63C/CACNA1B/CACNG5/KCNA5/KCNJ3/KCNMB2/GRIA2/HCN4/CNR1/SNAP25/KCNB2/KCNG3/GRIA4/GABRA4/GABRG2/ABCC8/GPM6A/TRPV6/KCNH6/CFTR/KCNJ6/CLIC3/GLRA1/KCNK16/GABRA2/SLC26A9*	61
MF	GO:0034987	Immunoglobulin receptor binding	23/906	80/18,410	2.96E-12	3.42E-10	2.85E-10	*IGHV3-64/IGHV6-1/IGHV2-5/IGHV3-74/IGHV4-4/IGHV3-20/IGHV3-7/IGHV3-15/IGHV1-45/JCHAIN/IGHV3-72/IGHV3-48/IGHV2-26/IGHV3-49/IGHV4-28/IGHV5-51/IGHV1-69/IGHV4-39/IGHV4-59/IGHA2/IGHV3-21/IGHV3-13/IGHV1-18*	23

(*Continued*)

 © 2023. The Author(s).

Global Medical Genetics © 2023. The Author(s).

Supplementary Table S1 (*Continued*)

Ontology	ID	Description	GeneRatio	BgRatio	p-Value	*p*.adjust	q-Value	geneID	Count
MF	GO:0005261	Cation channel activity	50/906	345/18,410	6.29E-12	6.36E-10	5.30E-10	*CHRNB3/KCNK3/SCNN1B/P2RX1/PEX5L/KCNJ5/OTOP3/KCNA2/KCNAB1/TRPM6/KCNJ16/KCNH7/SCN3A/CACNB2/KCNB1/KCNA3/SCN4A/UNC80/KCND3/CHRNB2/SCNN1G/GRIK5/ABCC9/KCNV1/MCOLN3/KCNJ11/CACNA2D2/SCN3B/CNGA3/SCN7A/TRPM3/TMEM63C/CACNA1B/CACNG5/KCNA5/KCNJ3/KCNMB2/GRIA2/HCN4/CNR1/SNAP25/KCNB2/KCNG3/GRIA4/ABCC8/GPM6A/TRPV6/KCNH6/KCNJ6/KCNK16*	50
MF	GO:0015276	Ligand-gated ion channel activity	28/906	145/18,410	4.26E-10	3.45E-08	2.87E-08	*CHRNB3/SCNN1B/P2RX1/PEX5L/KCNJ5/KCNJ16/GABRB3/KCNH7/CHRNB2/SCNN1G/GRIK5/ABCC9/MCOLN3/KCNJ11/CNGA3/GLRA3/KCNJ3/GRIA2/HCN4/GRIA4/GABRA4/GABRG2/ABCC8/KCNH6/CFTR/KCNJ6/GLRA1/GABRA2*	28
MF	GO:0022834	Ligand-gated channel activity	28/906	145/18,410	4.26E-10	3.45E-08	2.87E-08	*CHRNB3/SCNN1B/P2RX1/PEX5L/KCNJ5/KCNJ16/GABRB3/KCNH7/CHRNB2/SCNN1G/GRIK5/ABCC9/MCOLN3/KCNJ11/CNGA3/GLRA3/KCNJ3/GRIA2/HCN4/GRIA4/GABRA4/GABRG2/ABCC8/KCNH6/CFTR/KCNJ6/GLRA1/GABRA2*	28
MF	GO:0005249	Voltage-gated potassium channel activity	21/906	91/18,410	2.26E-09	1.66E-07	1.39E-07	*KCNK3/KCNJ5/KCNA2/KCNAB1/KCNJ16/KCNH7/KCNB1/KCNA3/KCND3/ABCC9/KCNV1/KCNJ11/KCNA5/KCNJ3/HCN4/SNAP25/KCNB2/KCNG3/ABCC8/KCNH6/KCNJ6*	21
MF	GO:0005244	Voltage-gated ion channel activity	32/906	201/18,410	4.00E-09	2.34E-07	1.95E-07	*KCNK3/KCNJ5/KCNA2/KCNAB1/KCNJ16/KCNH7/SCN3A/CACNB2/KCNB1/KCNA3/SCN4A/KCND3/ABCC9/KCNV1/KCNJ11/CACNA2D2/SCN3B/SCN7A/CACNA1B/CACNG5/KCNA5/KCNJ3/HCN4/CNR1/SNAP25/KCNB2/KCNG3/ABCC8/KCNH6/KCNJ6/CLIC3/KCNK16*	32
MF	GO:0022832	Voltage-gated channel activity	32/906	201/18,410	4.00E-09	2.34E-07	1.95E-07	*KCNK3/KCNJ5/KCNA2/KCNAB1/KCNJ16/KCNH7/SCN3A/CACNB2/KCNB1/KCNA3/SCN4A/KCND3/ABCC9/KCNV1/KCNJ11/CACNA2D2/SCN3B/SCN7A/CACNA1B/CACNG5/KCNA5/KCNJ3/HCN4/CNR1/SNAP25/KCNB2/KCNG3/ABCC8/KCNH6/KCNJ6/CLIC3/KCNK16*	32
MF	GO:0005267	Potassium channel activity	24/906	121/18,410	4.29E-09	2.34E-07	1.95E-07	*KCNK3/KCNJ5/KCNA2/KCNAB1/KCNJ16/KCNH7/KCNB1/KCNA3/KCND3/GRIK5/ABCC9/KCNV1/KCNJ11/KCNA5/KCNJ3/KCNMB2/HCN4/SNAP25/KCNB2/KCNG3/ABCC8/KCNH6/KCNJ6/KCNK16*	24
MF	GO:0008236	Serine-type peptidase activity	31/906	191/18,410	4.35E-09	2.34E-07	1.95E-07	*CTRL/CELA2B/CELA2A/CTRC/CELA3B/F11/CELA3A/RELN/PRSS1/CTRB2/CTRB1/CMA1/LPA/DPP10/KLKB1/PRSS2/TLL1/KLK1/CTSG/HPN/PCSK1/DPP6/PCSK2/*	31

Supplementary Table S1 (Continued)

Ontology	ID	Description	GeneRatio	BgRatio	p-Value	*p*.adjust	q-Value	geneID	Count
								MMP11/GZMK/HABP2/KLK13/PRSS3/TMPRSS4/KLK2/KLK8	
MF	GO:0017171	Serine hydrolase activity	31/906	195/18,410	7.24E-09	3.66E-07	3.05E-07	*CTRL/CELA2B/CELA2A/CTRC/CELA3B/F11/CELA3A/RELN/PRSS1/CTRB2/CTRB1/CMA1/LPA/DPP10/KLKB1/PRSS2/TLL1/KLK1/CTSG/HPN/PCSK1/DPP6/PCSK2/MMP11/GZMK/HABP2/KLK13/PRSS3/TMPRSS4/KLK2/KLK8*	31
MF	GO:0022843	Voltage-gated cation channel activity	26/906	144/18,410	7.90E-09	3.76E-07	3.13E-07	*KCNK3/KCNJ5/KCNA2/KCNAB1/KCNJ16/KCNH7/CACNB2/KCNB1/KCNA3/KCND3/ABCC9/KCNV1/KCNJ11/CACNA2D2/CACNA1B/CACNG5/KCNA5/KCNJ3/HCN4/CNR1/SNAP25/KCNB2/KCNG3/ABCC8/KCNH6/KCNJ6*	26
MF	GO:0015081	Sodium ion transmembrane transporter activity	26/906	150/18,410	1.91E-08	8.57E-07	7.14E-07	*SLC1A2/SCNN1B/SLC5A9/SLC8A3/SLC8A2/SCN3A/SLC5A7/SLC6A4/SLC8A1/SLC5A4/SCN4A/SLC4A4/SLC4A10/SCNN1G/GRIK5/SLC17A4/SLC6A15/SLC17A1/SCN3B/SCN7A/SLC18A2/ATP1A3/SLC5A8/HCN4/SLC10A2/ATP4A*	26
MF	GO:0004252	Serine-type endopeptidase activity	28/906	174/18,410	2.93E-08	1.25E-06	1.04E-06	*CTRL/CELA2B/CELA2A/CTRC/CELA3B/F11/CELA3A/PRSS1/CTRB2/CTRB1/CMA1/LPA/KLKB1/PRSS2/TLL1/KLK1/CTSG/HPN/PCSK1/PCSK2/MMP11/GZMK/HABP2/KLK13/PRSS3/TMPRSS4/KLK2/KLK8*	28
MF	GO:0015079	Potassium ion transmembrane transporter activity	26/906	154/18,410	3.34E-08	1.35E-06	1.12E-06	*KCNK3/KCNJ5/KCNA2/KCNAB1/KCNJ16/KCNH7/KCNB1/KCNA3/KCND3/GRIK5/ABCC9/KCNV1/KCNJ11/ATP1A3/KCNA5/KCNJ3/KCNMB2/HCN4/SNAP25/KCNB2/KCNG3/ATP4A/ABCC8/KCNH6/KCNJ6/KCNK16*	26
MF	GO:0008528	G protein-coupled peptide receptor activity	25/906	148/18,410	6.05E-08	2.33E-06	1.94E-06	*GRPR/VIPR2/CX3CR1/SORCS1/UTS2R/GPR37/TACR1/NPY5R/GALR1/TSHR/CYSLTR1/CCR2/CCKBR/PRLHR/CXCR6/SSTR2/CCR5/AVPR1B/NPY1R/AGTR2/SSTR3/GLP1R/XCR1/CXCR5/SCTR*	25
MF	GO:0001653	Peptide receptor activity	25/906	154/18,410	1.35E-07	4.95E-06	4.12E-06	*GRPR/VIPR2/CX3CR1/SORCS1/UTS2R/GPR37/TACR1/NPY5R/GALR1/TSHR/CYSLTR1/CCR2/CCKBR/PRLHR/CXCR6/SSTR2/CCR5/AVPR1B/NPY1R/AGTR2/SSTR3/GLP1R/XCR1/CXCR5/SCTR*	25
MF	GO:0099094	Ligand-gated cation channel activity	21/906	115/18,410	1.76E-07	6.17E-06	5.14E-06	*CHRNB3/SCNN1B/P2RX1/PEX5L/KCNJ5/KCNJ16/KCNH7/CHRNB2/SCNN1G/GRIK5/ABCC9/MCOLN3/KCNJ11/CNGA3/KCNJ3/GRIA2/HCN4/GRIA4/ABCC8/KCNH6/KCNJ6*	21
MF	GO:0005539	Glycosaminoglycan binding	30/906	234/18,410	1.56E-06	5.25E-05	4.37E-05	*CEL/F11/RTN4RL1/REG3G/TGFBR3/REG1B/LPA/REG3A/SELP/VTN/REG1A/CTSG/APOH/SFRP1/COL25A1/TNXB/APLP1/SERPINA10/PTPRC/THBS4/*	30

(Continued)

Global Medical Genetics © 2023. The Author(s).

Global Medical Genetics © 2023. The Author(s).

Supplementary Table S1 (*Continued*)

Ontology	ID	Description	GeneRatio	BgRatio	p-Value	*p*.adjust	q-Value	geneID	Count
								HABP2/FGF10/VIT/EPYC/COL11A1/JCHAIN/PLA2G2D/SERPINA5/FGFBP1/ZG16	
MF	GO:0005242	Inward rectifier potassium channel activity	9/906	27/18,410	3.42E-06	0.000111	9.21E-05	*KCNJ5/KCNJ16/KCNH7/ABCC9/KCNJ11/KCNJ3/ABCC8/KCNH6/KCNJ6*	9
MF	GO:0005179	Hormone activity	19/906	122/18,410	7.88E-06	0.000245	0.000204	*SPX/GPHA2/KL/VIP/EPO/CARTPT/ENHO/GHRL/ADCYAP1/CHGB/IGF1/ADIPOQ/NPY/GUCA2A/TTR/PPY/UCN3/SST/IAPP*	19
MF	GO:0005201	Extracellular matrix structural constituent	23/906	172/18,410	1.26E-05	0.000378	0.000314	*UMOD/GP2/ENAM/LAMB4/MMRN1/SBSPON/VTN/COL21A1/TFPI2/COL25A1/TNXB/ABI3BP/COL14A1/OGN/COL7A1/FGA/COL19A1/ADIPOQ/FGB/COL11A1/COL17A1/MUC6/FGG*	23
MF	GO:0099095	Ligand-gated anion channel activity	7/906	18/18,410	1.35E-05	0.000389	0.000324	*GABRB3/GLRA3/GABRA4/GABRG2/CFTR/GLRA1/GABRA2*	7
MF	GO:0048018	Receptor ligand activity	46/906	489/18,410	2.01E-05	0.00056	0.000466	*SPX/C5/GPHA2/PDGFD/FGF12/KL/VIP/SEMA6D/EPO/CARTPT/FLRT2/GDF10/FASLG/NRG2/ENHO/EGF/GHRL/GDF7/ADCYAP1/REG1A/CTSG/SEMA3E/IL33/CCL14/TNFSF9/VEGFD/CXCL12/OGN/CHGB/SCG2/THBS4/FGF10/IGF1/ADIPOQ/NPY/GUCA2A/BMP5/TTR/CCL21/CXCL9/PPY/UCN3/SST/LEFTY1/IAPP/CCL19*	46
MF	GO:0022853	Active ion transmembrane transporter activity	29/906	254/18,410	2.24E-05	0.000604	0.000503	*SLC1A2/SLCO4C1/SLC5A9/SLC8A3/SLC8A2/SLC5A7/SLC6A4/SLC8A1/SLC5A4/SLCO1C1/SLC26A4/SLC4A4/SLC4A10/ATP2B2/SLC17A4/ABCC9/SLC6A15/SLC17A1/KCNJ11/SLC18A2/SLC22A9/ATP1A3/SLC5A8/SLC10A2/ATP4A/ABCC8/ATP13A4/CFTR/SLC26A9*	29
MF	GO:0016247	Channel regulator activity	20/906	143/18,410	2.36E-05	0.000616	0.000513	*AMIGO1/KCNAB1/GPLD1/NOS1/FGF12/WNK2/DPP10/FXYD2/HPCAL4/NRXN1/AMBP/ABCC9/KCNV1/SCN3B/CACNG5/DPP6/KCNMB2/PRKCB/CFTR/NPY*	20
MF	GO:0030546	Signaling receptor activator activity	46/906	496/18,410	2.86E-05	0.000724	0.000603	*SPX/C5/GPHA2/PDGFD/FGF12/KL/VIP/SEMA6D/EPO/CARTPT/FLRT2/GDF10/FASLG/NRG2/ENHO/EGF/GHRL/GDF7/ADCYAP1/REG1A/CTSG/SEMA3E/IL33/CCL14/TNFSF9/VEGFD/CXCL12/OGN/CHGB/SCG2/THBS4/FGF10/IGF1/ADIPOQ/NPY/GUCA2A/BMP5/TTR/CCL21/CXCL9/PPY/UCN3/SST/LEFTY1/IAPP/CCL19*	46
MF	GO:0015370	Solute:sodium symporter activity	13/906	72/18,410	4.34E-05	0.001065	0.000887	*SLC1A2/SLC5A9/SLC5A7/SLC6A4/SLC5A4/SLC4A4/SLC4A10/SLC17A4/SLC6A15/SLC17A1/SLC18A2/SLC5A8/SLC10A2*	13
MF	GO:0099106	Ion channel regulator activity	19/906	138/18,410	4.67E-05	0.001083	0.000902	*AMIGO1/KCNAB1/GPLD1/NOS1/FGF12/WNK2/DPP10/FXYD2/HPCAL4/NRXN1/AMBP/ABCC9/KCNV1/SCN3B/DPP6/KCNMB2/PRKCB/CFTR/NPY*	19

Supplementary Table S1 (*Continued*)

Ontology	ID	Description	GeneRatio	BgRatio	p-Value	*p*.adjust	q-Value	geneID	Count
MF	GO:0070492	Oligosaccharide binding	6/906	15/18,410	4.77E-05	0.001083	0.000902	*REG3G/REG1B/REG3A/SELP/REG1A/SELE*	6
MF	GO:0022804	Active transmembrane transporter activity	39/906	404/18,410	4.82E-05	0.001083	0.000902	*SLC1A2/ABCB1/ABCA3/SLC16A12/SLCO4C1/SLC6A16/SLC5A9/SLC8A3/SLC8A2/ABCD2/SLC5A7/SLC6A4/SLC8A1/SLC5A4/SLC38A3/SLCO1C1/SLC26A4/SLC4A4/SLC4A10/ATP2B2/SLC17A4/ABCC9/SLC6A15/SLC17A1/SLC29A4/KCNJ11/ABCA8/SLC18A2/SLC22A9/SLC38A4/ATP1A3/SLC5A8/SLC10A2/ATP4A/ABCC8/ATP13A4/CFTR/SLC6A17/SLC26A9*	39
MF	GO:0044325	Transmembrane transporter binding	18/906	127/18,410	4.98E-05	0.001088	0.000906	*KCNAB1/NOS1/AKAP6/FGF12/DPP10/KCNB1/SLC8A1/PIRT/KCND3/ABCC9/ANK2/LRRK2/KCNJ11/SCN3B/RIMS2/RIMS1/CASR/ABCC8*	18
MF	GO:0008188	Neuropeptide receptor activity	10/906	45/18,410	5.25E-05	0.001118	0.000931	*GRPR/SORCS1/GPR37/TACR1/NPY5R/GALR1/PRLHR/SSTR2/NPY1R/SSTR3*	10
MF	GO:0015291	Secondary active transmembrane transporter activity	27/906	240/18,410	5.49E-05	0.00114	0.000949	*SLC1A2/SLC16A12/SLCO4C1/SLC6A16/SLC5A9/SLC8A3/SLC8A2/SLC5A7/SLC6A4/SLC8A1/SLC5A4/SLC38A3/SLCO1C1/SLC26A4/SLC4A4/SLC4A10/SLC17A4/SLC6A15/SLC17A1/SLC18A2/SLC22A9/SLC38A4/ATP1A3/SLC5A8/SLC10A2/SLC6A17/SLC26A9*	27
MF	GO:0008201	Heparin binding	21/906	168/18,410	8.09E-05	0.001635	0.001362	*CEL/F11/RTN4RL1/TGFBR3/LPA/SELP/VTN/CTSG/APOH/SFRP1/COL25A1/TNXB/APLP1/SERPINA10/PTPRC/THBS4/FGF10/COL11A1/PLA2G2D/SERPINA5/FGFBP1*	21
MF	GO:0019957	C-C chemokine binding	7/906	24/18,410	0.000113	0.002231	0.001858	*CX3CR1/CCR2/CXCR6/CCR5/ACKR1/XCR1/CXCR5*	7
MF	GO:0042923	Neuropeptide binding	8/906	32/18,410	0.000122	0.002351	0.001958	*GRPR/GPR37/NPY5R/GALR1/PRLHR/SSTR2/NPY1R/SSTR3*	8
MF	GO:1901681	Sulfur compound binding	28/906	267/18,410	0.000138	0.002592	0.002159	*GNMT/CEL/ACACB/P2RX1/F11/RTN4RL1/TGFBR3/ACADL/LPA/SELP/VTN/OGDHL/CTSG/APOH/SFRP1/COL25A1/TNXB/APLP1/SERPINA10/CBS/PTPRC/THBS4/FGF10/GLRA1/COL11A1/PLA2G2D/SERPINA5/FGFBP1*	28
MF	GO:0022824	Transmitter-gated ion channel activity	11/906	60/18,410	0.000144	0.002594	0.00216	*CHRNB3/GABRB3/CHRNB2/GRIK5/GLRA3/GRIA2/GRIA4/GABRA4/GABRG2/GLRA1/GABRA2*	11
MF	GO:0022835	Transmitter-gated channel activity	11/906	60/18,410	0.000144	0.002594	0.00216	*CHRNB3/GABRB3/CHRNB2/GRIK5/GLRA3/GRIA2/GRIA4/GABRA4/GABRG2/GLRA1/GABRA2*	11
MF	GO:0019956	Chemokine binding	8/906	33/18,410	0.000154	0.00268	0.002231	*CX3CR1/CCR2/ITGA4/CXCR6/CCR5/ACKR1/XCR1/CXCR5*	8

(*Continued*)

 © 2023. The Author(s).

Supplementary Table S1 (*Continued*)

Ontology	ID	Description	GeneRatio	BgRatio	p-Value	*p*.adjust	q-Value	geneID	Count
MF	GO:0042834	Peptidoglycan binding	6/906	18/18,410	0.000156	0.00268	0.002231	*REG3G/REG1B/REG3A/REG1A/JCHAIN/ZG16*	6
MF	GO:0005230	Extracellular ligand-gated ion channel activity	12/906	73/18,410	0.000215	0.003631	0.003024	*CHRNB3/P2RX1/GABRB3/CHRNB2/GRIK5/GLRA3/GRIA2/GRIA4/GABRA4/GABRG2/GLRA1/GABRA2*	12
MF	GO:0015293	Symporter activity	18/906	144/18,410	0.000255	0.004208	0.003504	*SLC1A2/SLC16A12/SLC6A16/SLC5A9/SLC5A7/SLC6A4/SLC5A4/SLC38A3/SLC4A4/SLC4A10/SLC17A4/SLC6A15/SLC17A1/SLC18A2/SLC38A4/SLC5A8/SLC10A2/SLC6A17*	18
MF	GO:0017075	Syntaxin-1 binding	6/906	20/18,410	0.000299	0.004832	0.004024	*SYBU/SLC6A4/UNC13A/LRRK2/SNAP25/CPLX2*	6
MF	GO:0015108	Chloride transmembrane transporter activity	14/906	99/18,410	0.00034	0.005309	0.004421	*GABRB3/ANO5/SLC6A4/ANO3/SLC26A4/GLRA3/SLC18A2/GABRA4/GABRG2/CFTR/CLIC3/GLRA1/GABRA2/SLC26A9*	14
MF	GO:0031406	Carboxylic acid binding	20/906	173/18,410	0.000341	0.005309	0.004421	*GNMT/PLA2G1B/ACACB/NOS1/ALB/ST8SIA3/SELP/AFM/GLRA3/SELE/RBP2/SIGLEC6/GAD2/NR1H4/ADIPOQ/GLRA1/PMP2/FABP4/SERPINA5/PCK1*	20
MF	GO:0015271	Outward rectifier potassium channel activity	5/906	14/18,410	0.000394	0.006011	0.005005	*KCNA2/KCNB1/KCNA3/KCND3/KCNA5*	5
MF	GO:0015103	Inorganic anion transmembrane transporter activity	18/906	151/18,410	0.000458	0.00686	0.005713	*GABRB3/ANO5/SLC6A4/ANO3/SLC26A4/SLC4A4/SLC4A10/ABCC9/SLC17A1/GLRA3/SLC18A2/GABRA4/GABRG2/CFTR/CLIC3/GLRA1/GABRA2/SLC26A9*	18
MF	GO:0005237	Inhibitory extracellular ligand-gated ion channel activity	5/906	15/18,410	0.000567	0.008338	0.006943	*GLRA3/GABRA4/GABRG2/GLRA1/GABRA2*	5
MF	GO:0042277	Peptide binding	30/906	322/18,410	0.000626	0.00905	0.007536	*GRPR/VIPR2/PEX5L/NLGN1/CLU/CD36/CMA1/GPR37/NPY5R/PRLR/GALR1/CRHBP/ANPEP/TRHDE/CCKBR/COL25A1/PRLHR/MAPK8IP2/CACNA1B/SSTR2/ATP1A3/AVPR1B/TRAV29DV5/GRIA2/NPY1R/GRIA4/SSTR3/GLP1R/SCTR/IAPP*	30
MF	GO:0019905	Syntaxin binding	11/906	71/18,410	0.000659	0.009353	0.007788	*SYBU/SYT6/SLC6A4/UNC13A/LRRK2/STXBP5L/SYT9/SNAP25/CPLX2/SYT5/SYT8*	11
MF	GO:0016493	C–C chemokine receptor activity	6/906	23/18,410	0.000685	0.009559	0.00796	*CX3CR1/CCR2/CXCR6/CCR5/XCR1/CXCR5*	6
MF	GO:0004896	Cytokine receptor activity	13/906	97/18,410	0.00092	0.012458	0.010374	*LIFR/CX3CR1/FLT3/IL12RB2/PRLR/IL5RA/CCR2/GFRA1/CXCR6/CCR5/GFRA3/XCR1/CXCR5*	13
MF	GO:0008509	Anion transmembrane transporter activity	29/906	315/18,410	0.000924	0.012458	0.010374	*SLC1A2/SLC16A12/SLC7A2/SLCO4C1/CD36/GABRB3/ANO5/SLC6A4/ANO3/SLC38A3/SLCO1C1/SLC2A2/SLC26A4/SLC4A4/SLC4A10/ABCC9/SLC17A1/GLRA3/*	29

 © 2023. The Author(s).

Supplementary Table S1 (*Continued*)

Ontology	ID	Description	GeneRatio	BgRatio	p-Value	*p*.adjust	q-Value	geneID	Count
								SLC18A2/SLC22A9/SLC3A1/SLC5A8/GABRA4/GABRG2/CFTR/CLIC3/GLRA1/GABRA2/SLC26A9	
MF	GO:0005254	Chloride channel activity	11/906	74/18,410	0.000941	0.012479	0.010392	*GABRB3/ANO5/ANO3/GLRA3/GABRA4/GABRG2/CFTR/CLIC3/GLRA1/GABRA2/SLC26A9*	11
MF	GO:0000149	SNARE binding	14/906	110/18,410	0.000999	0.01304	0.010859	*SYBU/SYT6/KCNB1/SLC6A4/SYT4/UNC13A/LRRK2/STXBP5L/SYT9/SNAP25/SNAP91/CPLX2/SYT5/SYT8*	14
MF	GO:0042562	Hormone binding	12/906	87/18,410	0.001108	0.014223	0.011844	*VIPR2/MTNR1A/AMHR2/PRLR/GALR1/CRHBP/CCKBR/ATP1A3/AVPR1B/GLP1R/TTR/SCTR*	12
MF	GO:0005342	Organic acid transmembrane transporter activity	18/906	165/18,410	0.001304	0.016482	0.013725	*SLC1A2/ABCB1/SLC16A12/SLC7A2/CD36/SLC38A3/SLCO1C1/SLC26A4/SLC6A15/SLC7A14/SLC29A4/SLC38A11/SLC22A9/SLC3A1/SLC38A4/SLC5A8/SLC10A2/SLC26A9*	18
MF	GO:0001637	G protein-coupled chemoattractant receptor activity	6/906	26/18,410	0.001378	0.016886	0.014061	*CX3CR1/CCR2/CXCR6/CCR5/XCR1/CXCR5*	6
MF	GO:0004950	Chemokine receptor activity	6/906	26/18,410	0.001378	0.016886	0.014061	*CX3CR1/CCR2/CXCR6/CCR5/XCR1/CXCR5*	6
MF	GO:0042626	Atpase-coupled transmembrane transporter activity	12/906	90/18,410	0.001495	0.018055	0.015035	*ABCB1/ABCA3/ABCD2/ATP2B2/ABCC9/KCNJ11/ABCA8/ATP1A3/ATP4A/ABCC8/ATP13A4/CFTR*	12
MF	GO:0033218	Amide binding	34/906	402/18,410	0.001543	0.018359	0.015289	*GNMT/GRPR/ACACB/VIPR2/P2RX1/PEX5L/NLGN1/CLU/CD36/CMA1/ACADL/GPR37/NPY5R/PRLR/GALR1/CRHBP/ANPEP/TRHDE/CCKBR/COL25A1/PRLHR/MAPK8IP2/CACNA1B/SSTR2/ATP1A3/AVPR1B/TRAV29DV5/GRIA2/NPY1R/GRIA4/SSTR3/GLP1R/SCTR/IAPP*	34
MF	GO:0005272	Sodium channel activity	8/906	46/18,410	0.001646	0.019297	0.01607	*SCNN1B/SCN3A/SCN4A/SCNN1G/GRIK5/SCN3B/SCN7A/HCN4*	8
MF	GO:0015294	Solute:cation symporter activity	13/906	104/18,410	0.001763	0.020371	0.016964	*SLC1A2/SLC5A9/SLC5A7/SLC6A4/SLC5A4/SLC4A4/SLC4A10/SLC17A4/SLC6A15/SLC17A1/SLC18A2/SLC5A8/SLC10A2*	13
MF	GO:0005326	Neurotransmitter transmembrane transporter activity	5/906	19/18,410	0.001863	0.020641	0.017189	*SLC6A16/SLC6A4/SLC18A3/SLC6A15/SLC29A4*	5
MF	GO:0030506	Ankyrin binding	5/906	19/18,410	0.001863	0.020641	0.017189	*NRCAM/SPTB/SLC8A1/KCNJ11/PTPRC*	5
MF	GO:0051428	Peptide hormone receptor binding	5/906	19/18,410	0.001863	0.020641	0.017189	*GPHA2/VIP/ADCYAP1/GNAO1/UCN3*	5
MF	GO:0030276	Clathrin binding	10/906	69/18,410	0.001927	0.02107	0.017545		10

(*Continued*)

 © 2023. The Author(s).

Supplementary Table S1 (*Continued*)

Ontology	ID	Description	GeneRatio	BgRatio	p-Value	*p*.adjust	q-Value	geneID	Count
								SYT6/DNAJC6/NSG2/SYT4/LRRK2/SYT9/SNAP91/SYT5/CALY/SYT8	
MF	GO:0030165	PDZ domain binding	11/906	82/18,410	0.002208	0.023815	0.019832	*SNTG2/NLGN1/TGFBR3/CXXC4/GPR37/PDZK1/GRIK5/ATP2B2/SHISA9/SSTR2/CFTR*	11
MF	GO:0022851	GABA-gated chloride ion channel activity	4/906	13/18,410	0.002915	0.030514	0.02541	*GABRB3/GABRA4/GABRG2/GABRA2*	4
MF	GO:0001614	Purinergic nucleotide receptor activity	5/906	21/18,410	0.003004	0.030514	0.02541	*P2RX1/P2RY12/P2RY13/GPR34/GPR87*	5
MF	GO:0016502	Nucleotide receptor activity	5/906	21/18,410	0.003004	0.030514	0.02541	*P2RX1/P2RY12/P2RY13/GPR34/GPR87*	5
MF	GO:0005184	Neuropeptide hormone activity	6/906	30/18,410	0.003004	0.030514	0.02541	*SPX/VIP/CARTPT/ADCYAP1/NPY/PPY*	6
MF	GO:0046943	Carboxylic acid transmembrane transporter activity	17/906	164/18,410	0.003017	0.030514	0.02541	*SLC1A2/ABCB1/SLC16A12/SLC7A2/CD36/SLC38A3/SLCO1C1/SLC26A4/SLC6A15/SLC7A14/SLC38A11/SLC22A9/SLC3A1/SLC38A4/SLC5A8/SLC10A2/SLC26A9*	17
MF	GO:0030594	Neurotransmitter receptor activity	13/906	111/18,410	0.00316	0.031561	0.026282	*CHRNB3/GABRB3/CHRM3/CHRNB2/GRIK5/GLRA3/CHRM2/GRIA2/GRIA4/GABRA4/GABRG2/GLRA1/GABRA2*	13
MF	GO:0005253	Anion channel activity	11/906	87/18,410	0.003537	0.03455	0.028771	*GABRB3/ANO5/ANO3/GLRA3/GABRA4/GABRG2/CFTR/CLIC3/GLRA1/GABRA2/SLC26A9*	11
MF	GO:0030020	Extracellular matrix structural constituent conferring tensile strength	7/906	41/18,410	0.003545	0.03455	0.028771	*COL21A1/COL25A1/COL14A1/COL7A1/COL19A1/COL11A1/COL17A1*	7
MF	GO:0017046	Peptide hormone binding	8/906	52/18,410	0.003662	0.035102	0.029231	*VIPR2/PRLR/GALR1/CRHBP/CCKBR/AVPR1B/GLP1R/SCTR*	8
MF	GO:0015106	Bicarbonate transmembrane transporter activity	5/906	22/18,410	0.003732	0.035102	0.029231	*SLC26A4/SLC4A4/SLC4A10/CFTR/SLC26A9*	5
MF	GO:0033691	Sialic acid binding	5/906	22/18,410	0.003732	0.035102	0.029231	*ST8SIA3/SELP/SELE/SIGLEC6/ADIPOQ*	5
MF	GO:0030246	Carbohydrate binding	24/906	270/18,410	0.003846	0.035748	0.029769	*REG3G/REG1B/REG3A/ATRNL1/DCDC1/SELP/VTN/GALNT16/FCN2/CLEC9A/REG1A/NOMO3/CNTN2/FREM1/SELE/LGALS2/CLEC10A/CRYBG2/SIGLEC6/GCK/CLEC17A/KRT1/APCS/ZG16*	24
MF	GO:0045028	G protein-coupled purinergic nucleotide receptor activity	4/906	14/18,410	0.003923	0.035748	0.029769	*P2RY12/P2RY13/GPR34/GPR87*	4

 © 2023. The Author(s).

Supplementary Table S1 (*Continued*)

Ontology	ID	Description	GeneRatio	BgRatio	p-Value	*p*.adjust	q-Value	geneID	Count
MF	GO:0098960	Postsynaptic neurotransmitter receptor activity	9/906	64/18,410	0.003933	0.035748	0.029769	*CHRNB3/CHRM3/CHRNB2/GRIK5/CHRM2/GABRA4/GABRG2/GLRA1/GABRA2*	9
MF	GO:0019955	Cytokine binding	15/906	141/18,410	0.004043	0.036346	0.030267	*LIFR/CX3CR1/CD36/IL12RB2/TGFBR3/PRLR/KIT/IL5RA/CCR2/ITGA4/CXCR6/CCR5/ACKR1/XCR1/CXCR5*	15
MF	GO:0051427	Hormone receptor binding	6/906	32/18,410	0.004217	0.037492	0.031221	*GPHA2/VIP/ADCYAP1/GNAO1/FABP4/UCN3*	6
MF	GO:0015459	Potassium channel regulator activity	8/906	54/18,410	0.004646	0.04085	0.034018	*AMIGO1/KCNAB1/WNK2/DPP10/ABCC9/KCNV1/DPP6/KCNMB2*	8
MF	GO:0004175	Endopeptidase activity	34/906	432/18,410	0.004861	0.042289	0.035215	*CTRL/CELA2B/CELA2A/CTRC/CELA3B/MMP24/F11/CELA3A/PRSS1/CTRB2/CTRB1/CMA1/LPA/KLKB1/PRSS2/TLL1/USP2/KLK1/CTSG/SFRP1/HPN/PCSK1/PCSK2/MMP11/GZMK/HABP2/KLK13/PRSS3/PAPPA2/MEP1B/TMPRSS4/KLK2/KLK8/CASP14*	34
MF	GO:0005251	Delayed rectifier potassium channel activity	6/906	33/18,410	0.004944	0.042548	0.035431	*KCNA2/KCNB1/KCNA3/KCNA5/KCNB2/KCNG3*	6
MF	GO:0005200	Structural constituent of cytoskeleton	12/906	104/18,410	0.005052	0.042901	0.035725	*EPB41L4B/SORBS2/SPTB/DMD/PRPH/ANK2/INA/CTNNA2/TUBB4A/ACTL6B/KRT16/KRT6B*	12
MF	GO:0042043	Neurexin family protein binding	4/906	15/18,410	0.005144	0.042901	0.035725	*CEL/NLGN1/LRRTM2/CPE*	4
MF	GO:0086008	Voltage-gated potassium channel activity involved in cardiac muscle cell action potential repolarization	4/906	15/18,410	0.005144	0.042901	0.035725	*KCNJ5/KCND3/KCNA5/KCNJ3*	4
MF	GO:0019829	Atpase-coupled cation transmembrane transporter activity	7/906	44/18,410	0.005319	0.043908	0.036564	*ATP2B2/ABCC9/KCNJ11/ATP1A3/ATP4A/ABCC8/ATP13A4*	7
MF	GO:0005248	Voltage-gated sodium channel activity	5/906	24/18,410	0.005552	0.044914	0.037402	*SCN3A/SCN4A/SCN3B/SCN7A/HCN4*	5
MF	GO:0019865	Immunoglobulin binding	5/906	24/18,410	0.005552	0.044914	0.037402	*UMOD/AMBP/MS4A2/JCHAIN/MS4A1*	5

 © 2023. The Author(s).
